# Anticoronavirus Activity of Uridine Glycoconjugates Containing a 1,2,3-Triazole Moiety

**DOI:** 10.1021/acs.jmedchem.5c01602

**Published:** 2025-08-08

**Authors:** Malgorzata Graul, Gabriela Brzuska, Ewa Wisniewska, Monika Dominska, Petra Strakova, Daniel Ruzek, Gabriela Pastuch-Gawolek, Ewelina Krol

**Affiliations:** † Laboratory of Recombinant Vaccines, Intercollegiate Faculty of Biotechnology, 49646 University of Gdansk and Medical University of Gdansk, Abrahama 58, Gdansk 80-307, Poland; ‡ Laboratory of Virus Molecular Biology, Intercollegiate Faculty of Biotechnology, University of Gdansk and Medical University of Gdansk, Abrahama 58, Gdansk 80-307, Poland; § Department of Organic Chemistry, Bioorganic Chemistry and Biotechnology, Faculty of Chemistry, Silesian University of Technology, Krzywoustego 4, Gliwice 44-100, Poland; ∥ Biotechnology Center, Silesian University of Technology, Krzywoustego 8, Gliwice 44-100, Poland; ⊥ Laboratory of Emerging Viral Diseases, 48357 Veterinary Research Institute, Hudcova 70, Brno CZ-62100, Czech Republic; # Department of Experimental Biology, Faculty of Science, Masaryk University, Kamenice 735, Brno CZ-62500, Czech Republic; ∇ Laboratory of Emerging Viral Infections, Veterinary Research Institute, Hudcova 296, Brno CZ-62100, Czech Republic

## Abstract

Coronaviruses can spread rapidly to new host species and cause severe respiratory and enteric diseases in vertebrates, including humans. To date, seven coronaviruses have been identified in humans, with severe acute respiratory syndrome coronavirus 2 (SARS-CoV-2) being the most notorious due to its substantial social and economic impact. Although anti-SARS-CoV-2 vaccines are available, infections remain widespread, highlighting the ongoing need for antiviral treatments. Here, we report the synthesis and evaluation of the activity of uridine glycoconjugates, designed as glycosyltransferase donor-type inhibitors incorporating a 1,2,3-triazole moiety. These compounds were tested against two model coronaviruses: murine hepatitis virus strain A59 (MHV) and human coronavirus strain NL63 (HCoV-NL63). Four of the synthesized compounds demonstrated strong antiviral activity against both viruses, and their efficacy was further confirmed against SARS-CoV-2. Our results suggest that these compounds interfere with the coronavirus infectivity and replication process. Thus, these novel compounds may prove to be effective broad-spectrum antiviral inhibitors.

## Graphical abstract

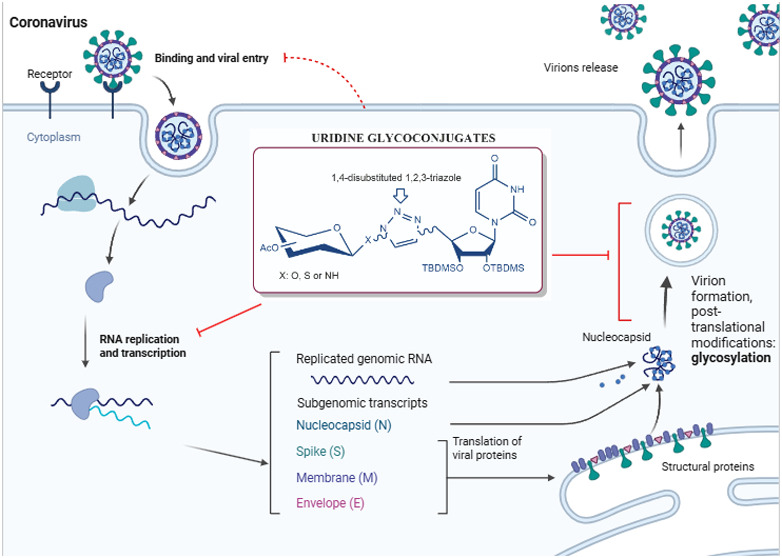

## 1 Introduction

Coronaviruses (CoVs) are large, enveloped, single-stranded RNA viruses that infect a variety of vertebrates, including humans, and are responsible for both respiratory and enteric diseases. They are classified into four genera: α-, β-, γ-, and delta-coronaviruses. To date, seven CoVs have been identified in humans. Among them, the α-CoVs HCoV-229E and HCoV-NL63, along with the β-CoVs HCoV-OC43 and HCoV-HKU1, are generally associated with mild respiratory infections, though more severe cases can occur.^1,2^ Three highly pathogenic β-CoVs—SARS-CoV, MERS-CoV, and SARS-CoV-2—are known to cause severe respiratory and systemic infections, presenting significant public health risks.^3^

The SARS-CoV-2 pandemic, which began in December 2019, has resulted in more than 600 million infections and more than 7 million deaths globally, resulting in profound social and economic consequences. Due to the biosafety requirements for handling SARS-CoV-2 (biosafety level 3 laboratory), antiviral studies often rely on model viruses, such as murine hepatitis virus (MHV) and human HCoV-NL63, which offer valuable insights into CoV replication cycle and antiviral efficacy.^4–8^ MHV, a β-CoV like SARS-CoV-2, infects mice and shares several characteristics with human-pathogenic CoVs. Although evolutionarily more distant, HCoV-NL63 uses the same ACE2 receptor for cellular entry as SARS-CoV-2, making it a valuable model for research. In response to the SARS-CoV-2 pandemic, a wide range of vaccines employing various mechanisms, including mRNA, DNA, and viral vector approaches, have been developed. However, FDA-approved antiviral treatments remain limited, with only remdesivir (Veklury) and baricitinib (Olumiant) authorized, along with emergency-use therapies such as molnupiravir (Lagevrio) and Paxlovid.^9^ As the need for new broad-spectrum antivirals remains critical, targeting multiple steps of the viral life cycle is expected to be a key strategy, especially in light of the potential for future CoV outbreaks.

The spike (S) glycoprotein of all coronaviruses is highly glycosylated, with more than 20 predicted *N*-glycosylation sites in a monomeric protein.^10–13^ It was confirmed by mass spectrometry that SARS-CoV-2 S protein contains 3 *O*-glycosylation sites and 22 *N*-glycosylation sites.^11,14^ The predominant types of *N*-glycans are complex and hybrid, with only 8 sites bearing oligomannose-type glycans. The glycosylation of other structural proteins of SARS-CoV-2 has not yet been studied. Notably, glycosylation of the S protein, mediated by host cell glycosyltransferases (GTs), plays a critical role in proper protein folding and interaction with the ACE2 receptor.^15^ Inhibitors that mimic natural GT substrates offer promising antiviral potential. Natural GT donor-type substrates typically consist of a sugar unit linked to uridine via a diphosphate moiety in their structure, which interacts with a bivalent metal ion present in the active center of the enzyme.^16,17^ Based on this, we designed and synthesized a panel of uridine-based glycoconjugates as GT inhibitors, replacing the diphosphate group with a noncharged linker capable of coordinating with the divalent metal ions necessary for GT function. This study evaluates the antiviral activity of these compounds against MHV, HCoV-NL63 and SARS-CoV-2, identifying four with potent activity.

## 2 Results

### 2.1 Synthesis of Glycoconjugates Type I–VI

Taking into account previous findings regarding the influence of structural modifications in uridine glycoconjugates on their activity,^18–20^ we aimed to explore how further structural modifications to the linker between sugar and uridine might affect the antiviral activity of these compounds against selected coronaviruses.

To this end, six types of uridine glycoconjugates (Figure 1) containing a 1,2,3-triazole ring in the linker structure were synthesized. These compounds differ in the type of heteroatom at the anomeric position of the sugar (oxygen for type **I** or **II**, nitrogen for type **III** and sulfur for types **IV**–**VI**). Additionally, the glycoconjugates vary in the nature of the linker bonds: type **I** contains only an ether bond, type **II** has an ether and an amide bond, type **III** includes two amide bonds, type **IV** has a thioether bond, type **V** features both a thioether and an amide bond, and type **VI** contains a thioether and an ether bond. The presence of heteroatoms like oxygen or sulfur and an amide bond, common in many biologically active compounds, is expected to enhance the chelating ability of these uridine glycoconjugates, thereby increasing their affinity for metal-dependent glycosyltransferases.^21^ Additionally, the orientation of the 1,2,3-triazole ring, formed by the reaction of the azides with propargyl derivatives of both linked parts, varies among the compounds. The sugars used for the glycoconjugating uridine are d-glucose and d-galactose derivatives, allowing us to assess the impact of the sugar moiety type on the activity and selectivity of the obtained conjugates. All synthesized uridine glycoconjugates contain a sugar part protected by acetyl groups, and hydroxyl groups in uridine protected by hydrophobic *tert*-butyldimethylsilyl groups (TBDMS). The same protecting groups were used in previously described glycoconjugates that exhibited antiviral activity.^18–20^

**Figure 1:**
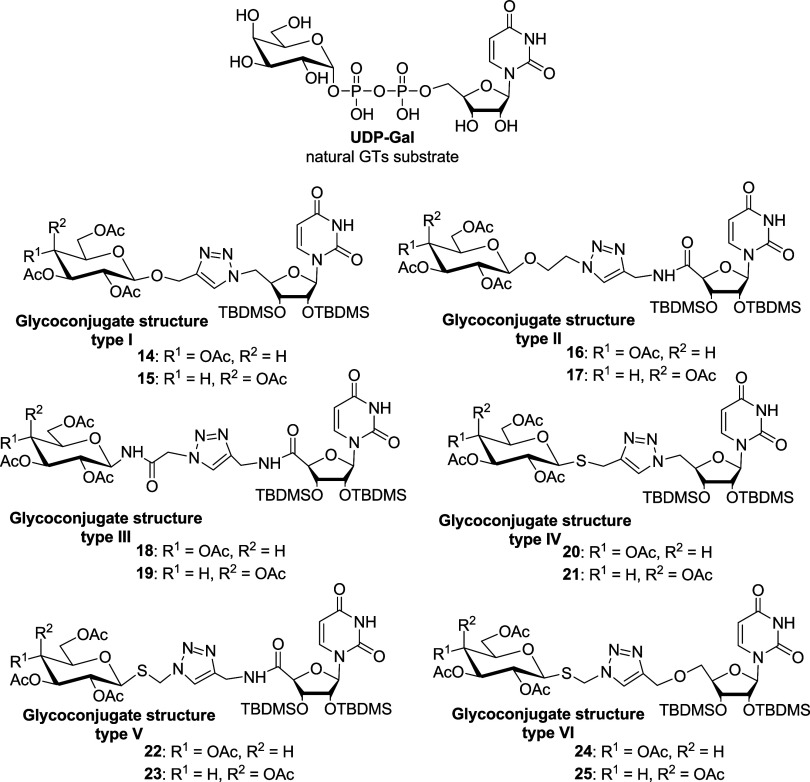
Structures of the tested glycoconjugates, analogues of the natural β4GalT substrate.

The key reaction used in the synthesis of the proposed glycoconjugate structures was the copper­(I)-catalyzed 1,3-dipolar azido-alkyne cycloaddition (CuAAC), which enables the connection of propargyl-containing derivatives with azide-containing derivatives to form a 1,2,3-triazole ring in the linker structure.^22^ The building blocks **1**–**13** required for this type of reaction were previously synthesized in our laboratory, and their synthesis has been described in earlier works.^19,23–29^

CuAAC reactions were performed at room temperature under an argon atmosphere to minimize the oxidation of copper­(I) ions generated *in situ* within the reaction mixture. The reactions were carried out for 24 to 72 h, using an equimolar ratio of reactants in a THF/*i*-PrOH/H_2_O solvent system. CuSO_4_ pentahydrate was used as the source of copper ions, while sodium ascorbate (NaAsc) served as the reducing agent to convert Cu­(II) ions to Cu­(I) ions. This method ensured the selective formation of 1,4-disubstituted 1,2,3-triazole derivatives (Scheme 1).

**Scheme 1:**
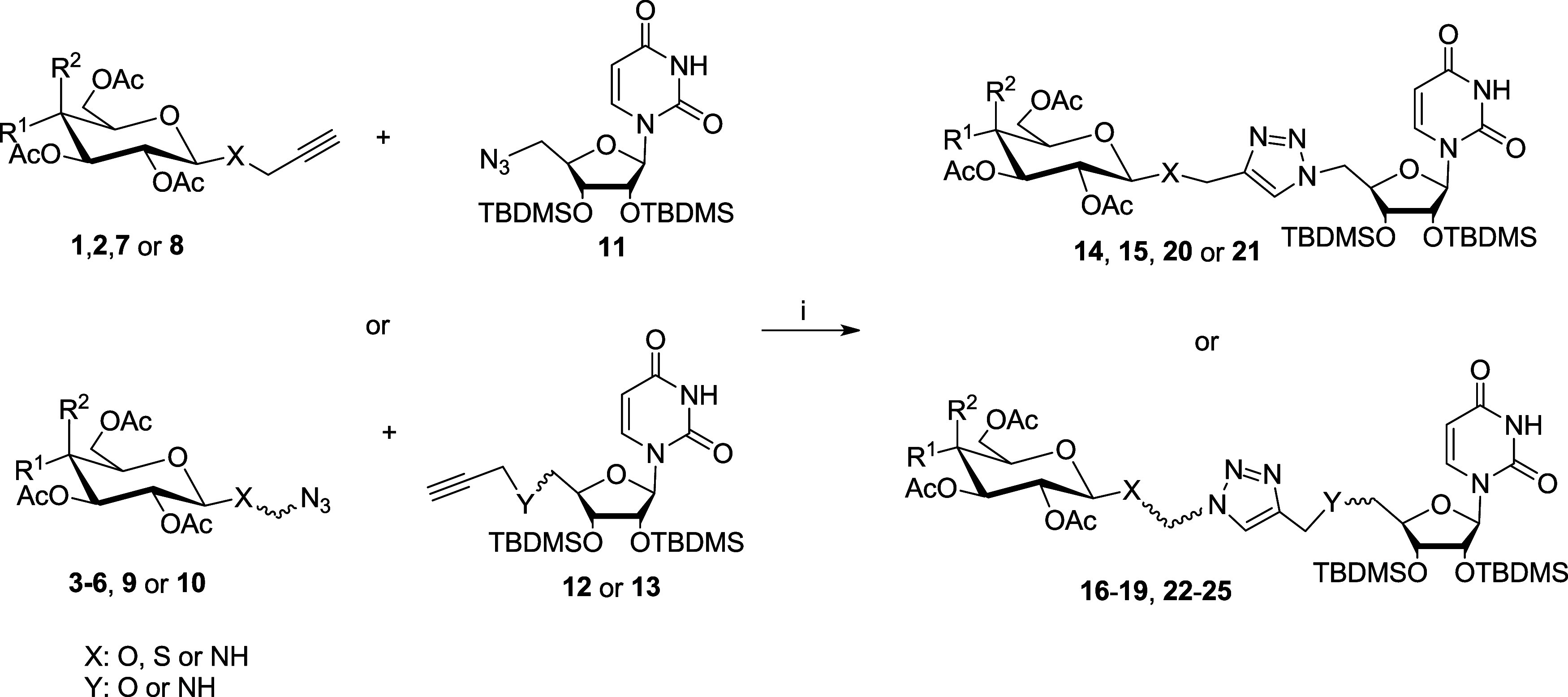
General Procedures for Glycoconjugate Synthesis^a^

a Reagents and Conditions: (i) CuSO_4_·5H_2_O, NaASC, THF/*i*-PrOH, H_2_O, r.t.

Glycoconjugates **14** and **15** (Type **I**) served as reference compounds for comparing the effects of subsequent structural modifications. In these glycoconjugates, the sugar is attached *via* an easily enzymatically hydrolyzed *O*-glycosidic bond, and apart from the 1,2,3-triazole ring, the linker structure does not contain any rigidifying elements. Glycoconjugates **16** and **17** (Type **II**) feature an amide moiety on the uridine side and a two-carbon alkyl chain on the sugar side to increase linker flexibility. Additionally, the 1,2,3-triazole ring was inversed compared to type **I** glycoconjugates. For glycoconjugates **18** and **19** (Type **III)**, an additional amide bond was introduced into the linker structure to increase its rigidity and enhance its ability to coordinate metal ions at the GT active center. Moreover, replacing the anomeric oxygen atom with nitrogen is expected to increase the resistance to enzymatic hydrolysis by intracellular glycosidases. To further improve hydrolytic stability, glycoconjugates **20–25** were synthesized with sulfur replacing oxygen at the anomeric position. Literature reports indicate that of 1-thiosugar derivatives are used in treating various human diseases^30^ and that sulfur-containing compounds can effectively complex metal ions.^31^ Depending on the combination of sugar derivative **7–10** and uridine derivative **11–13**, the glycoconjugates were designed with different orientations of the 1,2,3-triazole ring (glycoconjugates Type **IV** and **VI**), and varying functional groups—amide (Type **V**) or ether (Type **VI**) on the side of the junction with uridine.

### 2.2 Biological Evaluation

#### 2.2.1 Anticoronavirus Activity of Uridine Glycoconjugates

The antiviral activity of the designed compounds was initially screened against two coronaviruses: murine hepatitis virus strain A59 (MHV) and human coronavirus strain NL63 (HCoV-NL63). Since MHV infection in permissive LR7 cells at a multiplicity of infection (MOI) of 0.01 induces a pronounced cytopathic effect (CPE) 24 h postinfection, we used a CPE reduction assay to evaluate antiviral activity (Figure 2A). Both the protective efficacy of each compound (50 μM) against MHV infection, and its cytotoxicity in uninfected cells, was assessed using an MTS-based proliferation assay. At 50 μM, compounds **14**, **15**, **16**, **17**, **18**, **19**, **23**, **24**, and **25** reduced cell viability by 10–30%, while compounds **20**, **21**, and **22** showed no detectable toxicity. The tested compounds exhibited varying degrees of protection against MHV-induced CPE. For instance, compounds **18** and **19** (Type **III** glycoconjugates) demonstrated low cytotoxicity but also limited anti-MHV activity. In contrast, compounds **14**, **15** (Type **I**), **20**, **21** (Type **IV**), **22** (Type **V**), and **24** (Type **VI**) significantly reduced cell death in MHV-infected LR7 cells. Among these, compounds **20** and **21** were the most effective, nearly eliminating CPE with minimal compound-induced cytotoxicity.

**Figure 2:**
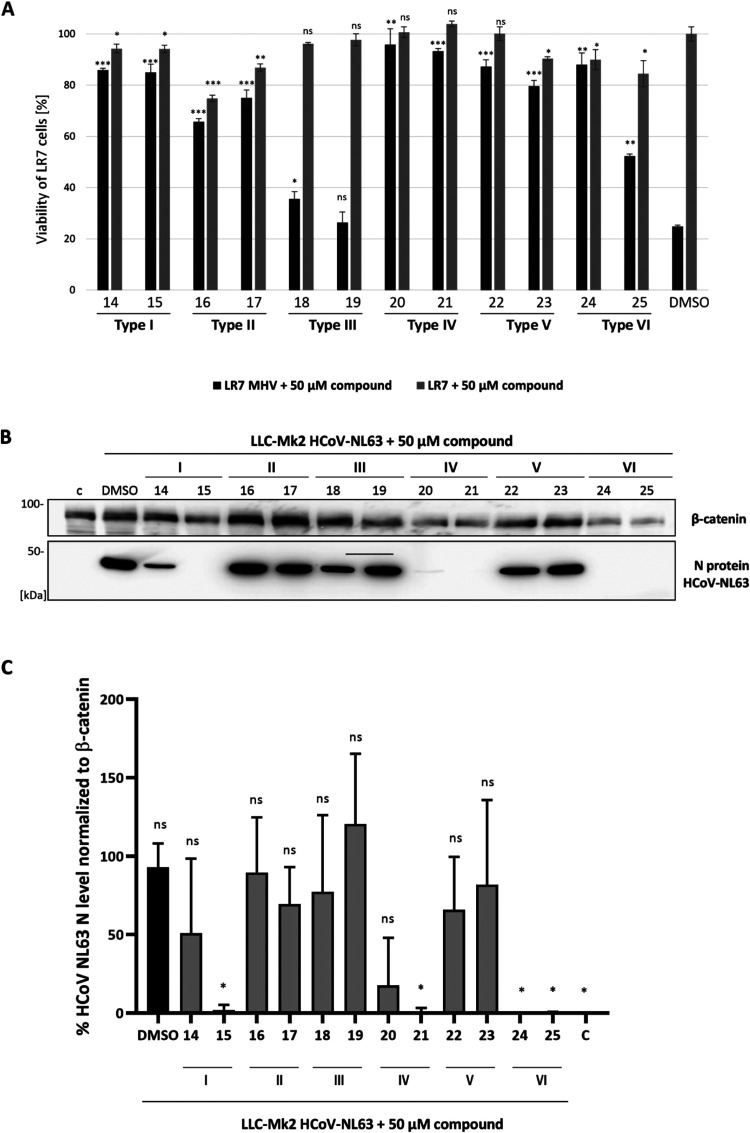
Analysis of anticoronavirus activity of compounds **14–25**. (A) Reduction of MHV cytotoxicity in LR7 cells following treatment with 50 μM of each compound. Gray bars indicate cell viability in the absence of viral infection; black bars indicate cell viability in MHV-infected LR7 cells. At 1 h postinfection (p.i.), virus-containing medium (MOI 0.01) was replaced with fresh medium containing 50 μM of each compound or DMSO. After 24 h, cell viability was measured via MTS/PMS assay. Results represent the mean of three independent experiments with error bars showing standard deviations. Statistical significance was assessed using a *t* test: *p < 0.05, **p < 0.005, ***p < 0.001; ns, not significant. (B) HCoV-NL63 N protein expression in LLC-Mk2 cells in the presence of 50 μM of each compound. LLC-Mk2 cells were infected with HCoV-NL63 at an MOI of 0.01. At 2 h p.i., the medium was replaced with a new one supplemented with 50 μM compound **14–25** or DMSO. Four days p.i. the cells were lysed and the expression of N HCoV-NL63 protein was determined by SDS-PAGE followed by immunoblotting with specific anti-N antibodies. The level of β-catenin was evaluated as a loading control. (C) Densitometric analysis of HCoV-NL63 N and β-catenin protein expression in LLC-Mk2 cells treated with 50 μM of each compound. The N protein levels were expressed as a percentage relative to DMSO-treated infected cells, while β-catenin levels were expressed as a percentage relative to noninfected control cells—C. The data are presented as the ratio of N to β-catenin for each sample, normalized accordingly. Results represent the mean of three independent experiments, with error bars indicating standard deviations. Statistical significance of N protein levels in treated infected cells, compared to DMSO-treated infected cells, was determined using one-way ANOVA followed by Dunnett’s posthoc test; *p < 0.1, ns – not significant.

Since HCoV-NL63 infection in LLC-Mk2 cells does not produce a pronounced CPE, antiviral activity of the compounds was assessed by analyzing Nucleocapsid (N) protein levels via Western blot and further densitometry analysis (Figure 2B,C). No significant reduction in N protein levels was observed in cells treated with Type II (compounds **16**, **17**), Type III (compounds **18**, **19**), or Type V (compounds **22**, **23**) glycoconjugates. In contrast, compounds from Types I (**14**, **15**), IV (**20**, **21**), and VI (**24**, **25**) significantly reduced the level of N protein production. No N protein was detected in the presence of compounds **15, 21, 24**, and **25**. Treatment with compound **20** resulted in the reduction of N, as well as β-catenin levels. Based on their strong antiviral activity against both MHV and HCoV-NL63 with their acceptable toxicity profiles, compounds **15**, **20**, **21**, and **24** were selected for further analysis. Compound **25** was excluded due to lower activity against MHV and high toxicity, while compound **14** was excluded for its weaker activity against HCoV-NL63.

#### 2.2.2 Dose-Dependent Activity of Selected Uridine Glycoconjugates against CoVs

To further evaluate antiviral activity, the dose-dependent effects of selected compounds against MHV, HCoV-NL63, and SARS-CoV-2 were examined. The compounds were tested over a wide range of concentrations by using CPE reduction assays for MHV and immunoblotting for HCoV-NL63 (Figure 3A,C). Viral titers in media from infected cells were also assessed by plaque assay (MHV and SARS-CoV-2) or foci assay (HCoV-NL63) (Figure 3B,D,E). All tested compounds showed a dose-dependent reduction in CPE or in viral titer. Notably, treatment with compounds **20** and **21** at concentrations above 6.25 μM, and with compounds **15** and **24** at concentrations above 12.5 μM, significantly reduced MHV-induced CPE in LR7 cells. Compounds **21** and **24** effectively decreased viral titers, with no detectable virus in cells treated with doses above 6.25 μM. Against HCoV-NL63, compound **24** showed the highest antiviral efficacy, completely inhibiting viral protein expression and viral release at doses above 6.25 μM, while compounds **15**, **20**, and **21** demonstrated slightly lower activity. Similarly, a dose-dependent antiviral effect was seen for all of the tested compounds against SARS-CoV-2 (Figure[Fig fig3]E).

**Figure 3:**
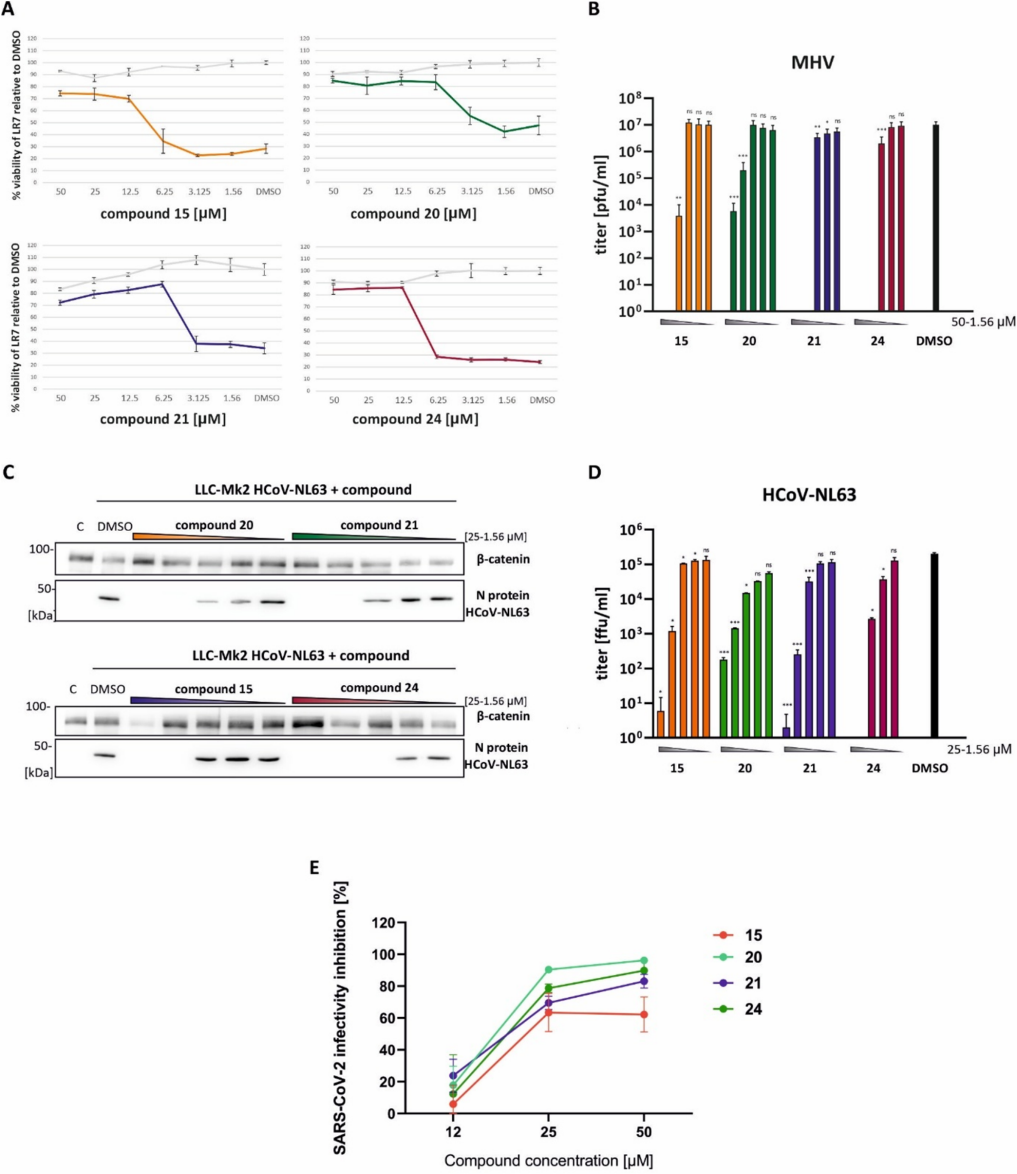
Antiviral activity of compounds 15, 20, 21, and 24 on MHV, HCoV-NL63, and SARS-CoV-2 infection. (A) LR7 cells were infected with MHV (MOI 0.01) and treated with compounds (0–50 μM) or DMSO. 24 h p.i. PMS/MTS test was performed. The values are presented as a percentage of the viability of cells treated with DMSO (100%). The gray lines illustrate the viability analysis of compound-treated noninfected LR7 cells and MHV-infected cells, respectively. (B) 24 h p.i. the medium sample was collected and MHV was titrated by a plaque assay. (C) LLC-Mk2 cells were infected with HCoV-NL63 at an MOI of 0.01 and treated with compounds (0–25 μM). Four days p.i. cells were collected and lysed. Expression of the HCoV-NL63 N protein and, as a control, β-catenin was evaluated by SDS-PAGE followed by immunoblotting with specific antibodies. C indicates noninfected LLC-Mk2 cells treated with DMSO for 4 days. (D) The medium sample was collected and HCoV-NL63 was titrated by a foci-forming assay. (E) Dose-dependent inhibition of SARS-CoV-2 replication by the compounds **15**, **20**, **21**, and **24**. Vero cells were infected with SARS-CoV-2 and 2 h after infection the cells were treated with different concentrations of the tested molecules. Culture supernatants were collected at 48 h after infection and analyzed for SARS-CoV-2 infectivity by plaque assay. Results represent the mean of three independent experiments with error bars showing standard deviations. Statistical significance was assessed by *t* test; * *p* < 0.05, ** *p* < 0.005, *** *p* < 0.001, ns – not significant.

Viral titration assays were used to determine the half-maximal inhibitory concentration (IC_50_) for each compound, defined as the concentration required to reduce the level of virus replication by 50%. Compounds cytotoxicity in LR7, LLC-Mk2 and Vero cells (0–100 μM) was evaluated using an MTS-based assay, and the cytotoxic concentrations (CC_50_) were estimated accordingly. The selectivity index (SI), calculated as the CC_50_/IC_50_ ratio, provided an indication of each compound’s therapeutic potential and safety (Table 1). Among the tested compounds, compound **24** showed the highest SI values against MHV and HCoV-NL63, while compound **20** demonstrated the highest SI against SARS-CoV-2.

**Table 1:** Comparison of the Anticoronaviral Activity (IC_50_) of the Compounds against MHV, HCoV-NL63, and SARS-CoV-2, along with Their Cytotoxicity (CC_50_) in LR7, LLC-Mk2, and Vero Cell Lines, and the Corresponding Selectivity Indexes (SI)^a^

		**MHV**			**HCoV-NL63**			**SARS-CoV-2**		
**type of uridine glycoconjugate**	**compound**	**CC**_**50**_ ^b^ **[μM]**	**IC**_**50**_ ^c^ **[μM]**	**SI** ^d^	**CC**_**50**_ **[μM]**	**IC**_**50**_ **[μM]**	**SI**	**CC**_**50**_ **[μM]**	**IC**_**50**_ **[μM]**	**SI**
**I**	**15**	134.5	7.6	17.7	49.8	6.8	7.3	>100	∼27.4	>3.6
**IV**	**20**	>100	11.3	>8.8	>100	5.5	>18.2	>100	∼16.2	>6.2
	**21**	65.3	4.5	14.5	46.7	5.7	8.2	>100	∼19.0	>5.3
**VI**	**24**	>100	3.7	>27.0	59.1	3.1	19.0	>100	∼18.7	>5.3

a Cytotoxicity was measured after 48 h in LR7 and/or 96 h in Vero or LLC-Mk2 cell lines.

b the concentration of compound needed to reduce the cells viability by 50% (CC_50_),

c the concentration of compound needed to reduce the virus titer released from the cells by 50% (IC_50_),

d SI—a CC_50_/IC_50_ ratio.

#### 2.2.3 Insights into the Mechanism of Action

##### 2.2.3.1 Inhibition of SARS-CoV-2 Entry

To investigate whether these compounds interfere with SARS-CoV-2 entry, a virus-like particle (SC-VLP) system was employed.^32^ SC-VLP consists of four SARS-CoV-2 structural proteins and mRNA carrying a luciferase gene. This system allows for the evaluation of how alterations in S protein glycosylation affect particle assembly and entry into permissive cells. Additionally, it excludes viral RNA replication, isolating potential inhibitory effects on virus entry. SC-VLPs were produced in the presence of each compound (3.125–12.5 μM) or DMSO. The internalization assay demonstrated a dose-dependent reduction in SC-VLP entry into HEK-hACE2 cells with all analyzed compounds (Figure 4A). Compounds **15** and **21** were particularly effective, with a 12.5 μM concentration reducing entry by 80% compared with the control. To determine whether this effect was due to reduced expression and secretion of S protein, Western blot analysis was performed on both SC-VLP-producing cells and the corresponding culture medium using S2-specific antibodies. The analysis revealed no significant changes in the levels of S2 and N proteins in the producing cells (Supporting Information, S37). However, some minor alterations in S2 domain levels were observed in the medium from cells treated with compounds **15**, **20**, and **24**, with a more pronounced reduction detected in samples treated with compound **21** (Figure 4B). These findings suggest that the tested glycoconjugates may alter the glycosylation profile of the SARS-CoV-2 S protein, thereby inhibiting its interaction with the ACE2 receptor (compounds **15**, **20**, and **24**) or assembly and secretion of SC-VLP (compound **21**).

**Figure 4:**
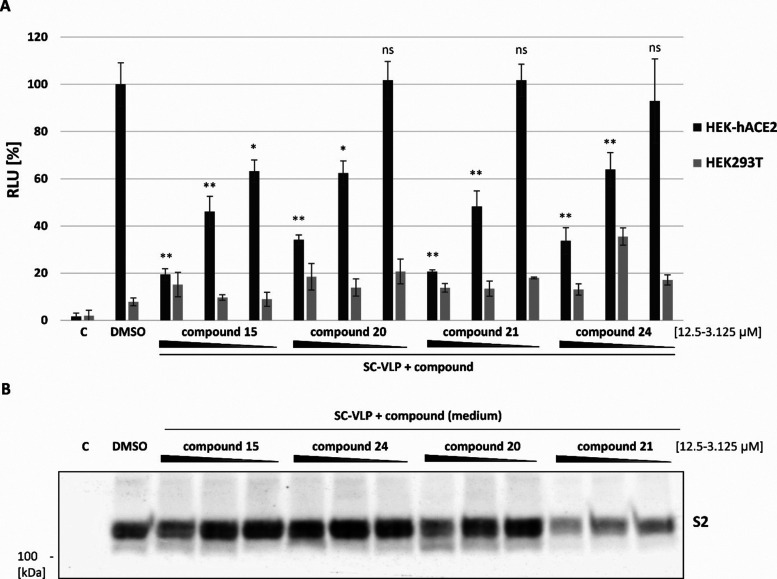
The effect of uridine glycoconjugates on SC-VLPs entry to HEK-hACE2 cells. (A) HEK293T cells were transfected with SC-VLPs components and treated with compounds **15**, **20**, **21**, and **24** (12.5–3.125 μM). Cell culture medium was used in the internalization assay using permissive HEK-hACE2 cell line and HEK293T as a negative control; C—medium from nontransfected HEK293T cells. Statistical significance was assessed using a *t* test: *p < 0.05, **p < 0.005, ***p < 0.001; ns, not significant. (B) Remaining cell culture medium was subjected to PEG-precipitation, followed by SDS-PAGE and immunoblotting with S2 domain-specific antibodies.

##### 2.2.3.2 Inhibition of Coronavirus Replication

Our previous studies have shown that uridine glycoconjugates can inhibit viral RNA replication, as demonstrated for hepatitis C virus.^33^ To evaluate the effect of the selected compounds on viral RNA synthesis, we analyzed viral RNA levels early in infection (8 h p.i.) in LR7 cells infected with MHV (MOI of 1) and treated with increasing concentrations of compounds **15**, **20**, **21**, and **24** (Figure 5A). Low concentrations (3.125–6.25 μM) of each compound allowed for MHV RNA detection, while higher concentrations resulted in undetectable viral RNA. Similarly, in HCoV-NL63-infected LLC-MK2 cells, 12.5 μM of compounds **15**and **21**, as well as 6.25 μM of compounds **20** and **24**, effectively suppressed viral RNA synthesis (Figure 5B).

**Figure 5:**
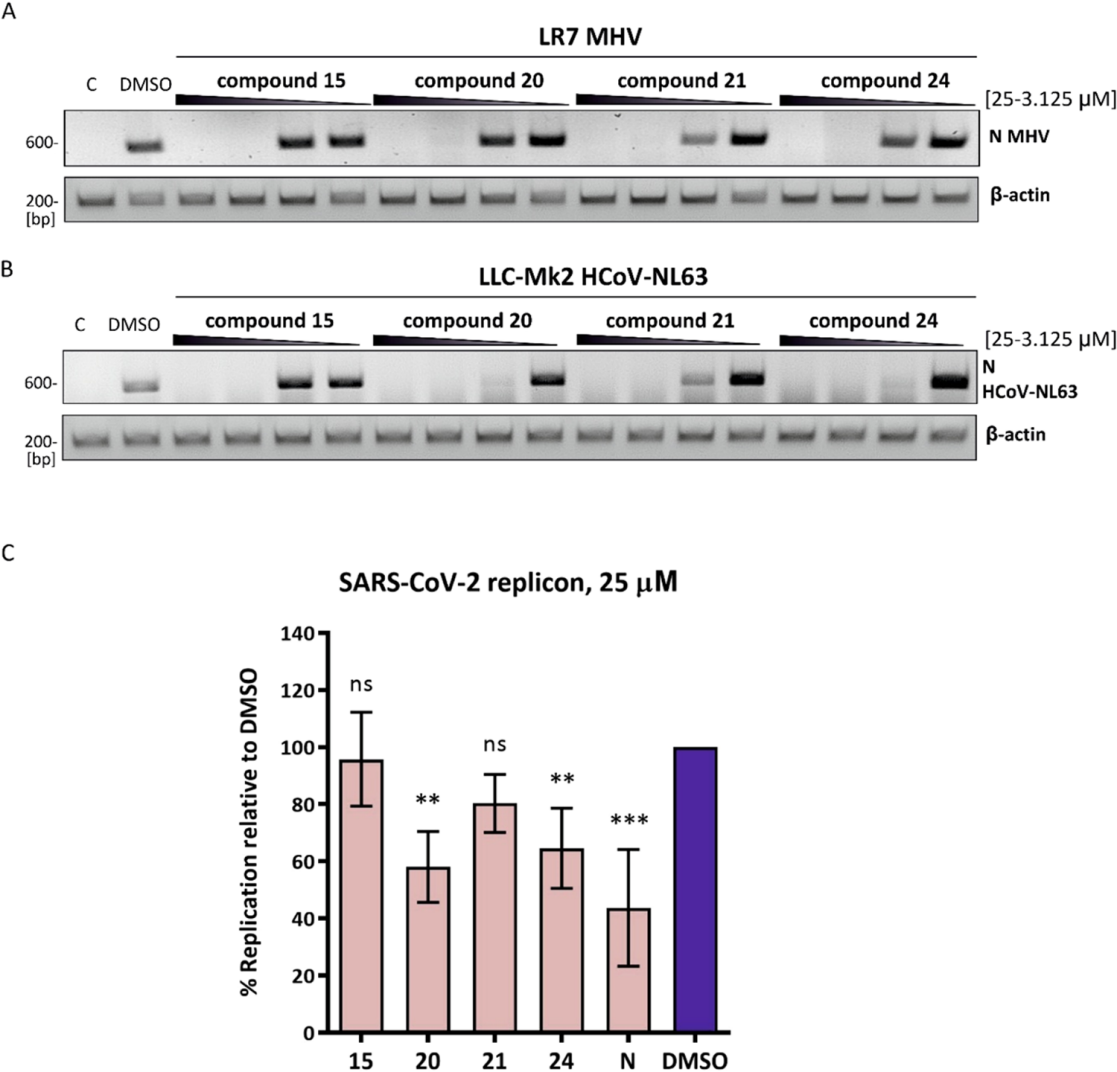
Effect of analyzed glycoconjugates on MHV and HCoV-NL63 RNA synthesis and SARS-CoV-2 replication. LR7 and LLC-Mk2 cells were infected with MHV (MOI 1) (A) and HCoV-NL63 (MOI 0.01) (B), respectively. 1 h p.i. virus-containing medium was removed, and cells were covered with medium supplemented with different concentrations of analyzed compounds (25–3.125 μM). 8 h p.i. (MHV) or 96 h (HCoV-NL63) total RNA was isolated. A fragment of N gene was amplified and analyzed in gel electrophoresis. As a control for RNA isolation, a fragment of a gene encoding β-actin was amplified. C—noninfected cells. Inhibition of SARS-CoV-2 replication. (C) BHK21 cells containing SARS-CoV-2 replicon were seeded and treated with 25 μM of selected compounds, N (Nirmatrelvir) as a reference or an equal volume of diluent DMSO for 48 h. Cells were assayed for luciferase activity using Nano-Glo Luciferase Assay System (Promega). Results were presented as a percentage of luminescence signal from compound-treated cells relative to cells treated with DMSO only. Dunnett’s One-way ANOVA test was used for statistical analysis. The statistical significance of relativity to DMSO is represented as follows: ns *p* > 0.05, ** *p* < 0.01, *** *p* < 0.001. Error bars stand for standard deviation between three biological repeats, three technical replicates each.

Additionally, the SARS-CoV-2 replicon system in the BHK-21 cell line was used to evaluate the effect of the analyzed compounds on viral replication process, in comparison with nirmatrelvir, a known 3C-like protease inhibitor that blocks virus replication.^34^ Treatment of replicon-containing cells with 25 μM compounds **20** and **24**, as well as nirmatrelvir, inhibited SARS-CoV-2 replication (Figure 5C).

Together, these results support the antiviral potential of uridine glycoconjugates and suggest that the inhibition of viral RNA synthesis may be one of their potential mechanisms of action against coronaviruses.

The presented data indicate that the analyzed uridine glycoconjugates (compounds **15**, **20**, **21**, and **24**) exhibit antiviral properties against coronaviruses. While all of the compounds inhibited the replication of model coronaviruses, only compounds **20** and **24** effectively suppressed SARS-CoV-2 replication. Additionally, all tested compounds altered the entry of SARS-CoV-2 VLPs into permissive cells. Therefore, these findings suggest that compounds **20** and **24** may have a dual mechanism of action, involving both the alteration of viral protein glycosylation and the inhibition of RNA replication. However, further studies are needed to confirm and clarify these mechanisms in detail.

## 3 Discussion and Conclusions

Envelope glycoproteins, such as the spike protein in coronaviruses, are essential for the viral life cycle. They play a key role in viral assembly and entry by interacting with host cell receptors and initiating fusion with host cell membranes. Since glycosylation seems to be essential for multiple stages of the coronavirus replication cycle, targeting the glycosylation maturation process represents a promising strategy for the development of anticoronavirus drugs.

In murine hepatitis virus (MHV), for example, tunicamycin treatment disrupts the incorporation of the spike glycoprotein into virions, significantly reducing viral titers.^35^ Similar experiments with various glycosylation inhibitors have confirmed the importance of *N*-glycosylation for the production and infectivity of SARS-CoV-2 virions.^36^ Moreover, studies by Gadlage^37^ suggested that *N*-glycosylation of the MHV-encoded nsp4 protein is crucial for efficient double membrane vesicle (DMV) formation, which is essential for RNA synthesis. Consequently, it is not surprising that inhibition or alterations of the glycosylation process in viral proteins often leads to antiviral effects.^38^

The aim of this study was to evaluate the effect of a series of uridine glycoconjugates as potential glycosyltransferase (GT) inhibitors on the coronavirus life cycle. These compounds were designed to resemble GT donor substrates, incorporating structural modifications such as a 1,2,3-triazole ring and hydrophobic protecting groups, to enhance their stability and antiviral activity. Our previous studies on the *Flaviviridae* viruses highlighted the antiviral potential of similar glycoconjugates, particularly those containing 1,2,3-triazole linkers and TBDMS-protected uridine.^19^ In this study, we found that both the presence of various heteroatoms strongly influenced antiviral activity in the anomeric position of the sugar and the type of bond in the linker structure. Specifically, uridine glycoconjugates with an ether linkage and oxygen as the anomeric heteroatom exhibited anticoronavirus activity, while amide-linked glycoconjugates showed either no activity or high cytotoxicity (Types **II**, **III**, and **V**). Notably, glycoconjugates with sulfur at the anomeric position (Types **IV** and **VI**) were particularly effective, with compound **24** (a d-glucose derivative) displaying the highest potency against both MHV and HCoV-NL63. For most of the glycoconjugate types (except Type **II**), the d-glucose derivative demonstrated slightly higher activity than the d-galactose derivative. However, for glycoconjugates of type **VI**, this difference in activity was notably greater. Considering both the cytotoxicity of the compounds and their antiviral activity against MHV and/or HCoV-NL63, we selected four compounds (**15**, **20**, **21**, and **24)** as the most promising candidates based on preliminary screening. Compound **24** exhibited the lowest IC_50_ values (3.70 μM for MHV and 3.10 μM for HCoV-NL63) along with high selectivity indexes of 26.96 and 19.05 for MHV and HCoV-NL63, respectively. All four selected compounds also demonstrated anti-SARS-CoV-2 activity in Vero cells, though with reduced potency. At a concentration of 25 μM, approximately 60–90% reduction in viral titers was observed, with compound **20** showing the highest level of activity. However, it should be mentioned that some inhibition of the replication cycle during the titration of coronaviruses or SC-VLP internalization may be attributed to the residual amount of compounds in the medium samples.

Using the SARS-CoV-2 VLP system, we confirmed that the selected compounds can interfere with VLP assembly or their interaction with hACE2 receptors in HEK293 cells, as shown by the internalization assay. These results support the hypothesis that the compounds exert glycosyltransferase (GT) inhibitory activity. However, the observed differences in compound activity between the authentic virus and the SC-VLP system may result from variations in the expression of GTs across different cell lines or differences in the glycosylation profile. Additionally, our compounds were designed as analogues of β-1, 4-galactosyltransferase substrate, a family of enzymes localized in the Golgi apparatus.

Recent studies have also suggested that targeting α-glucosidases (e.g., with miglustat, celgosivir) in the early steps in the glycosylation pathway may be more effective at blocking SARS-CoV-2 infection in various cell types than inhibiting downstream Golgi α-mannosidases I and II or α-galactosidases.^36,39,40^ Moreover, additional analysis of coronavirus RNA synthesis in infected cells and SARS-CoV-2 replication using the replicon system suggests that the compounds may also impair viral replication. However, replication inhibition was observed for only two compounds (**20** and **24**), and this effect was partial, suggesting that the compounds may affect replication indirectly.

Since our compounds target glycosyltransferases (GTs), cytotoxicity is a significant concern. While these compounds may interfere with host glycosylation, their impact likely depends on the structure of the specific glycoprotein and the type of host cell. The SARS-CoV-2 S protein is highly glycosylated with certain glycan types and patterns that are preferentially utilized by the virus. Due to its tightly lattice structure, even minor conformational changes in glycan composition may be poorly tolerated, potentially contributing to the greater selectivity of the compounds toward viral proteins. In contrast, many host glycoproteins can tolerate glycosylation inhibition (e.g., glucosidase inhibition) without a significant loss of function, as proper folding does not always rely exclusively on the calnexin/calreticulin system. It has also been demonstrated that the rapid synthesis of viral glycoproteins during acute infection makes enveloped viruses more susceptible to ER α-glucosidase inhibition than host cells.^41^

It should be underlined that some inhibitors targeting enzymes from the glycosidase group, which act at early stages of the glycosylation process, have been tested in clinical trials. Nonetheless, α-glucosidase inhibitors—such as celgosivir (investigated for HCV treatment) and miglustat (NB-DNJ)—have been associated with adverse effects in humans, including diarrhea, weight loss, and flatulence.^42,43^ Inhibitors targeting a broad spectrum of glycosidases may disrupt not only the post-translational modification of host proteins but also carbohydrate metabolism, thereby affecting physiological processes.

Despite these findings, the selectivity of GT inhibitors has remained poorly characterized. Further research is needed to elucidate their specific inhibitory effects on various glycosidases and glycosyltransferases, which could support the development of novel, more selective antiviral agents.

The primary aim of this work was to provide a proof-of-concept demonstration that the tested compounds have the potential to interfere with the replication cycle of coronaviruses. Our results show consistent antiviral effects in relevant *in vitro* models, which supports the hypothesis that interference with host glycosylation pathways may impair viral replication. Given the well-documented role of host glycosylation machinery—particularly in the processing of viral glycoproteins, it is plausible that the observed antiviral effects are at least partially on-target. Therefore, our findings may indicate a potential dual mechanism of action for the tested uridine glycoconjugates: inhibition of GTs and disruption of RNA replication. Further studies are needed to clarify the exact mechanism of action and selectivity toward coronavirus proteins.

## 4 Experimental Section

### 4.1 General Information

The structures and purity of all glycoconjugates were confirmed using NMR and HPLC/MS/MS methods (Supporting Information). All compounds are >95% pure by HPLC analysis. Nuclear magnetic resonance (^1^H NMR and ^13^C NMR) spectra were determined in CDCl_3_, using TMS as an internal standard, and recorded with an Agilent spectrometer at a frequency of 400 MHz or with a Varian spectrometer at a frequency of 600 MHz. NMR solvents were purchased from ACROS Organics (Geel, Belgium) or Deutero GmbH (Kastellaun, Germany). Chemical shifts (δ) are given in parts per million (ppm), and coupling constants (*J*) are given in Hz. Splitting patterns are designated as follows: s, singlet; d, doublet; dd, doublet of doublets; t, triplet; dd ∼ t, doublet of doublets resembling a triplet; ddd, doublet of doublet of doublets; m, multiplet; bs, broad singlet. Optical rotations were measured using a JASCO P-2000 polarimeter equipped with a sodium lamp (589.3 nm) at room temperature. Melting points were determined using an OptiMelt (MPA 100) from Stanford Research Systems. Mass spectra were recorded with a WATERS LCT Premier XE LC/MS system (high-resolution mass spectrometer equipped with an electron spray ionization source and a high-resolution orthogonal TOF analyzer). Reactions were monitored by thin-layer chromatography (TLC) on precoated plates of silica gel 60 F254 (Merck KGaA, Darmstadt, Germany). TLC plates were inspected under UV light (λ = 254 nm) or charring after spraying with 10% ethanolic solution of sulfuric acid. All products were purified using column chromatography performed on Silica Gel 60 (70–230 mesh, Merck KGaA, Darmstadt, Germany) developed with hexane/EtOAc, toluene/EtOAc, or CHCl_3_/MeOH solvent systems in various volume ratios. All evaporations were performed under a diminished pressure at 45 °C on a rotary evaporator.

Sugars (d-glucose and d-galactose), uridine, other chemicals, and solvents were purchased from Sigma-Aldrich (Steinheim, Germany), ACROS Organics (Geel, Belgium) or Avantor (Gliwice, Poland) and were used without purification. Propargyl 2,3,4,6-tetra-*O*-acetyl-β-d-glucopyranoside (**1**), propargyl 2,3,4,6-tetra-*O*-acetyl-β-d-galactopyranoside (**2**),^26^ 2-azidoethyl 2,3,4,6-tetra-*O*-acetyl-β-d-glucopyranoside (**3**),^25^ 2-azidoethyl 2,3,4,6-tetra-*O*-acetyl-β-d-galactopyranoside (**4**),^27^ *N*-(2,3,4,6-tetra-*O*-acetyl-β-d-glucopyranosyl)­azidoacetamide (**5**), *N*-(2,3,4,6-tetra-*O*-acetyl-β-d-galactopyranosyl)­azidoacetamide (**6**),^24^ propargyl 2,3,4,6-tetra-*O*-acetyl-1-thio-β-d-glucopyranoside (**7**), propargyl 2,3,4,6-tetra-*O*-acetyl-1-thio-β-d-galactopyranoside (**8**),^28^ azidomethyl 2,3,4,6-tetra-*O*-acetyl-1-thio-β-d-glucopyranoside (**9**), azidomethyl 2,3,4,6-tetra-*O*-acetyl-1-thio-β-d-galactopyranoside (**10**),^29^ 5′-azido-5′-deoxy-2′,3′-di-*O*-*tert*-butyldimethylsilyl-uridine (**11**),^23^ *N*-propargyl-2′,3′di-*O-tert*-butyldimethylsilyluridine-5′-amide (**12**), 2′,3′di-*O*-*tert*-butyldimethylsilyl-5′-*O*-propargyl-uridine (**13**)^19^ were prepared according to the respective published procedures.

#### 4.1.1 General Procedure for the Synthesis of Glycoconjugates by CuAAC Reaction

To a solutions of a suitably functionalized *O*-acetylated sugar derivatives **1**–**10** (0.2 mmol) and uridine derivatives **11**–**13** (0.2 mmol) in THF (2 mL) and *i*-PrOH (2 mL) mixture, CuSO_4_·5H_2_O (10 mg, 0.04 mmol) dissolved in H_2_O (1 mL) was added immediately after mixing it with sodium ascorbate aqueous solution (16 mg, 0.08 mmol in 1 mL of water). The reaction mixtures thus obtained were stirred for 24–72 h at r.t. The progress of the reaction was monitored on TLC (CHCl_3_:CH_3_OH 20:1 or toluene: ethyl acetate 1:1 solvents system). After completion, the reaction mixtures were concentrated *in vacuo* and purified using column chromatography to give products **14**–**25**.

##### 4.1.1.1 2′,3′-di-O-*tert*-Butyldimethylsilyl-5′-deoxy-5′-[4-(2″,3″,4″,6″-tetra-O-acetyl-β-d-glucopyranosyloxymethyl)-1,2-3-triazol-1-yl]­uridine (**14**)

The crude product was purified by column chromatography (toluene:ethyl acetate gradient from 8:1 to 1:1) to give **14** (162 mg, 92%) as a white solid. [α]_*D*_^20^ = −4.8 (*c* = 1.0, CHCl_3_), *m.p.* = 100–109 °C, ^1^H NMR (CDCl_3_, 600 MHz) δ: 0.02, 0.08, 0.11, 0.12 (4s, 12H, CH_3_Si), 0.88, 0.92 (2s, 18H, (CH_3_)_3_C), 2.01, 2.02, 2.03, 2.09 (4s, 12H, CH_3_CO), 3.74 (ddd, 1H, *J* = 2.4 Hz, *J* = 4.7 Hz, *J* = 10.0 Hz, H-5_glu_), 4.4 (dd ∼ t, 1H, *J* = 4.1 Hz, *J* = 4.1 Hz, H-3′_ur_), 4.19 (dd, 1H, *J* = 2.4 Hz, *J* = 12.3 Hz, H-6a_glu_), 4.26 (dd, 1H, *J* = 4.7 Hz, *J* = 12.3 Hz, H-6b_glu_), 4.32 (m, 1H, H-4′_ur_), 4.57–4.68 (m, 4H, H-2′_ur_, H-1_glu_, H-5′a_ur_, H-5′b_ur_), 4.79 and 4.96 (qAB, 2H, *J* = 12.6 Hz, CH_2_), 5.04 (dd ∼ t, 1H, *J* = 7.9 Hz, *J* = 9.7 Hz, H-2_glu_), 5.11 (dd ∼ t, 1H, *J* = 9.4 Hz, *J* = 10.0 Hz, H-4_glu_), 5.21 (dd ∼ t, 1H, *J* = 9.4 Hz, *J* = 9.7 Hz, H-3_glu_), 5.39 (d, 1H, *J* = 5.3 Hz, H-1′_ur_), 5.72 (dd, 1H, *J* = 2.3 Hz, *J* = 8.2 Hz, H-5_ur_), 6.98 (d, 1H, *J* = 8.2 Hz, H-6_ur_), 7.63 (s, 1H, H-5_triaz_), 8.66 (s, 1H, NH). ^13^C NMR (CDCl_3_, 150 MHz) δ: −4.79, −4.73, −4.57, −4.36 (CH_3_Si), 17.92, 18.02 ((CH_3_)_3_ C), 20.60, 20.68, 20.75, 21.44 (CH_3_)­CO, 25.73, 25.77 ((CH_3_)_3_C), 51.22 (C-5′_ur_), 61.80, 63.34 (C-6_glu_, CH_2_O), 68.32, 71.21, 71.96, 72.35, 72.65, 72.93 (C-3′_ur_, C-2_glu_, C-5_glu_, C-2′_ur_, C-3_glu_, C-4_glu_), 83.06 (C-4′_ur_), 94.97 (C-1′_ur_), 100.24 (C-1_glu_), 102.63 (C-5_ur_), 124.48 (C-5_triaz_), 142.70, 144.28 (C-6_ur_, C-4_triaz_), 149.77 (C-2_ur_), 162.49 (C-4_ur_), 169.39, 170.25, 170.74 (CO). ESI-HRMS: calcd for C_38_H_61_N_5_O_15_Si_2_Na [M + Na]^+^: *m*/*z* 906.3600. Found: *m*/*z* 906.3624.

##### 4.1.1.2 2′,3′-di-O-*tert*-Butyldimethylsilyl-5′-deoxy-5′-[4-(2″,3″,4″,6″-tetra-O-acetyl-β-d-galactopyranosyloxymethyl)-1,2-3-triazol-1-yl]­uridine (**15**)

The crude product was purified by column chromatography (toluene:ethyl acetate gradient from 8:1 to 1:1) to give **15** (135 mg, 77%) as a white solid. [α]_*D*_^20^ = −1.2 (*c* = 1.0, CHCl_3_), *m.p.* = 105–108 °C, ^1^H NMR (CDCl_3_, 400 MHz) δ: 0.02, 0.08, 0.12, 0.13 (4s, 12H, CH_3_Si), 0.88, 0.93 (2s, 18H, (CH_3_)_3_C), 1.99, 2.03, 2.06, 2.16 (4s, 12H, CH_3_CO), 3.95 (dd ∼ t, 1H, *J* = 6.7 Hz, *J* = 6.7 Hz, H-5_gal_), 4.08–4.17 (m, 2H, H-6a_gal_, H-3′_ur_), 4.21 (dd, 1H, *J* = 6.7 Hz, *J* = 11.3 Hz, H-6b_gal_), 4.31 (m, 1H, H-4′_ur_), 4.56–4.69 (m, 4H, H-2′_ur_, H-1_gal_, H-5′a_ur_, H-5′b_ur_), 4.77 and 4.99 (qAB, 2H, *J* = 12.5 Hz, CH_2_), 5.03 (dd, 1H, *J* = 3.1 Hz, *J* = 10.6 Hz, H-3_gal_), 5.24 (dd, 1H, *J* = 7.8 Hz, *J* = 10.6 Hz, H-2_gal_), 5.37 (d, 1H, *J* = 5.5 Hz, H-1′_ur_), 5.40 (d, 1H, *J* = 3.1 Hz, H-4_gal_), 5.72 (dd, 1H, *J* = 2.0 Hz, *J* = 8.0 Hz, H-5_ur_), 6.97 (d, 1H, *J* = 8.0 Hz, H-6_ur_), 7.63 (s, 1H, H-5_triaz_), 8.65 (s, 1H, NH). ^13^C NMR (CDCl_3_, 100 MHz) δ: −4.78, −4.68, −4.52, −4.35 (CH_3_Si), 17.94, 18.05 ((CH_3_)_3_ C), 20.69, 20.83 (CH_3_)­CO, 25.75, 25.80 ((CH_3_)_3_C), 51.20 (C-5′_ur_), 61.26, 63.52 (C-6_gal_, CH_2_O), 67.02, 68.87, 70.77, 72.23, 72.97 (C-3′_ur_, C-2_gal_, C-5_gal_, C-2′_ur_, C-3_gal_, C-4_gal_), 83.18 (C-4′_ur_), 95.09 (C-1′_ur_), 100.90 (C-1_gal_), 102.63 (C-5_ur_), 124.45 (C-5_triaz_), 142.87, 144.38 (C-6_ur_, C-4_triaz_), 149.81 (C-2_ur_), 162.51 (C-4_ur_), 169.78, 170.13, 170.26, 170.46 (CO). ESI-HRMS: calcd for C_38_H_61_N_5_O_15_Si_2_Na [M + Na]^+^: *m*/*z* 906.3600. Found: *m*/*z* 906.3611.

##### 4.1.1.3 *N*-Uracil [(1-(2″,3″,4″,6″-tetra-O-Acetyl-β-d-glucopyranosyloxyethyl)-1,2-3-triazol-4-yl)­methyl]-1′-deoxy-2′,3′-di-O-*tert*-butyldimethylsilyl-β-d-ribofuranuronamide (**16**)

The crude product was purified by column chromatography (first toluene:ethyl acetate gradient from 4:1 to 1:1 and next CHCl_3_:MeOH, gradient from 100:1 to 80:1) to give **16** (112 mg, 60%) as a white solid. [α]_*D*_^20^ = −18.0 (*c* = 1.0, CHCl_3_), *m.p.* = 106–109 °C, ^1^H NMR (CDCl_3_, 400 MHz) δ: −0.03, 0.04, 0.12, 0.18 (4s, 12H, CH_3_Si), 0.85, 0.93 (2s, 18H, (CH_3_)_3_C), 1.98, 2.01, 2.02, 2.09 (4s, 12H, CH_3_CO), 3.70 (ddd, 1H, *J* = 2.4 Hz, *J* = 4.7 Hz, *J* = 9.8 Hz, H-5_glu_), 3.93 (m, 1H, CH), 4.13 (dd, 1H, *J* = 2.4 Hz, *J* = 12.5 Hz, H-6a_glu_), 4.25 (dd, 1H, *J* = 4.7 Hz, *J* = 12.5 Hz, H-6b_glu_), 4.32 (dd, 1H, *J* = 1.2 Hz, *J* = 4.3 Hz, H-3′_ur_), 4.40 (d, 1H, *J* = 1.2 Hz, H-4′_ur_), 4.48 (d, 1H, *J* = 7.8 Hz, H-1_glu_), 4.49–4.67 (m, 5H,4 × CH, H-2′_ur_), 5.00 (dd, 1H, *J* = 7.8 Hz, *J* = 9.8 Hz, H-2_glu_), 5.06 (dd ∼ t, 1H, *J* = 9.4 Hz, *J* = 9.8 Hz, H-4_glu_), 5.19 (dd ∼ t, 1H, *J* = 9.4 Hz, *J* = 9.8 Hz, H-3_glu_), 5.51 (d, 1H, *J* = 7.4 Hz, H-1′_ur_), 5.78 (dd, 1H, *J* = 2.2 Hz, *J* = 8.0 Hz, H-5_ur_), 7.55 (d, 1H, *J* = 8.0 Hz, H-6_ur_), 7.58 (s, 1H, H-5_triaz_), 7.94 (dd ∼ t, 1H, *J* = 5.5 Hz, *J* = 5.5 Hz, NHCH_2_), 8.58 (m, 1H, NH). ^13^C NMR (CDCl_3_, 100 MHz) δ: −5.11, −4.69, −4.49 (CH_3_Si), 17.89, 18.04 ((CH_3_)_3_ C), 20.58, 20.74 (CH_3_)­CO, 25.69, 25.80 ((CH_3_)_3_C), 34.89 (CH_2_), 50.09 (CH_2_N), 61.76, 67.74 (C-6_glu_, CH_2_O), 68.27, 71.07, 71.22, 72.04, 72.35, 74.80 (C-3′_ur_, C-2_glu_, C-5_glu_, C-2′_ur_, C-3_glu_, C-4_glu_), 85.34 (C-4′_ur_), 94.07 (C-1′_ur_), 100.52 (C-1_glu_), 102.91 (C-5_ur_), 123.18 (C-5_triaz_), 143.85, 144.16 (C-6_ur_, C-4_triaz_), 150.42 (C-2_ur_), 162.30 (C-4_ur_), 169.24, 169.38, 169.86, 170.13, 170.65 (CO). ESI-HRMS: calcd for C_40_H_64_N_6_O_16_Si_2_Na [M + Na]^+^: *m*/*z* 963.3815. Found: *m*/*z* 963.3805.

##### 4.1.1.4 *N*-Uracil [(1-(2″, 3″, 4″, 6″-tetra-O-Acetyl-β-d-galactopyranosyloxyethyl)-1,2-3-triazol-4-yl)­methyl]-1′-deoxy-2′,3′-di-O-*tert*-butyldimethylsilyl-β-d-ribofuranuronamide (**17**)

The crude product was purified by column chromatography (first toluene:ethyl acetate gradient from 4:1 to 1:1 and next CHCl_3_:MeOH, gradient from 100:1 to 80:1) to give **17** (153 mg, 81%) as a white solid. [α]_*D*_^20^ = −17.6 (*c* = 1.0, CHCl_3_), *m.p.* = 116–120 °C, ^1^H NMR (CDCl_3_, 400 MHz) δ: 0.03, 0.04, 0.12, 0.17 (4s, 12H, CH_3_Si), 0.84, 0.93 (2s, 18H, (CH_3_)_3_C), 1.99, 2.05, 2.06, 2.17 (4s, 12H, CH_3_CO), 3.89–3.96 (m, 2H, CH, H-5_gal_), 4.11 (dd, 1H, *J* = 6.7 Hz, *J* = 11.3 Hz, H-6a_gal_), 4.17 (dd, 1H, *J* = 6.7 Hz, *J* = 11.3 Hz, H-6b_gal_), 4.25 (m, 1H, CH), 4.32 (dd, 1H, *J* = 1.6 Hz, *J* = 4.3 Hz, H-3′_ur_), 4.41 (d, 1H, *J* = 1.6 Hz, H-4′_ur_), 4.45 (d, 1H, *J* = 7.8 Hz, H-1_gal_), 4.46–4.67 (m, 5H, 5 × CH, H-2′_ur_)_,_ 5.00 (dd, 1H, *J* = 3.5 Hz, *J* = 10.2 Hz, H-3_gal_), 5.18 (dd, 1H, *J* = 7.8 Hz, *J* = 10.2 Hz, H-2_gal_), 5.40 (dd, 1H, *J* = 0.8 Hz, *J* = 3.5 Hz, H-4_gal_), 5.54 (d, 1H, *J* = 7.4 Hz, H-1′_ur_), 5.79 (dd, 1H, *J* = 2.4 Hz, *J* = 8.2 Hz, H-5_ur_), 7.59 (d, 1H, *J* = 8.2 Hz, H-6_ur_), 7.59 (s, 1H, H-5_triaz_), 7.88 (dd ∼ t, 1H, *J* = 5.5 Hz, *J* = 5.5 Hz, NHCH_2_), 8.81 (s, 1H, NH). ^13^C NMR (CDCl_3_, 100 MHz) δ: −5.09, −4.71, −4.68, −4.50 (CH_3_Si), 17.89, 18.04 ((CH_3_)_3_ C), 20.56, 20.64, 20.66, 20.68 (CH_3_)­CO, 25.70, 25.80 ((CH_3_)_3_C), 34.94 (CH_2_), 50.10 (CH_2_N), 61.17, 66.87 (C-6_gal_, CH_2_O), 67.59, 68.68, 70.52, 70.97, 71.39, 74.79 (C-3′_ur_, C-2_gal_, C-5_gal_, C-2′_ur_, C-3_gal_, C-4_gal_), 85.26 (C-4′_ur_), 93.83 (C-1′_ur_), 100.95 (C-1_gal_), 102.90 (C-5_ur_), 123.26 (C-5_triaz_), 143.75, 144.07 (C-6_ur_, C-4_triaz_), 150.49 (C-2_ur_), 162.50 (C-4_ur_), 169.28, 170.02, 170.07, 170.13, 170.40 (CO). ESI-HRMS: calcd for C_40_H_64_N_6_O_16_Si_2_Na [M + Na]^+^: *m*/*z* 963.3815. Found: *m*/*z* 963.3811.

##### 4.1.1.5 *N*-Uracil [((1-(2-oxo-(2″,3″,4″,6″-tetra-O-Acetyl-β-d-glucopyranosyl)­amino)­ethyl)-1,2-3-triazol-4-yl)­methyl]-1′-deoxy-2′,3′-di-O-*tert*-butyldimethylsilyl-β-d-ribofuranuronamide (**18**)

The crude product was purified by column chromatography (CHCl_3_:MeOH, gradient from 100:1 to 80:1) to give **18** (126 mg, 66%) as a white solid. [α]_*D*_^20^ = −12.0 (*c* = 1.0, CHCl_3_), *m.p.* = 141–143 °C, ^1^H NMR (CDCl_3_, 400 MHz) δ: −0.04, 0.05, 0.13, 0.20 (4s, 12H, CH_3_Si), 0.85, 0.94 (2s, 18H, (CH_3_)_3_C), 2.00, 2.01, 2.03, 2.07 (4s, 12H, CH_3_CO), 4.01 (ddd, 1H, *J* = 1.6 Hz, *J* = 4.1 Hz, *J* = 10.28 Hz, H-5_glu_), 4.07 (dd, 1H, *J* = 1.6 Hz, *J* = 12.5 Hz, H-6a_glu_), 4.31 (dd, 1H, *J* = 4.1 Hz, *J* = 12.5 Hz, H-6b_glu_), 4.35 (d, 1H, *J* = 4.7 Hz, H-3′_ur_), 4.39 (bs, 1H, H-4′_ur_), 4.53–4.68 (m, 2H, CH_2_), 4.87 (dd, 1H, *J* = 4.7 Hz, *J* = 8.0 Hz, H-2′_ur_), 4.91 (dd ∼ t, 1H, *J* = 9.4 Hz, *J* = 10.2 Hz, H-4_glu_), 4.98 and 5.18 (qAB, 2H, *J* = 7.8 Hz, CH_2_), 5.08 (dd ∼ t, 1H, *J* = 9.4 Hz, *J* = 9.8 Hz, H-2_glu_), 5.27–5.38 (m, 3H, H-1′_ur_, H-1_glu_, H-3_glu_), 5.75 (d, 1H, *J* = 8.2 Hz, H-5_ur_), 7.08 (d, 1H, *J* = 8.6 Hz, NH), 7.31 (d, 1H, *J* = 8.2 Hz, H-6_ur_), 7.86 (m, 1H, NH), 7.92 (s, 1H, H-5_triaz_), 9.17 (s, 1H, NH). ^13^C NMR (CDCl_3_, 100 MHz) δ: −5.06, −4.69, −4.67, −4.45 (CH_3_Si), 17.88, 18.04 ((CH_3_)_3_ C), 20.56, 20.58, 20.62, 20.74 (CH_3_)­CO, 25.71, 25.80 ((CH_3_)_3_C), 35.46 (CH_2_NH), 52.48 (NCH_2_CO), 61.50 (C-6_glu_), 67.90, 70.21, 70.67, 72.44, 73.67, 74.82 (C-3′_ur_, C-2_glu_, C-5_glu_, C-2′_ur_, C-3_glu_, C-4_glu_), 78.55 (C-1_glu_), 85.76 (C-4′_ur_), 96.07 (C-1′_ur_), 102.90 (C-5_ur_), 124.58 (C-5_triaz_), 144.29, 144.73 (C-6_ur_, C-4_triaz_), 150.48 (C-2_ur_), 162.60 (C-4_ur_), 166.04, 169.14 (CONH), 169.58, 169.74, 170.55, 171.46 (CO). ESI-HRMS: calcd for C_40_H_63_N_7_O_16_Si_2_Na [M + Na]^+^: *m*/*z* 976.3768. Found: *m*/*z* 976.3769.

##### 4.1.1.6 *N*-Uracil [((1-(2-oxo-(2″,3″,4″,6″-tetra-O-Acetyl-β-d-galactopyranosyl)­amino)­ethyl)-1,2-3-triazol-4-yl)­methyl]-1′-deoxy-2′,3′-di-O-*tert*-butyldimethylsilyl-β-d-ribofuranuronamide (**19**)

The crude product was purified by column chromatography (CHCl_3_:MeOH, gradient from 100:1 to 60:1) to give **19** (130 mg, 68%) as a white solid. [α]_*D*_^20^ = −3.8 (*c* = 1.0, CHCl_3_), *m.p.* = 135–137 °C, ^1^H NMR (CDCl_3_, 400 MHz) δ: −0.04, 0.05, 0.13, 0.19 (4s, 12H, CH_3_Si), 0.85, 0.94 (2s, 18H, (CH_3_)_3_C), 1.98, 2.01, 2.03, 2.14 (4s, 12H, CH_3_CO), 4.04–4.21 (m, 3H, H-5_gal_, H-6a_gal_, H-6b_gal_), 4.34 (d, 1H, *J* = 4.7 Hz, H-3′_ur_), 4.39 (s, 1H, H-4′_ur_), 4.54–4.68 (m, 2H, CH_2_), 4.84 (dd, 1H, *J* = 4.7 Hz, *J* = 7.6 Hz, H-2′_ur_), 4.98 and 5.17 (qAB, 2H, *J* = 16.0 Hz, CH_2_), 5.07 (1H, *J* = 9.4 Hz, *J* = 10.2 Hz, H-2_gal_), 5.18 (dd, 1H, *J* = 3.5 Hz, *J* = 10.2 Hz, H-3_gal_), 5.30 (dd ∼ t, 1H, *J* = 9.0 Hz, *J* = 9.0 Hz, H-1_gal_), 5.32 (d, 1H, *J* = 7.6 Hz, H-1′_ur_), 5.45 (d, 1H, *J* = 3.5 Hz, H-4_gal_), 5.75 (d, 1H, *J* = 8.2 Hz, H-5_ur_), 7.01 (d, 1H, *J* = 8.6 Hz, NH), 7.34 (d, 1H, *J* = 8.2 Hz, H-6_ur_), 7.87 (m, 1H, NH), 7.90 (s, 1H, H-5_triaz_), 9.17 (s, 1H, NH). ^13^C NMR (CDCl_3_, 100 MHz) δ: −5.05, −4.69, −4.45 (CH_3_Si), 17.88, 18.04 ((CH_3_)_3_ C), 20.52, 20.60, 20.68 (CH_3_)­CO, 25.71, 25.80 ((CH_3_)_3_C), 35.40 (CH_2_NH), 52.54 (NCH_2_CO), 60.99 (C-6_gal_), 67.16, 68.37, 70.35, 70.51, 72.53, 74.84 (C-3′_ur_, C-2_gal_, C-5_gal_, C-2′_ur_, C-3_gal_, C-4_gal_), 78.80 (C-1_gal_), 85.69 (C-4′_ur_), 95.84 (C-1′_ur_), 102.93 (C-5_ur_), 124.44 (C-5_triaz_), 144.41, 144.60 (C-6_ur_, C-4_triaz_), 150.50 (C-2_ur_), 162.60 (C-4_ur_), 165.88, 169.18 (CONH), 169.61, 169.96, 170.31, 171.70 (CO). ESI-HRMS: calcd for C_40_H_63_N_7_O_16_Si_2_Na [M + Na]^+^: *m*/*z* 976.3768. Found: *m*/*z* 976.3773.

##### 4.1.1.7 2′,3′-di-O-*tert*-Butyldimethylsilyl-5′-deoxy-5′-[4-(2″,3″,4″,6″-tetra-O-acetyl-β-d-glucopyranosylthiomethyl)-1,2-3-triazol-1-yl]­uridine (**20**)

The crude product was purified by column chromatography (first toluene:ethyl acetate gradient from 4:1 to 1:1 and next CHCl_3_:MeOH, gradient from 100:1 to 80:1) to give **20** (143 mg, 80%) as a white solid. [α]_*D*_^20^ = −20.0 (*c* = 0.5, CHCl_3_), *m.p.* = 103–107 °C, ^1^H NMR (CDCl_3_, 400 MHz) δ: 0.02, 0.07, 0.11 (3s, 12H, CH_3_Si), 0.87, 0.92 (2s, 18H, (CH_3_)_3_C), 2.00, 2.02, 2.03, 2.08 (4s, 12H, CH_3_CO), 3.72 (ddd, 1H, *J* = 2.7 Hz, *J* = 4.7 Hz, *J* = 10.2 Hz, H-5_glu_), 3.90 and 4.09 (qAB, 2H, *J* = 14.3 Hz, CH_2_), 4.12–4.19 (m, 2H, H-6a_glu_, H-3′_ur_), 4.22 (dd, 1H, *J* = 4.7 Hz, *J* = 12.2 Hz, H-6b_glu_), 4.31 (m, 1H, H-4′_ur_), 4.42 (dd ∼ t, 1H, *J* = 4.7 Hz, *J* = 5.5 Hz, H-2′_ur_), 4.59 (dd, 1H, *J* = 6.7 Hz, *J* = 14.2 Hz, H-5′a_ur_), 4.61 (d, 1H, *J* = 9.8 Hz, H-1_glu_), 4.67 (dd, 1H, *J* = 4.2 Hz, *J* = 14.2 Hz, H-5′b_ur_), 5.05 (dd ∼ t, 1H, *J* = 9.4 Hz, *J* = 10.2 Hz, H-4_glu_), 5.10 (dd ∼ t, 1H, *J* = 9.4 Hz, *J* = 9.8 Hz, H-2_glu_), 5.21 (dd ∼ t, 1H, *J* = 9.4 Hz, *J* = 9.4 Hz, H-3_glu_), 5.47 (d, 1H, *J* = 5.5 Hz, H-1′_ur_), 5.73 (dd, 1H, *J* = 2.0 Hz, *J* = 8.2 Hz, H-5_ur_), 6.95 (d, 1H, *J* = 8.2 Hz, H-6_ur_), 7.61 (s, 1H, H-5_triaz_), 8.72 (s, 1H, NH). ^13^C NMR (CDCl_3_, 100 MHz) δ: −4.71, −4.68, −4.55, −4.31 (CH_3_Si), 17.94, 18.03 ((CH_3_)_3_ C), 20.59, 20.61, 20.68, 20.79 (CH_3_)­CO, 25.74, 25.80 ((CH_3_)_3_C), 30.93 (CH_2_S), 51.35 (C-5′_ur_), 61.89, 68.26, 69.88, 72.73, 72.80, 73.79, 75.93 (C-6_glu_, C-3′_ur_, C-2_glu_, C-5_glu_, C-2′_ur_, C-3_glu_, C-4_glu_), 82.87, 82.93 (C-4′_ur_, C-1_glu_), 94.02 (C-1′_ur_), 102.67 (C-5_ur_), 123.95 (C-5_triaz_), 142.22, 144.70 (C-6_ur_, C-4_triaz_), 149.84 (C-2_ur_), 162.62 (C-4_ur_), 169.42, 169.47, 170.16, 170.72 (CO). ESI-HRMS: calcd for C_38_H_62_N_5_O_14_SSi_2_ [M + H]^+^: *m*/*z* 900.3553. Found: *m*/*z* 900.3585.

##### 4.1.1.8 2′,3′-di-O-*tert*-Butyldimethylsilyl-5′-deoxy-5′-[4-(2″,3″,4″,6″-tetra-O-acetyl-β-d-galactopyranosylthiomethyl)-1,2-3-triazol-1-yl]­uridine (**21**)

The crude product was purified by column chromatography (toluene:ethyl acetate gradient from 2:1 to 1:1) to give **21** (100 mg, 56%) as a white solid. [α]_*D*_^20^ = −14.2 (*c* = 1, CHCl_3_), *m.p.* = 98–102 °C, ^1^H NMR (CDCl_3_, 400 MHz) δ: −0.01, 0.01, 0.07, 0.11 (4s, 12H, CH_3_Si), 0.87, 0.92 (2s, 18H, (CH_3_)_3_C), 1.98, 2.04, 2.05, 2.15 (4s, 12H, CH_3_CO), 3.88–4.01 (m, 2H, CH, H-5_gal_), 4.04–4.20 (m, 4H, H-6a_gal_, H-6b_gal_, H-3′_ur_, CH), 4.31 (m, 1H, H-4′_ur_), 4.41 (dd ∼ t, 1H, *J* = 4.7 Hz, *J* = 5.1 Hz, H-2′_ur_), 4.53–4.71 (m, 3H, H-5′a_ur_, H-5′b_ur_, H-1_gal_), 5.04 (dd, 1H, *J* = 3.1 Hz, *J* = 9.8 Hz, H-3_gal_), 5.25 (dd ∼ t, 1H, *J* = 9.8 Hz, *J* = 9.8 Hz, H-2_gal_), 5.43 (d, 1H, *J* = 3.1 Hz, H-4_gal_), 5.47 (d, 1H, *J* = 5.1 Hz, H-1′_ur_), 5.74 (dd, 1H, *J* = 2.0 Hz, *J* = 8.2 Hz, H-5_ur_), 6.95 (d, 1H, *J* = 8.2 Hz, H-6_ur_), 7.60 (s, 1H, H-5_triaz_), 8.50 (s, 1H, NH). ^13^C NMR (CDCl_3_, 100 MHz) δ: −4.73, −4.67, −4.54, −4.31 (CH_3_Si), 17.94, 18.03 ((CH_3_)_3_ C), 20.58, 20.68, 20.72, 20.79 (CH_3_)­CO, 25.73, 25.79 ((CH_3_)_3_C), 24.29 (CH_2_S), 51.35 (C-5′_ur_), 61.32, 67.27, 71.77, 72.64, 72.77, 74.58 (C-6_gal_, C-3′_ur_, C-2_gal_, C-5_gal_, C-2′_ur_, C-3_gal_, C-4_gal_), 82.98, 83.40 (C-4′_ur_, C-1_gal_), 93.98 (C-1′_ur_), 102.68 (C-5_ur_), 125.29 (C-5_triaz_), 137.87, 142.25 (C-6_ur_, C-4_triaz_), 149.77 (C-2_ur_), 162.452 (C-4_ur_), 169.69, 170.03, 170.22, 170.48 (CO). ESI-HRMS: calcd for C_38_H_62_N_5_O_14_SSi_2_ [M + H]^+^: *m*/*z* 900.3553. Found: *m*/*z* 900.3560.

##### 4.1.1.9 *N*-Uracil [(1-(2″,3″,4″,6″-tetra-O-Acetyl-β-d-glucopyranosylthiomethyl)-1,2-3-triazol-4-yl)­methyl]-1′-deoxy-2′,3′-di-O-*tert*-butyldimethylsilyl-β-d-ribofuranuronamide (**22**)

The crude product was purified by column chromatography (toluene:ethyl acetate gradient from 2:1 to 1:1) to give **22** (169 mg, 91%) as a white solid. [α]_*D*_^20^ = −34.6 (*c* = 1, CHCl_3_), *m.p.* = 108–112 °C, ^1^H NMR (CDCl_3_, 400 MHz) δ: −0.11, 0.05, 0.12, 0.17 (4s, 12H, CH_3_Si), 0.85, 0.93 (2s, 18H, (CH_3_)_3_C), 2.00, 2.01, 2.02, 2.10 (4s, 12H, CH_3_CO), 3.71 (ddd, 1H, *J* = 2.7 Hz, *J* = 4.3 Hz, *J* = 10.2 Hz, H-5_glu_), 4.09–4.19 (m, 2H, H-6a_glu_, H-6b_glu_), 4.29 (dd, 1H, *J* = 1.6 Hz, *J* = 4.3 Hz, H-3′_ur_), 4.39 (d, 1H, *J* = 1.6 Hz, H-4′_ur_), 4.52–4.68 (m, 4H,CH_2_, H-1_glu_, H-2′_ur_), 5.03 (dd ∼ t, 1H, *J* = 9.4 Hz, *J* = 10.2 Hz, H-4_glu_), 5.09 (dd ∼ t, 1H, *J* = 9.0 Hz, *J* = 9.4 Hz, H-2_glu_), 5.20 (dd ∼ t, 1H, *J* = 9.4 Hz, *J* = 9.4 Hz, H-3_glu_), 5.37 and 5.64 (qAB, 2H, *J* = 14.5 Hz, CH_2_), 5.48 (d, 1H, *J* = 7.0 Hz, H-1′_ur_), 5.78 (dd, 1H, *J* = 2.3 Hz, *J* = 8.2 Hz, H-5_ur_), 7.58 (d, 1H, *J* = 8.2 Hz, H-6_ur_), 7.76 (s, 1H, H-5_triaz_), 7.98 (dd ∼ t, 1H, *J* = 5.5 Hz, *J* = 5.9 Hz, NH), 8.73 (bs, 1H, NH). ^13^C NMR (CDCl_3_, 100 MHz) δ: −5.02, −4.73, −4.67, −4.53 (CH_3_Si), 17.89, 18.03 ((CH_3_)_3_ C), 20.55, 20.59, 20.81 (CH_3_)­CO, 25.70, 25.78 ((CH_3_)_3_C), 34.91 (CH_2_S), 48.50 (CH_2_NH), 61.64 (C-6_glu_), 67.96, 69.71, 71.33, 73.52, 74.79 (C-3′_ur_, C-2_glu_, C-5_glu_, C-2′_ur_, C-3_glu_, C-4_glu_), 82.26 _(_C-1_glu_), 85.12 (C-4′_ur_), 94.36 (C-1′_ur_), 102.80 (C-5_ur_), 121.97 (C-5_triaz_), 143.92, 145.30 (C-6_ur_, C-4_triaz_), 150.38 (C-2_ur_), 162.55 (C-4_ur_), 169.35, 169.48, 170.00, 170.76 (CO). ESI-HRMS: calcd for C_39_H_62_N_6_O_15_SSi_2_Na [M + Na]^+^: *m*/*z* 965.3430. Found: *m*/*z* 965.3432.

##### 4.1.1.10 *N*-Uracil [(1-(2″,3″,4″,6″-tetra-O-Acetyl-β-d-galactopyranosylthiomethyl)-1,2-3-triazol-4-yl)­methyl]-1′-deoxy-2′,3′-di-O-*tert*-butyldimethylsilyl-β-d-ribofuranuronamide (**23**)

The crude product was purified by column chromatography (toluene:ethyl acetate gradient from 2:1 to 1:1) to give **23** (94 mg, 50%) as a white solid. [α]_*D*_^20^ = −29.0 (*c* = 1, CHCl_3_), *m.p.* = 118–123 °C, ^1^H NMR (CDCl_3_, 600 MHz) δ: −0.02, 0.05, 0.13, 0.18 (4s, 12H, CH_3_Si), 0.85, 0.93 (2s, 18H, (CH_3_)_3_C), 2.03, 2.06, 2.07, 2.17 (4s, 12H, CH_3_CO), 3.94 (ddd, 1H, *J* = 0.8 Hz, *J* = 5.5 Hz, *J* = 5.9 Hz, H-5_gal_), 4.01–4.09 (m, 2H, H-6a_gal_, H-6b_gal_), 4.29 (dd, 1H, *J* = 1.6 Hz, *J* = 4.3 Hz, H-3′_ur_), 4.39 (d, 1H, *J* = 1.6 Hz, H-4′_ur_), 4.52–4.69 (m, 4H,CH_2_, H-1_gal_, H-2′_ur_), 5.03 (dd, 1H, *J* = 3.5 Hz, *J* = 9.8 Hz, H-3_gal_), 5.23 (dd ∼ t, 1H, *J* = 9.8 Hz, *J* = 10.2 Hz, H-2_gal_), 5.39 and 5.65 (qAB, 2H, *J* = 14.5 Hz, CH_2_), 5.42 (dd ∼ t, 1H, *J* = 0.8 Hz, *J* = 3.5 Hz, H-4_gal_), 5.45 (d, 1H, *J* = 7.0 Hz, H-1′_ur_), 5.78 (dd, 1H, *J* = 2.0 Hz, *J* = 8.2 Hz, H-5_ur_), 7.54 (d, 1H, *J* = 8.2 Hz, H-6_ur_), 7.73 (s, 1H, H-5_triaz_), 8.03 (dd ∼ t, 1H, *J* = 5.5 Hz, *J* = 5.9 Hz, NH), 8.57 (bs, 1H, NH). ^13^C NMR (CDCl_3_, 150 MHz) δ: −5.05, −4.72, −4.67, −4.50 (CH_3_Si), 17.89, 18.03 ((CH_3_)_3_ C), 20.52, 20.64, 20.70, 20.73 (CH_3_)­CO, 25.70, 25.78 ((CH_3_)_3_C), 34.92 (CH_2_S), 48.49 (CH_2_NH), 61.17 (C-6_gal_), 67.01, 67.07, 71.13, 71.51, 74.83, 74.91 (C-3′_ur_, C-2_gal_, C-5_gal_, C-2′_ur_, C-3_gal_, C-4_gal_), 82.76 (C-1_gal_), 85.20 (C-4′_ur_), 94.56 (C-1′_ur_), 102.84 (C-5_ur_), 121.88 (C-5_triaz_), 144.00, 145.33 (C-6_ur_, C-4_triaz_), 150.37 (C-2_ur_), 162.33 (C-4_ur_), 169.50, 169.74, 169.82, 170.13 (CO). ESI-HRMS: calcd for C_39_H_62_N_6_O_15_SSi_2_Na [M + Na]^+^: *m*/*z* 965.3430. Found: *m*/*z* 965.3433.

##### 4.1.1.11 2′,3′-di-O-*tert*-Butyldimethylsilyl-5′-O-[(1-(2″,3″,4″,6″-tetra-O-acetyl-β-d-glucopyranosylthiomethyl)-1,2-3-triazol-4-yl)­methyl]­uridine (**24**)

The crude product was purified by column chromatography (toluene:ethyl acetate gradient from 4:1 to 2:1) to give **24** (156 mg, 85%) as a white solid. [α]_*D*_^20^ = −14.2 (*c* = 1, CHCl_3_), *m.p.* = 91–93 °C, ^1^H NMR (CDCl_3_, 600 MHz) δ: 0.06, 0.07, 0.10 (3s, 12H, CH_3_Si), 0.89, 0.90 (2s, 18H, (CH_3_)_3_C), 2.00, 2.01, 2.03, 2.09 (4s, 12H, CH_3_CO), 3.71 (dd, 1H, *J* = 2.0 Hz, *J* = 10.9 Hz, H-5′a_ur_), 3.75 (ddd, 1H, *J* = 2.3 Hz, *J* = 4.4 Hz, *J* = 10.0 Hz, H-5_glu_),3.91 (dd, 1H, *J* = 2.4 Hz, *J* = 10.9 Hz, H-5′b_ur_), 4.07–4.16 (m, 4H, H-6a_glu_, H-2′_ur_, H-3′_ur_, H-4′_ur_), 4.21 (dd, 1H, *J* = 4.4 Hz, *J* = 12.6 Hz, H-6b_glu_), 4.65 (d, 1H, *J* = 10.0 Hz, H-1_glu_), 4.66 and 4.72 (qAB, 2H, *J* = 12.0 Hz, CH_2_), 5.08 (dd ∼ t, 1H, *J* = 9.4 Hz, *J* = 10.0 Hz, H-4_glu_), 5.12 (dd ∼ t, 1H, *J* = 9.4 Hz, *J* = 10.0 Hz, H-2_glu_), 5.21 (dd ∼ t, 1H, *J* = 9.4 Hz, *J* = 9.4 Hz, H-3_glu_), 5.34 and 5.73 (qAB, 2H, *J* = 14.7 Hz, CH_2_), 5.64 (dd, 1H, *J* = 2.6 Hz, *J* = 8.2 Hz, H-5_ur_), 5.74 (d, 1H, *J* = 2.9 Hz, H-1′_ur_), 7.72 (s, 1H, H-5_triaz_), 7.99 (d, 1H, *J* = 8.2 Hz, H-6_ur_), 8.04 (bs, 1H, NH). ^13^C NMR (CDCl_3_, 150 MHz) δ: −5.01, −4.83, –4.58, −4.30 (CH_3_Si), 18.01, 18.09 ((CH_3_)_3_ C), 20.56, 20.78 (CH_3_)­CO, 25.79, 25.83 ((CH_3_)_3_C), 48.27 (CH_2_N), 61.60, 64.36 (CH_2_O, C-6_glu_), 67.94, 68.43, 69.73, 70.63, 73.48, 75.83 (C-3′_ur_, C-2_glu_, C-5_glu_, C-2′_ur_, C-3_glu_, C-4_glu_), 81.97, 82.59 (C-1_glu_, C-4′_ur_), 89.76 (C-1′_ur_), 101.72 (C-5_ur_), 122.18 (C-5_triaz_), 140.74, 144.99 (C-6_ur_, C-4_triaz_), 150.05 (C-2_ur_), 162.85 (C-4_ur_), 169.39, 170.00, 170.49 (CO). ESI-HRMS: calcd for C_39_H_63_N_5_O_15_SSi_2_Na [M + Na]^+^: *m*/*z* 952.3478. Found: *m*/*z* 952.3474.

##### 4.1.1.12 2′,3′-di-O-*tert*-Butyldimethylsilyl-5′-O-[(1-(2″,3″,4″,6″-tetra-O-acetyl-β-d-galactopyranosylthiomethyl)-1,2-3-triazol-4-yl)­methyl]­uridine (**25**)

The crude product was purified by column chromatography (toluene:ethyl acetate gradient from 4:1 to 2:1) to give **25** (82 mg, 45%) as a white solid. [α]_*D*_^20^ = −10.2 (*c* = 1, CHCl_3_), *m.p.* = 104–110 °C, ^1^H NMR (CDCl_3_, 400 MHz) δ: 0.06, 0.08, 0.11, 0.12 (4s, 12H, CH_3_Si), 0.89, 0.90 (2s, 18H, (CH_3_)_3_C), 1.98, 2.02, 2.05, 2.18 (4s, 12H, CH_3_CO), 3.72 (dd, 1H, *J* = 2.0 Hz, *J* = 10.6 Hz, H-5′a_ur_), 3.93–4.18 (m, 6H, H-5_gal_, H-6a_gal_, H-6b_gal_, H-2′_ur_, H-3′_ur_, H-4′_ur_), 4.65 (d, 1H, *J* = 9.8 Hz, H-1_gal_), 4.66 and 4.72 (qAB, 2H, *J* = 11.9 Hz, CH_2_), 5.06 (dd, 1H, *J* = 3.1 Hz, *J* = 10.2 Hz, H-3_gal_), 5.29 (dd ∼ t, 1H, *J* = 9.8 Hz, *J* = 10.2 Hz, H-2_gal_), 5.35 and 5.77 (qAB, 2H, *J* = 14.6 Hz, CH_2_), 5.44 (dd, 1H, *J* = 0.9 Hz, *J* = 3.1 Hz, H-4_gal_), 5.62 (dd, 1H, *J* = 2.3 Hz, *J* = 8.2 Hz, H-5_ur_), 5.72 (d, 1H, *J* = 2.7 Hz, H-1′_ur_), 7.72 (s, 1H, H-5_triaz_), 8.01 (d, 1H, *J* = 8.2 Hz, H-6_ur_), 8.31 (bs, 1H, NH). ^13^C NMR (CDCl_3_, 100 MHz) δ: −4.92, −4.73, −4.43, −4.17 (CH_3_Si), 148.14, 18.22 ((CH_3_)_3_ C), 20.67, 20.77, 20.80, 20.81 (CH_3_)­CO, 25.93, 25.96 ((CH_3_)_3_C), 48.33 (CH_2_N), 61.10, 64.55 (CH_2_O, C-6_gal_), 67.09, 67.24, 68.53, 70.63, 71.59, 75.07, 75.95 (C-5′_ur_, C-3′_ur_, C-2_gal_, C-5_gal_, C-2′_ur_, C-3_gal_, C-4_gal_), 82.52, 82.63 (C-4′_ur_, C-1_gal_), 90.02 (C-1′_ur_), 101.76 (C-5_ur_), 122.22 (C-5_triaz_), 140.89, 145.01 (C-6_ur_, C-4_triaz_), 150.23 (C-2_ur_), 163.18 (C-4_ur_), 169.76, 169.95, 170.23, 170.41 (CO). ESI-HRMS: calcd for C_39_H_63_N_5_O_15_SSi_2_Na [M + Na]^+^: *m*/*z* 952.3478. Found: *m*/*z* 952.3477.

### 4.2 Analyzed Compounds

The 10 mM stock solutions of analyzed compounds and nirmatrelvir (Molport, SIA, Latvia) were prepared in DMSO and stored at −20 °C.

### 4.3 Cell Lines and Viruses

*Macaca mulatta* kidney epithelial cells (LLC-Mk2) were maintained in minimal essential medium with one part of Earle’s MEM (Corning) and two parts of Hank’s MEM (Corning) supplemented with 6% fetal bovine serum (FBS, Thermo Scientific) and an Antibiotic Antimycotic Solution (Thermo Scientific). Mouse L cells stably transfected with mCEACAM-receptor (LR7) were maintained in Dulbecco’s modified Eagle’s medium (Corning) supplemented with 5% FBS, Antibiotic Antimycotic Solution, and 0.5 mg/mL G418 (Gibco). Human embryonic kidney cells (HEK293T) and HEK293T cells stably producing ACE2 receptor^44^ were maintained in Dulbecco’s modified Eagle’s medium (Corning) supplemented with 8% FBS, 100 IU/ml penicillin, and 100 μg/mL streptomycin. SARS-CoV-2 replicon containing BHK21 cell line was maintained in Dulbecco’s modified Eagle’s medium supplemented with 8% FBS, 100 IU/ml penicillin, 100 μg/mL streptomycin, and 200 μg/mL G418.

Vero cells (ATCC CCL-81) and Vero E6 cells (ATCC CRL-1586) were cultured in Dulbecco’s modified Eagle’s medium (DMEM). The media were supplemented with 10% (DMEM) newborn calf serum, 100 U/mL penicillin, 100 μg/mL streptomycin, and 1% glutamine (Sigma-Aldrich, Prague, Czech Republic). Vero and Vero E6 cells were cultured at 37 °C with 5% CO_2_.

HCoV-NL63 (isolate Amsterdam 1) and MHV strain A59, a kind gift of Prof. Krzysztof Pyr (Malopolska Centre of Biotechnology, Jagiellonian University, Poland), were propagated in LLC-Mk2 and LR7, respectively. Infected LLC-Mk2 cells were incubated for 9 days at 32 °C under 5% CO_2_. LR7 cells infected with MHV were incubated at 37 °C under 5% CO_2_ for 2 days. Virus stocks were prepared at the indicated time points by lysing the cells in two freeze–thaw rounds. Medium containing the virus was aliquoted and stored in −70 °C. Virus titer was determined by the plaque assay.

SARS-CoV-2 (strain SARS-CoV-2/human/Czech Republic/951/2020) was isolated from a clinical specimen at the National Institute of Health, Prague, Czech Republic, and was kindly provided by Dr. Jan Weber, Institute of Organic Chemistry and Biochemistry, Prague, Czech Republic. Vero cells were used for SARS-CoV-2 propagation and for anti-SARS-CoV-2 assays, whereas Vero E6 cells were used for the plaque assay.

### 4.4 Cell Viability Assay

LR7, LLC-Mk2 or Vero cells were seeded in 96-well plates and cultured overnight. Next, the medium was changed to a new one supplemented with different concentrations (in triplicate) of analyzed compounds. Following the 2 days (LR7 and Vero) or 4 days (LLC-Mk-2) of incubation, CellTiter 96 AQ_ueous_ nonradioactive cell proliferation assay (Promega) was performed according to the manufacturer’s instruction. The half-maximal cytotoxic concentration (CC_50_) was calculated as the concentration of a compound needed to reduce cell viability by 50% using GraphPad Prism software (version 5.01, GraphPad Software, San Diego, CA).

### 4.5 Reduction of Virus Cytopathic Effect

LR7 cells were seeded in a 96-well plate and cultured overnight. Next, the cells were infected with MHV at an MOI value of 0.01 in medium without FBS. Following 1 h of incubation, the medium containing the virus was collected and cells were covered with a medium supplemented with different concentrations (in triplicate) of the analyzed compounds or DMSO. 24 h p.i. cells viability was evaluated by CellTiter 96 AQ_ueous_ nonradioactive cell proliferation assay (Promega) according to the manufacturer’s instruction.

### 4.6 Dose-Dependent Anticoronavirus Activity of Compounds

LR7 or LLC-Mk2 cells were infected at a MOI of 0.01 with MHV and HCoV-NL63, respectively, and treated with selected compounds at a concentration ranging from 0 to 50 μM. Culture supernatants were collected 1 day (MHV) or 4 days (HCoV-NL63) after infection. Virus titer was determined by plaque assay.

Vero cells were seeded in appropriate plates and allowed to reach an approximately 90% confluence. For infection, 200 μL of viral suspension containing 10^5^ pfu (SARS-CoV-2 strain SARS-CoV-2/human/Czech Republic/951/2020) was added to each well and incubated for 2 h at 37 °C to allow virus adsorption. After the incubation, the viral medium was removed, and the wells were washed gently with phosphate-buffered saline (PBS). Test compounds were prepared in serial dilutions to final concentrations of 50, 25, 12.5, 6.25, 3.125, and 0 μM in the medium (DMEM containing 2% FBS). The compounds were added in quadruplicate to the infected cells. The plates were then incubated for 48 h at 37 °C. Following the 48 h treatment period, the supernatants were removed, and plaque assays in Vero E6 cells were performed to assess viral titers. Cells were fixed and stained 4 days after infection to visualize plaques, as described previously.^45^

The half-maximum inhibitory concentration value (IC_50_) indicating the concentration of compound needed to reduce virus titer by 50% in comparison to the control, was evaluated by GraphPad software (version 5.01, GraphPad Software, San Diego, CA).

### 4.7 Western Blot Analysis

LLC-Mk2 monolayers grown in 24-well plates were infected with HCoV-NL63 at an MOI value of 0.01 for 2 h. Unbound virus was removed, and cells were cultured in medium supplemented with different concentrations of compounds. Four days postinfection, cells were collected and lysed in CellLyticM Cell Lysis Reagent (Sigma). Following the protein separation by SDS-PAGE and transfer to PVDF membranes, proteins were immunoblotted using specific mouse anti-N NL63 Abs (Eurofins, Spain) or rabbit anti-β-catenin Abs (Santa Cruz), followed by antimouse or antirabbit secondary antibodies conjugated with HRP. Proteins were visualized and analyzed by chemiluminescence using the UVITEC Cambridge Alliance MINI HD detection system. The densitometric analysis of cells treated with 50 μM dose of a panel of glycoconjugates was performed based on three biological replicates of N and β-catenin immunoblots. The N protein levels were expressed as a percentage relative to DMSO-treated infected cells, while β-catenin levels were expressed as a percentage relative to noninfected control cells. The data were presented as the ratio of N/β-catenin for each sample, normalized accordingly.

### 4.8 SARS-CoV-2 VLP Internalization Assay

SARS-CoV-2 VLP internalization assay was performed according to the protocol published by Syed et al. with some modifications.^32^ Seven × 10^5^ HEK293T cells were seeded into each well of the six-well plate. Next day, cells on each well were transfected with 1 μg of LucPS9 (addgene no. 177942), 1.35 μg of CoV-2-N-WT-Hu1 (addgene no. 177937), 0.6 μg of CoV2-M-IRES-E (addgene no. 177938), and 50 ng of CoV2-Spike-EF1a-D614G-N501Y (addgen no. 177939) using jetPRIME transfection reagent according to the manufacturer’s protocol. Briefly, DNA was mixed with 200 μL of jetPRIME buffer, and subsequently, 6 μL of jetPRIME reagent was added to the mixture, vortexed, and incubated for 10 min. Next transfection mixture was added by droplets onto cells. 4 h post-transfection (p.t.) medium was changed to one supplemented with different concentrations of the analyzed compounds. 48 h p.t. SC-VLP-containing medium was collected and filtered by a 0.45 μm PVDF filter. Remaining cells were collected and lysed in CellLytic buffer (Sigma) or 1% digitonin.

50 μL of SC-VLP containing medium was added to 3 × 10^4^ HEK293T or HEK293T hACE2 cells freshly seeded on a 96-well plate in 50 μL of medium and incubated. After 24 h, the cells were lysed using 100 μL per well of Glo Lysis Buffer (Promega). Luciferase expression was analyzed by Bright-Glo Luciferase Assay (Promega) according to the manufacturer’s protocol.

SC-VLPs were precipitated using 10% PEG6000 with 0.3 M NaCl and resuspended in 30 μL of PBS buffer. Following the protein separation by SDS-PAGE and transfer to PVDF membranes, proteins were immunoblotted using specific rabbit anti-S2 Abs (Sino Biological Europe GmbH, Germany) or rabbit anti-β-catenin Abs (Santa Cruz) followed by antirabbit secondary antibodies conjugated with HRP. Proteins were visualized by chemiluminescence using the UVITEC Cambridge Alliance MINI HD detection system.

### 4.9 Viral RNA Isolation and PCR Assay

LR7 or LLC-Mk2 were cultured in 12-well plate overnight. Next, LR7 cells were infected with MHV at an MOI value of 1 for 1 h or LLC-Mk2 cells were infected with HCoV-NL63 at an MOI value of 0.01 for 2 h. Following removal of unbound virus, cells were cultured with medium supplemented with different concentrations of selected compounds. 8 h p.i. (MHV) or 96 h p.i. (HCoV-NL63) cells were collected and total RNA was isolated by CoV RNA kit (A&A Biotechnology, Poland) according to the manufacturer’s instruction. To evaluate levels of viral RNA in infected cells Qiagen One-Step RT-PCR reaction was performed. Following reverse transcription, the PCR reaction was carried out using primers: F: GATGGTGTTGTTTGGGTTGC, R: CTGTGGAAAACCTTTGGCATC for amplification of a fragment of HCoV-NL63 genome (position: 26418–26960 bp NC_005831.2) and F: TGGCCGAAGAAATTGCTGCTCTTG and R: TGCACAGAGCTTTTGGGCTTTGC for amplification of a fragment of MHV genome (position: 30341–30902 bp FJ647225.1). For internal control fragment of β-actin mRNA was reverse transcribed and amplified using primers For: GCGGGAAATCGTGCGTGACATT and Rev: GATGGAGTTGAAGGTAGTTTCGTG.

### 4.10 Inhibition of SARS-CoV-2 Replication

The protocol for SARS-CoV-2 replicon containing BHK21 cell line treatment was conducted according to Liu et al.^34^ In brief: Compounds were diluted in DMEM cell culture medium with 8% FBS to a final concentration of 25 μM. The SARS-CoV-2-Rep-NanoLuc-Neo BHK21 cells were seeded at 1.5 × 10^4^ per well in a 96-well plate in triplicate and incubated at 37 °C, 5% CO_2_. 24 h after seeding, the cell culture medium was replaced with the media containing compounds and incubated at 37 °C, 5% CO_2_ for 48 h. After that time, the cells were assayed for NanoLuc activity with Nano-Glo Luciferase Assay System (Promega), and luminescence signal was assessed in a microplate reader (Infinite 200, Tecan). All compounds were tested in at least three biological replicates.

### 4.11 Statistical Analysis

All statistical analyses were performed using GraphPad Prism 5 software. Statistical significance was determined using a *t* test or Dunnett’s One-way ANOVA test.

## Supplementary Material





## Notes

1H NMR spectra and HRMS of analyzed compounds. Immunodetection of S and N proteins in SC-VLP-producing cells (PDF)
A file containing the compound numbers described in the publication, along with their corresponding Molecular Formula Strings, has been included in the submitted materials (CSV)

## Abbreviations Used

β4GalT: β-1,4-galactosyltransferase
ACE2: angiotensin-converting enzyme 2
BHK21: baby hamster kidney 21 (cells)
CPE: cytopathic effect
CuAAC: copper­(i)-catalyzed 1,3-dipolar azido-alkyne cycloaddition
DMEM: Dulbecco’s modified Eagle’s medium
DMV: double membrane vesicle
FBS: fetal bovine serum
GT: glycosyltransferase
hCoV: human coronavirus
HRP: horseradish peroxidase
LLC-Mk2: *Macaca mulatta* kidney epithelial cells
mCEACAM: murine carcinoembryonic antigen-related cell adhesion molecule
MEM: minimal essential medium
MERS-CoV: middle east respiratory syndrome coronavirus
MHV: murine hepatitis virus
MOI: multiplicity of infection
MTS: 3-(4,5-dimethylthiazol-2-yl)-5-(3-carboxymethoxyphenyl)-2-(4-sulfophenyl)-2H-tetrazolium
NaAsc: sodium ascorbate
N: nucleocapsid (protein)
PFU: plaque-forming unit
PMS: phenazine methosulfate
S: spike (protein)
SARS-CoV: severe acute respiratory syndrome coronavirus
SARS-CoV-2: severe acute respiratory syndrome coronavirus 2
SI: selectivity index
TBDMS: *tert*-butyldimethylsilyl
VLP: virus-like particle
