# Supplementary material for: Anticoronavirus Activity of Uridine Glycoconjugates Containing a 1,2,3-Triazole Moiety
Source: J Med Chem. 2025 Aug 8;68(16):17859–73. doi: 10.1021/acs.jmedchem.5c01602 (PMC12406196; doi:10.1021/acs.jmedchem.5c01602)
Supplement: Supplementary file 1 [file jm5c01602_si_001.pdf]

Supporting Information

Anti-coronavirus activity of uridine glycoconjugates containing 1,2,3-triazole moiety

Malgorzata Graul[1,2], Gabriela Brzuska[1], Ewa Wisniewska[1], Monika Dominska[3,4], Petra Strakova[5], Daniel Ruzek[5,6,7], Gabriela Pastuch-Gawolek[3,4], Ewelina Krol[1,*]

[1] Laboratory of Recombinant Vaccines, Intercollegiate Faculty of Biotechnology, University of Gdansk and Medical University of Gdansk, Abrahama 58, 80-307 Gdansk, Poland; malgorzata.graul@umu.se (M.G.); gabriela.brzuska@ug.edu.pl (G.B.); ewa.wisniewska@phdstud.ug.edu.pl (E.W.); ewelina.krol@biotech.ug.edu.pl (E.K.)

[2] Laboratory of Virus Molecular Biology, Intercollegiate Faculty of Biotechnology, University of Gdansk and Medical University of Gdansk, Abrahama 58, 80-307 Gdansk, Poland

[3] Department of Organic Chemistry, Bioorganic Chemistry and Biotechnology, Faculty of Chemistry, Silesian University of Technology, Krzywoustego 4, 44-100 Gliwice, Poland; monikamdominska@gmail.com(M.D.); gabriela.pastuch@polsl.pl (G.P-G.)

[4] Biotechnology Center, Silesian University of Technology, Krzywoustego 8, 44-100 Gliwice, Poland

[5] Laboratory of Emerging Viral Diseases, Veterinary Research Institute, Hudcova 70, CZ-62100 Brno, Czech Republic; petra.strakova@vri.cz (P.S.); ruzekd@paru.cas.cz (D.R.)

[6] Department of Experimental Biology, Faculty of Science, Masaryk University, Kamenice 735, CZ-62500 Brno, Czech Republic

[7] Laboratory of Emerging Viral Infections, Veterinary Research Institute, Hudcova 296, CZ-62100 Brno, Czech Republic

TABLE OF CONTENTS

1. *NMR Spectra of Uridine Glycoconjugates (14-25)* *S2*
Fig. S1: ^{1}H NMR spectrum of compound **14**............ **S3**
Fig. S2: ^{13}C NMR spectrum of compound **14**............ **S4**
Fig. S3: ^{1}H NMR spectrum of compound **15**............ **S5**
Fig. S4: ^{13}C NMR spectrum of compound **15**............ **S6**
Fig. S5: ^{1}H NMR spectrum of compound **16**............ **S7**
Fig. S6: ^{13}C NMR spectrum of compound **16**............ **S8**
Fig. S7: ^{1}H NMR spectrum of compound **17**............ **S9**
Fig. S8: ^{13}C NMR spectrum of compound **17**............ **S10**
Fig. S9: ^{1}H NMR spectrum of compound **18**............ **S11**
Fig. S10: ^{13}C NMR spectrum of compound **18**............ ***S12***
Fig. S11: ^{1}H NMR spectrum of compound **19**............ **S13**
Fig. S12: ^{13}C NMR spectrum of compound 19............ **S14**
Fig. S13: ^{1}H NMR spectrum of compound **20**............ **S15**
Fig. S14: ^{13}C NMR spectrum of compound **20**............ **S16**
Fig. S15: ^{1}H NMR spectrum of compound **21**............ **S17**
Fig. S16: ^{13}C NMR spectrum of compound **21**............ **S18**
Fig. S17: ^{1}H NMR spectrum of compound **22**............ **S19**
Fig. S18: ^{13}C NMR spectrum of compound **22**............ **S20**
Fig. S19: ^{1}H NMR spectrum of compound **23**............ **S21**

Fig. S20: ^{13}C NMR spectrum of compound **23**……………………………………………………………**S22**
Fig. S21: ^{1}H NMR spectrum of compound **24**……………………………………………………………**S23**
Fig. S22: ^{13}C NMR spectrum of compound **24**……………………………………………………………**S24**
Fig. S23: ^{1}H NMR spectrum of compound **25**……………………………………………………………**S25**
Fig. S24: ^{13}C NMR spectrum of compound **25**……………………………………………………………**S26**
2. ***HRMS of Uridine Glycoconjugates (14-25***)…………………………………………………*S27*
Fig. S25: HRMS of compound **14**……………………………………………………………………S27
Fig. S26: HRMS of compound **15**……………………………………………………………………S28
Fig. S27: HRMS of compound **16**……………………………………………………………………S29
Fig. S28: HRMS of compound **17**……………………………………………………………………S30
Fig. S29: HRMS of compound **18**……………………………………………………………………S31
Fig. S30: HRMS of compound **19**……………………………………………………………………S32
Fig. S31 HRMS of compound **20**……………………………………………………………………S33
Fig. S32: HRMS of compound **21**……………………………………………………………………S34
Fig. S33: HRMS of compound **22**……………………………………………………………………S35
Fig. S34: HRMS of compound **23**……………………………………………………………………S36
Fig. S35: HRMS of compound **24**……………………………………………………………………S37
Fig. S36: HRMS of compound **25**……………………………………………………………………S38
3. ***Immunodetection of S and N proteins in SC-VLP producing cells. (Fig. S37)*……………………S39**

1. NMR Spectra of Uridine Glycoconjugates (**14**-**25**)

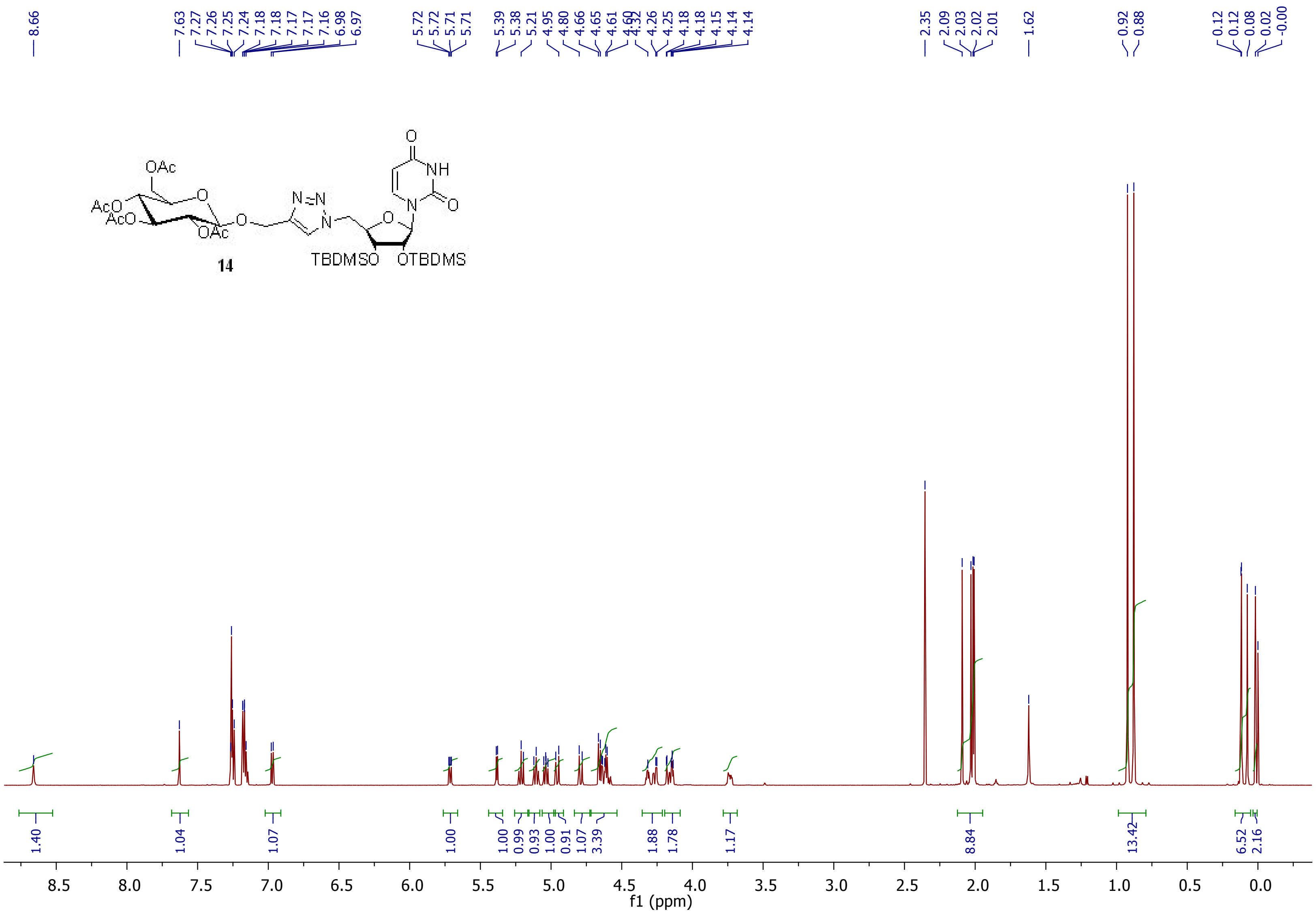

Fig. S1: ^{1}H NMR spectrum of compound **14**.

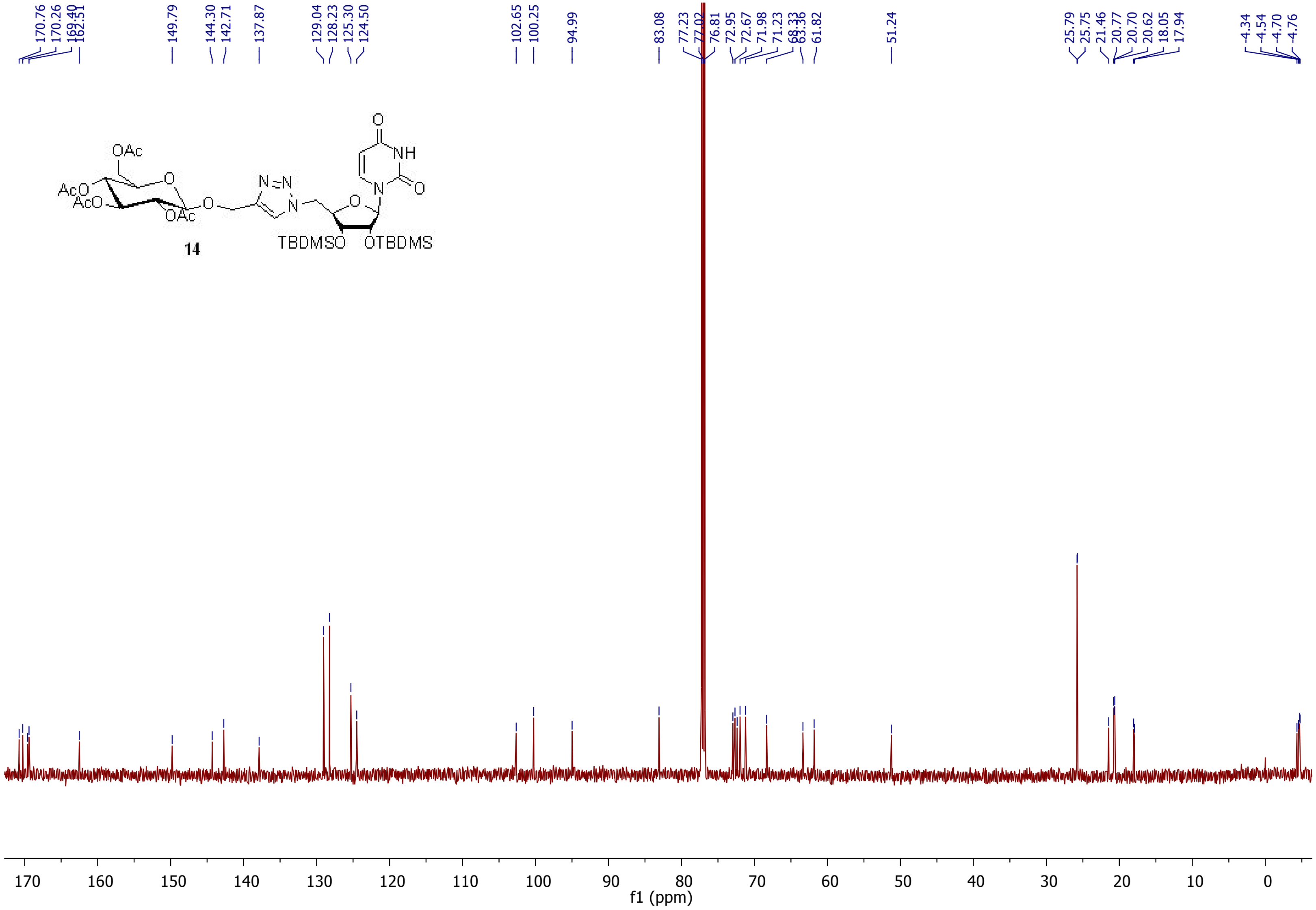

Fig. S2: ^{13}C NMR spectrum of compound **14**.

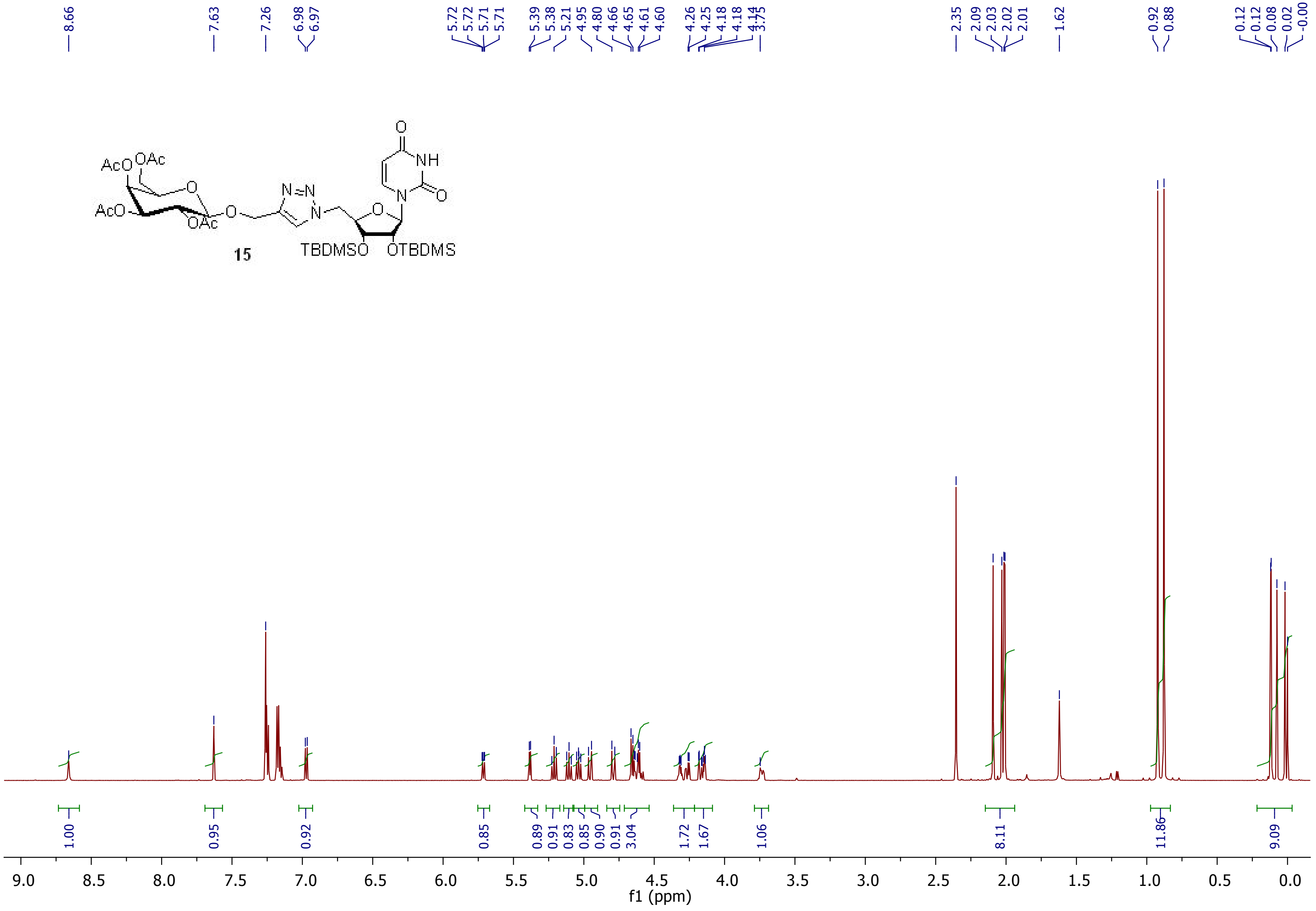

Fig. S3: ^{1}H NMR spectrum of compound **15**.

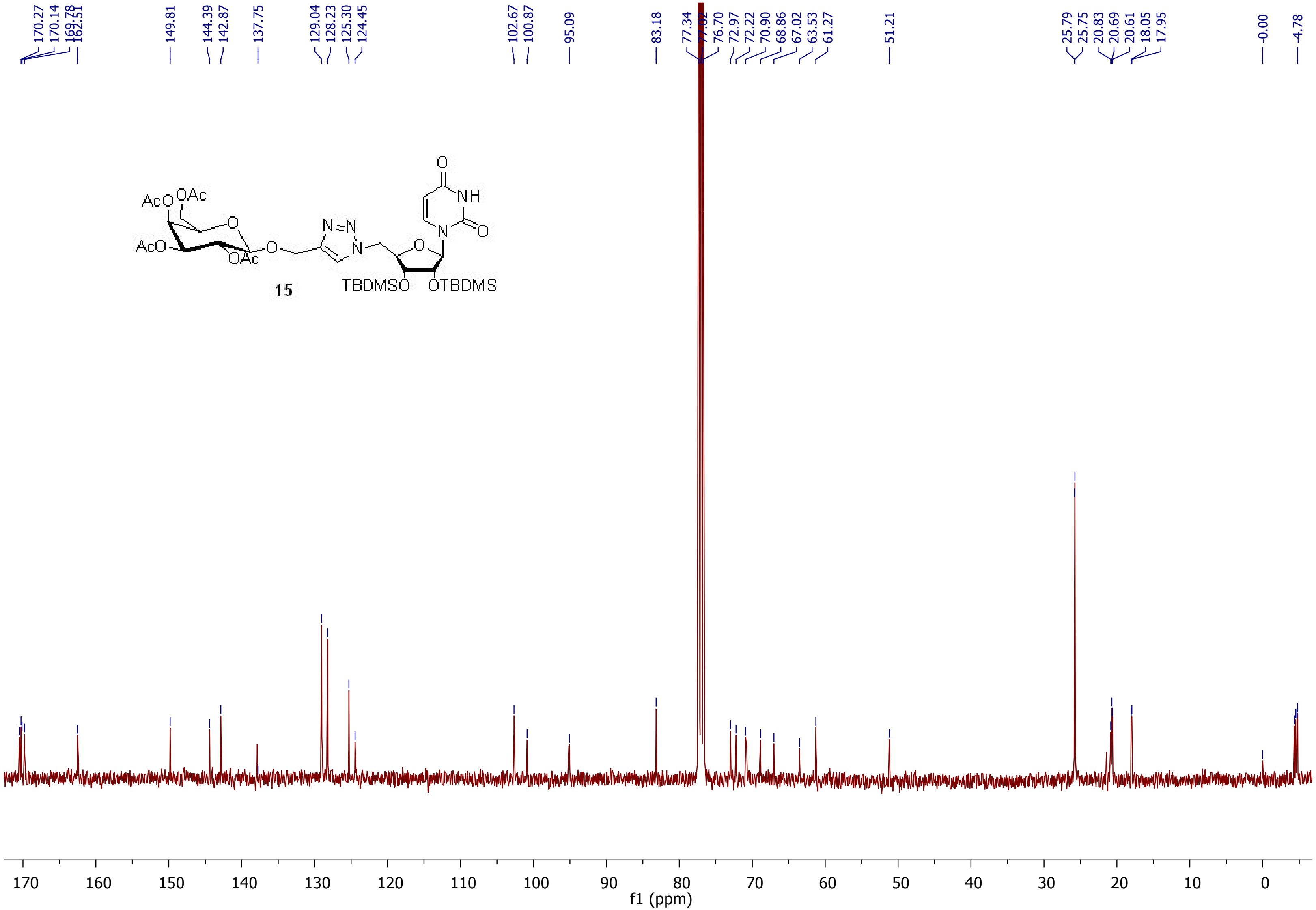

Fig. S4: ^{13}C NMR spectrum of compound **15**.

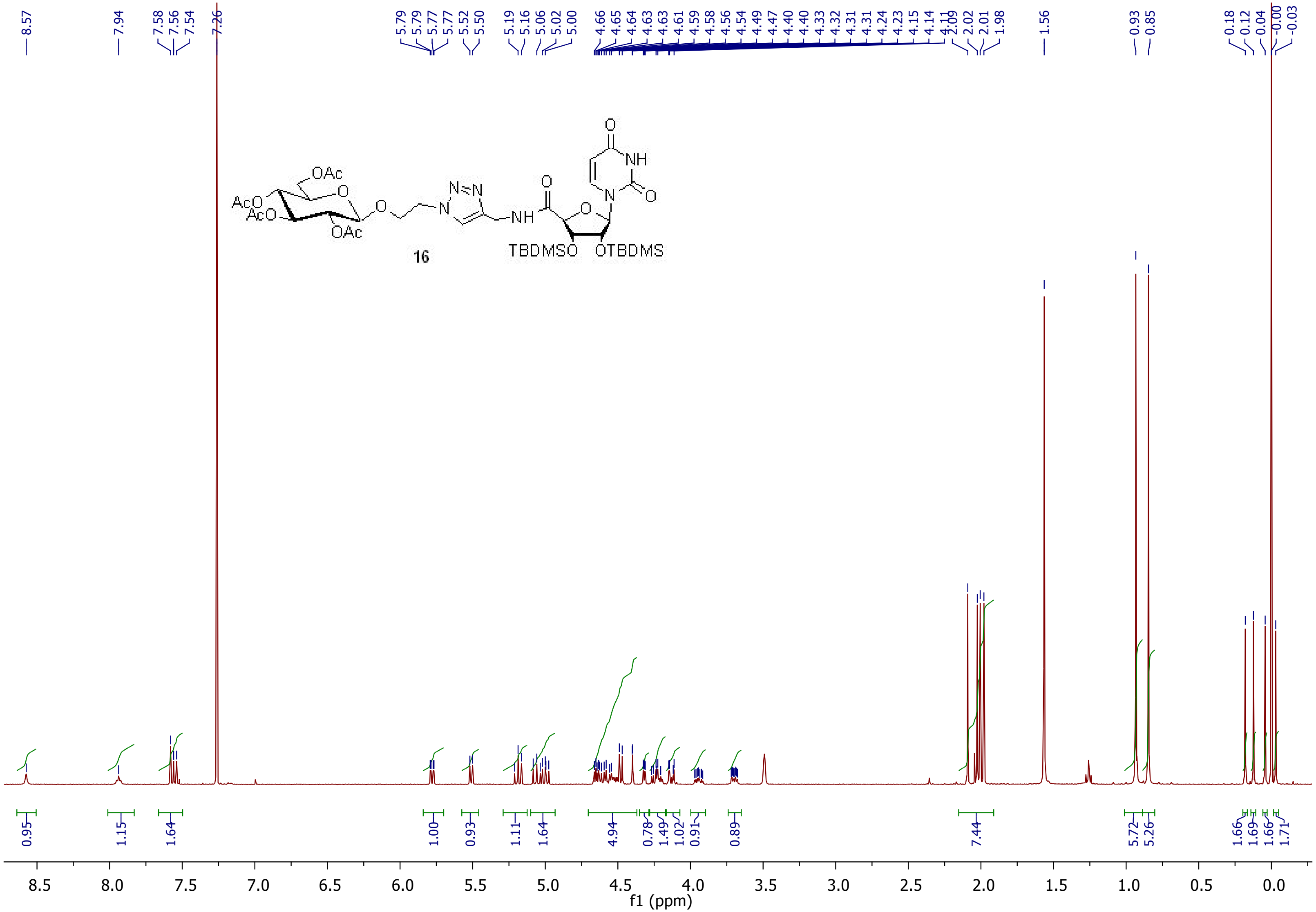

Fig. S5: ^{1}H NMR spectrum of compound **16**.

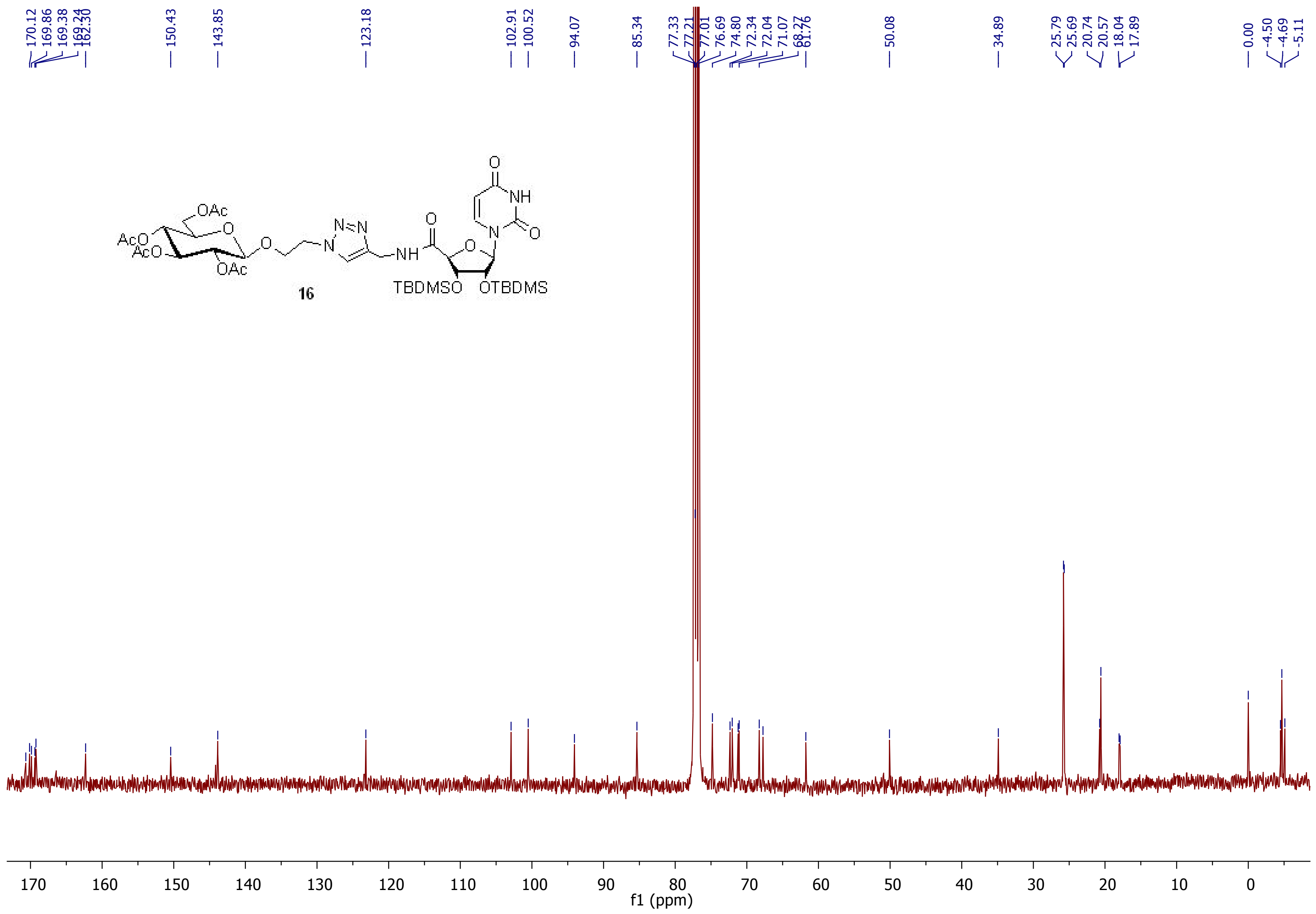

Fig. S6: ^{13}C NMR spectrum of compound **16**.

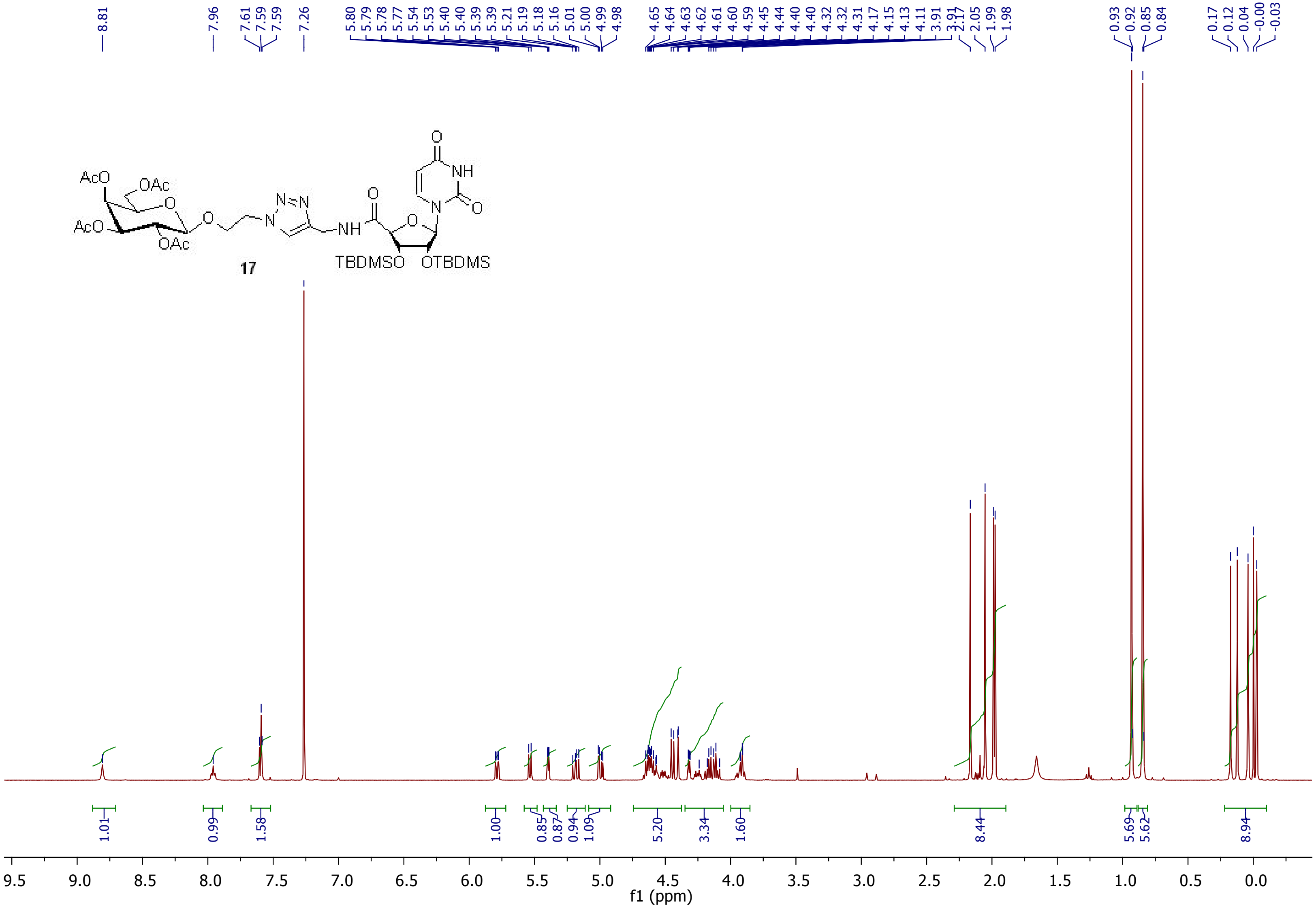

Fig. S7: ^{1}H NMR spectrum of compound **17**.

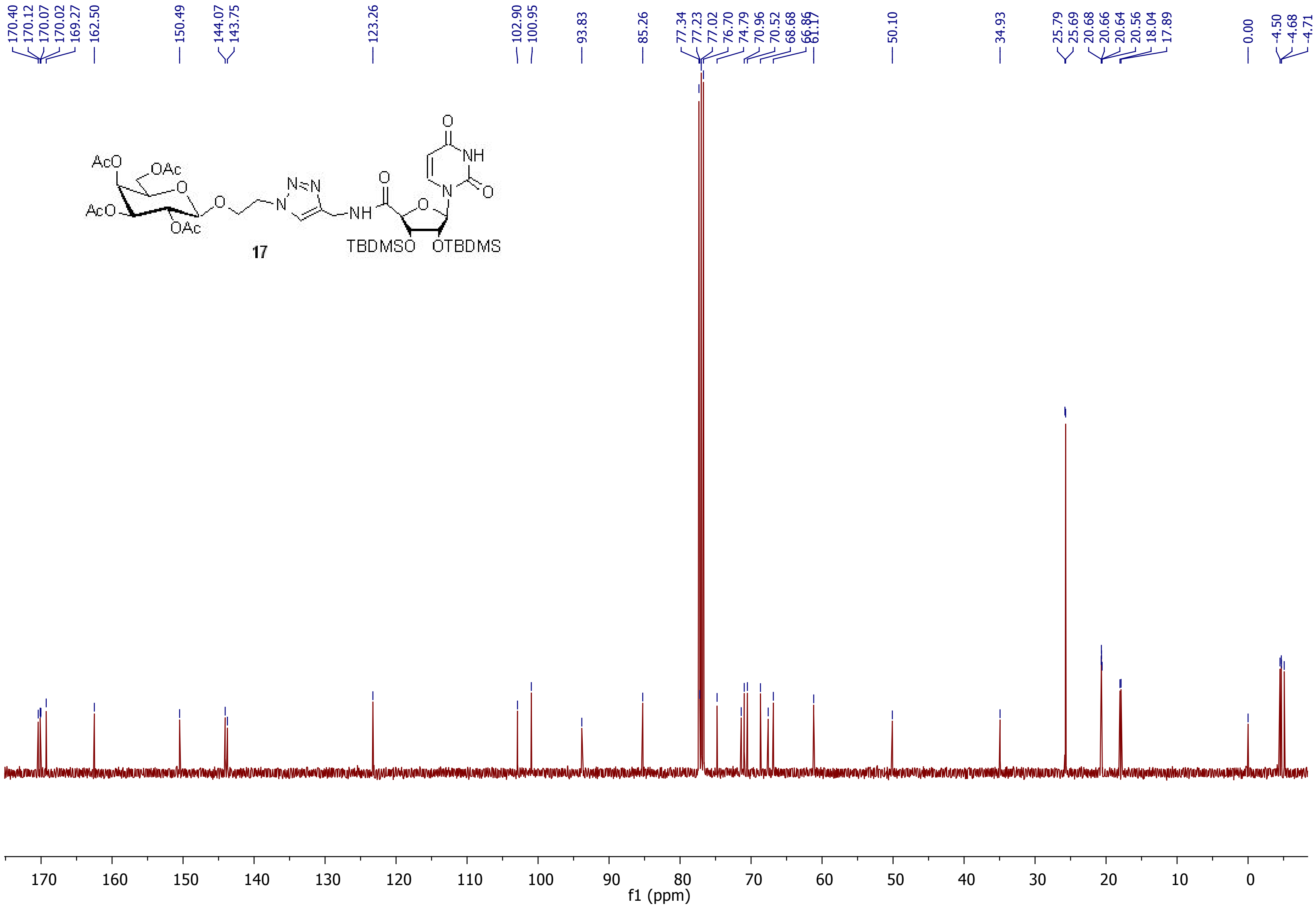

Fig. S8: ^{13}C NMR spectrum of compound **17**.

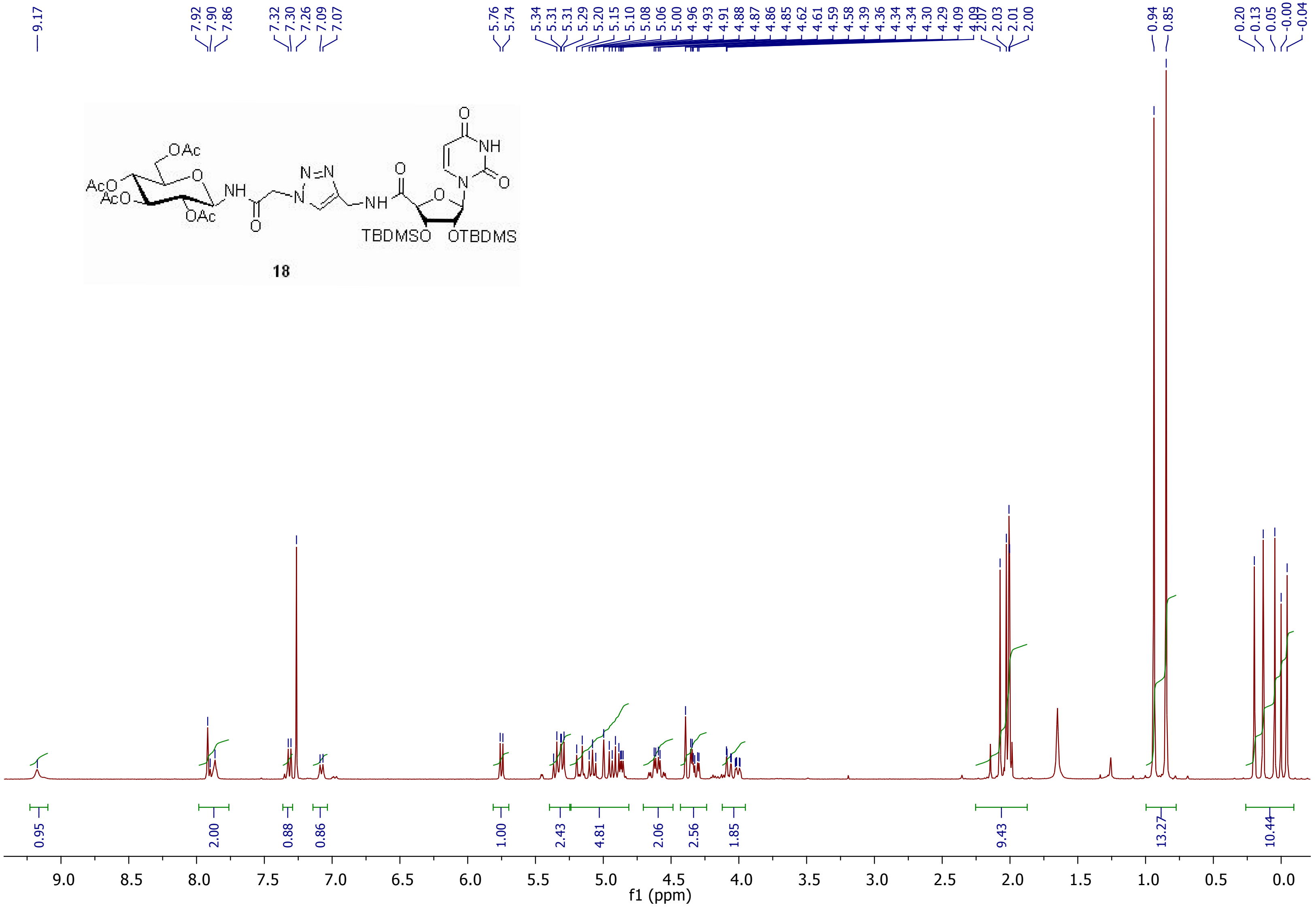

Fig. S9: ^{1}H NMR spectrum of compound **18**.

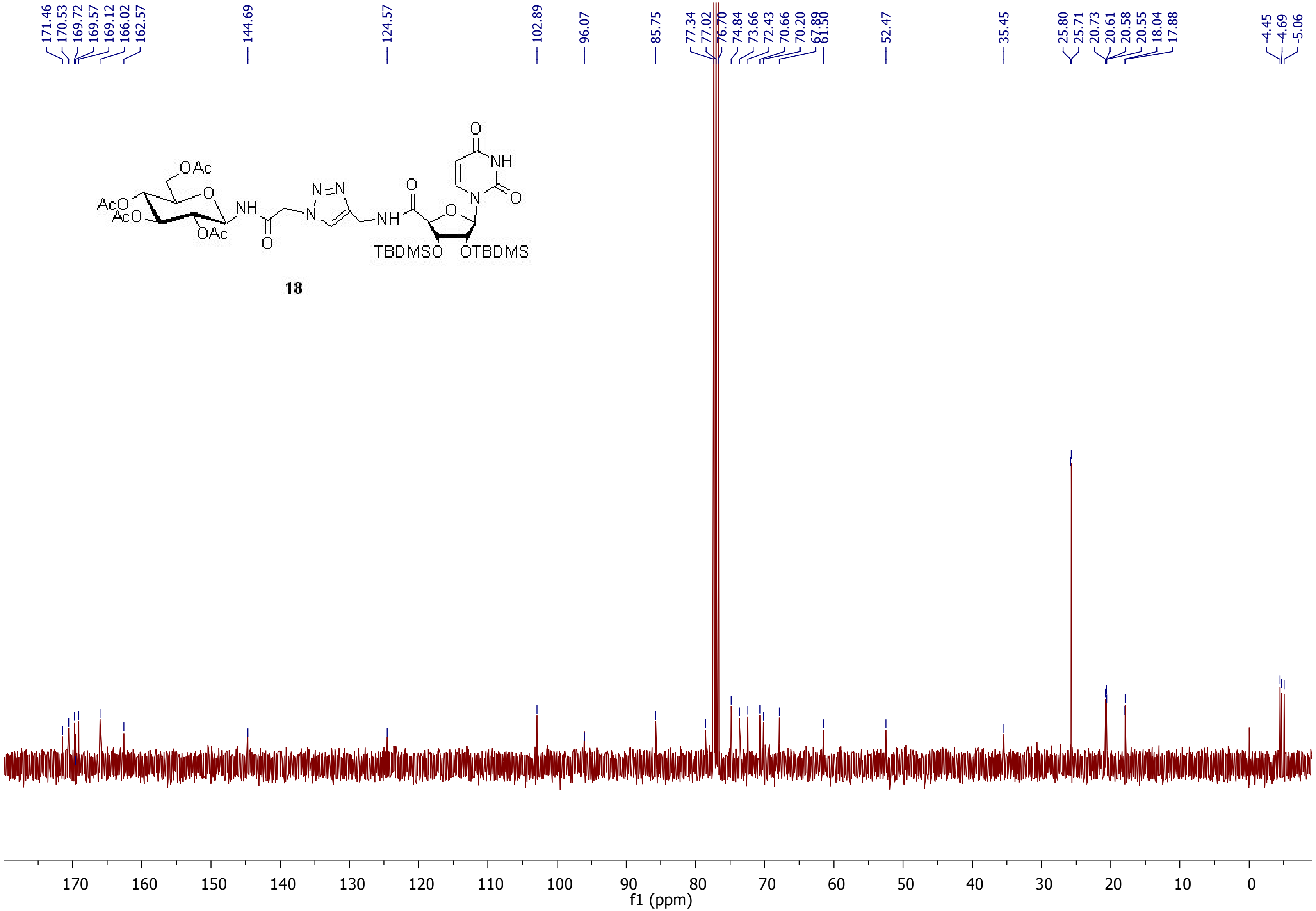

Fig. S10: ^{13}C NMR spectrum of compound **18**.

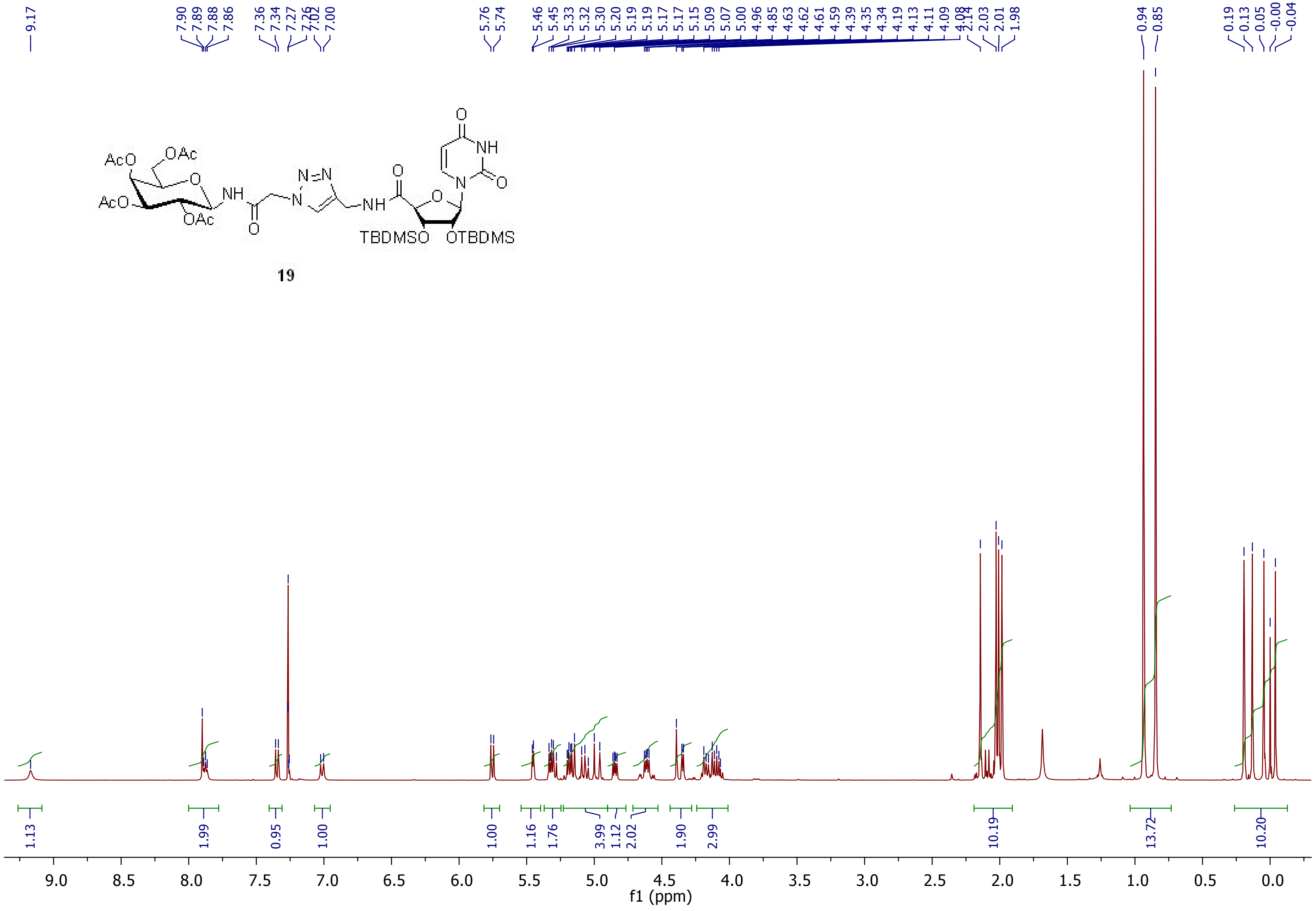

Fig. S11: ^{1}H NMR spectrum of compound **19**.

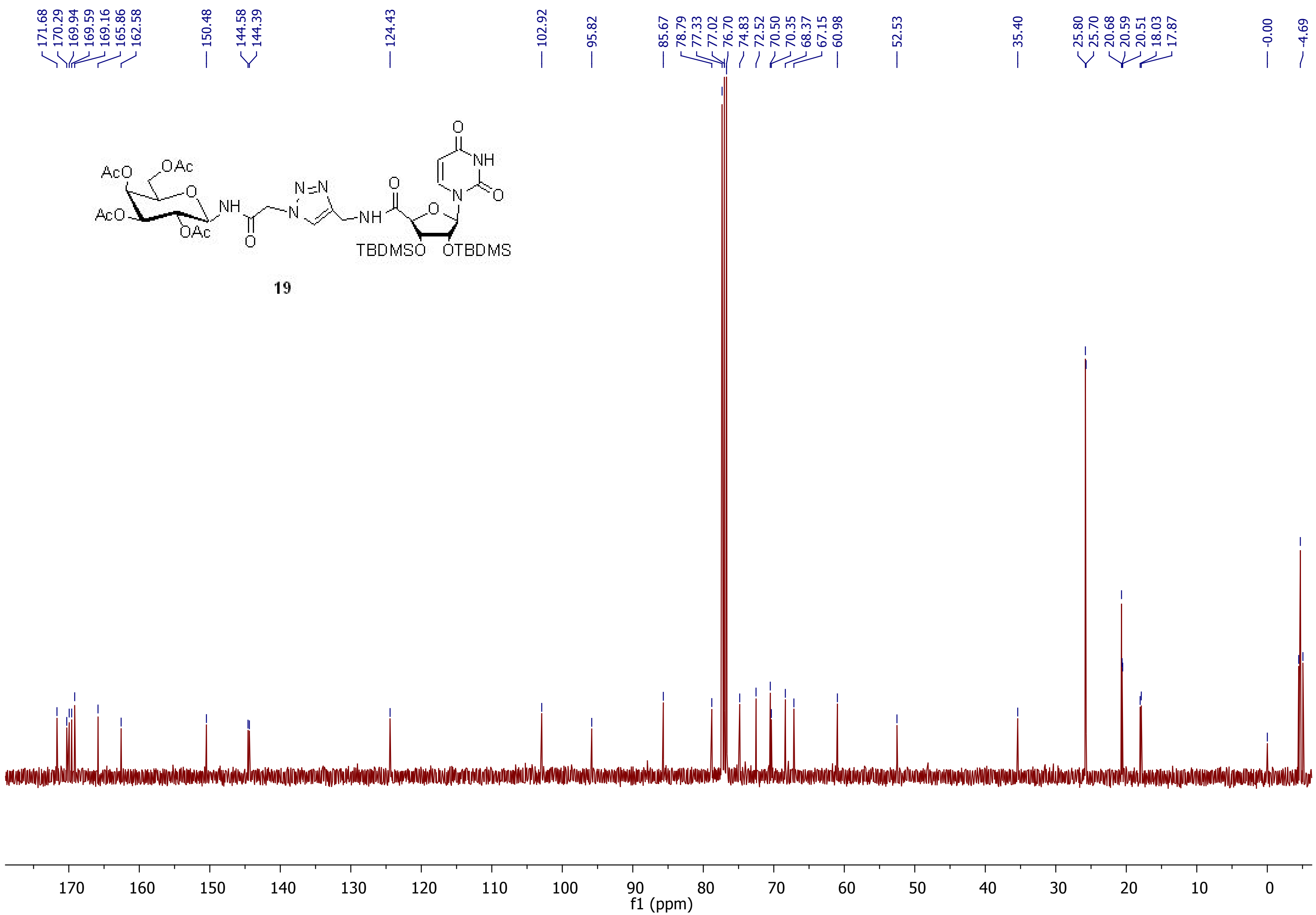

Fig. S12: ^{13}C NMR spectrum of compound **19**.

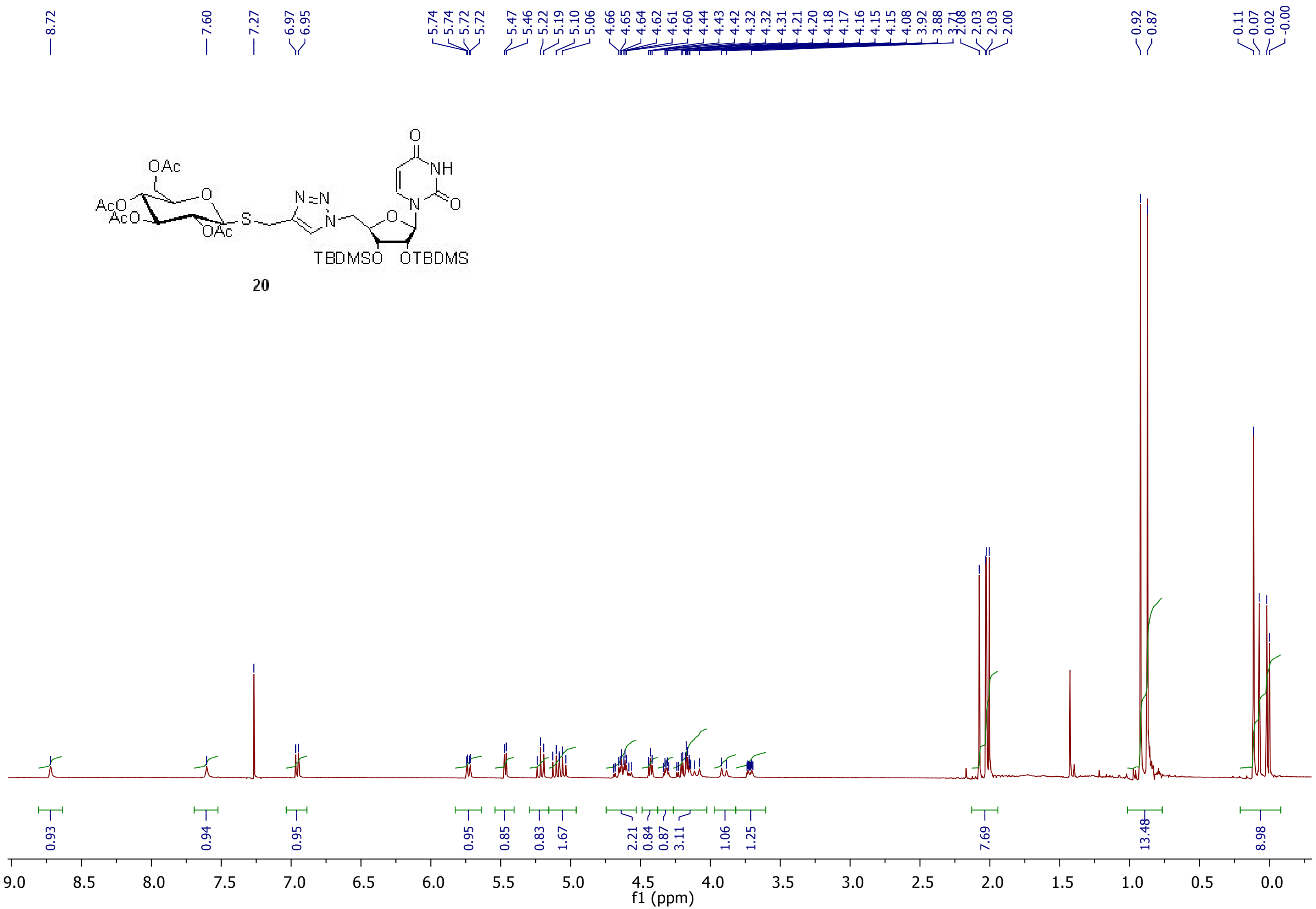

Fig. S13: ^{1}H NMR spectrum of compound **20**.

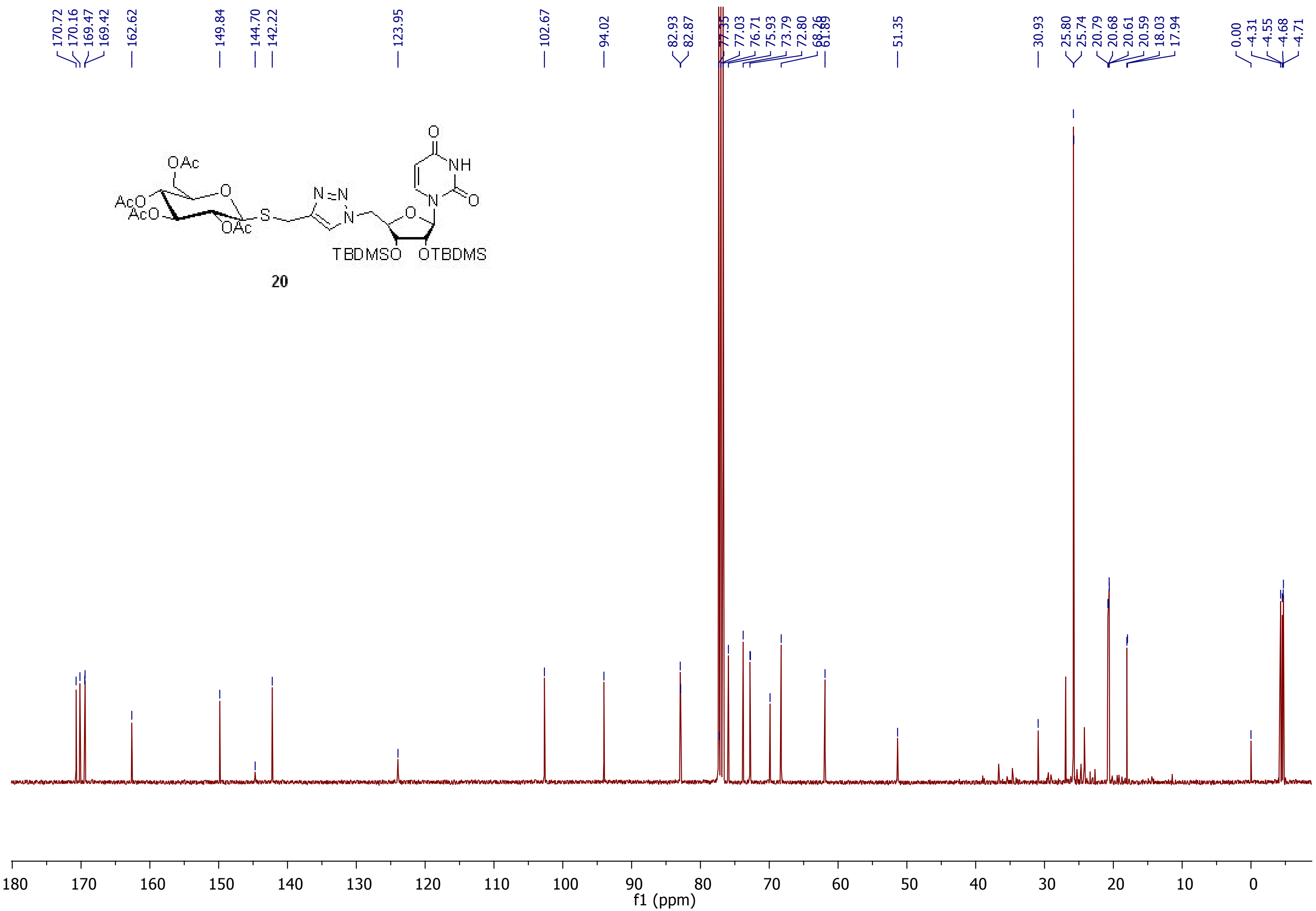

Fig. S14: ^{13}C NMR spectrum of compound **20**.

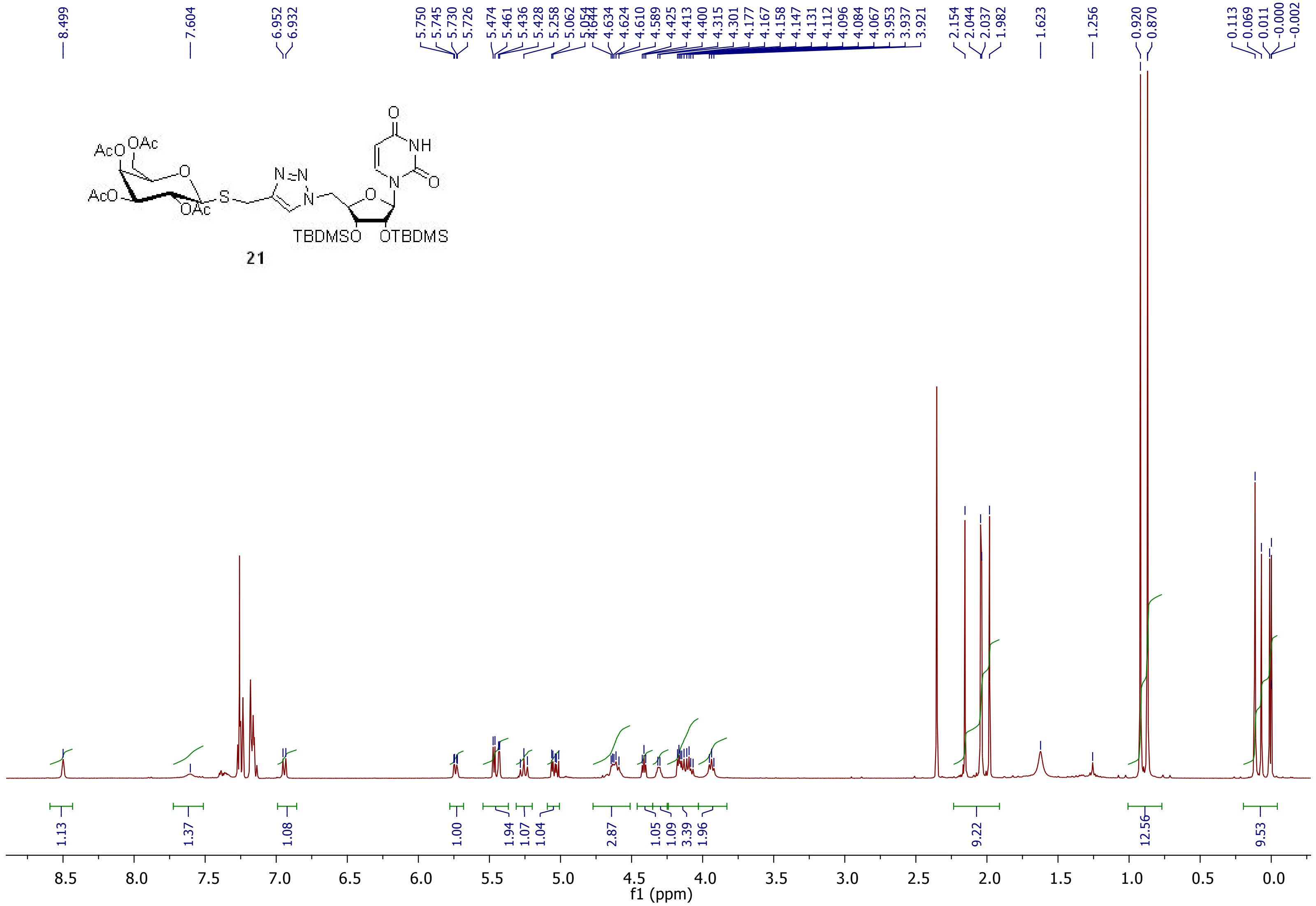

Fig. S15: ^{1}H NMR spectrum of compound **21**.

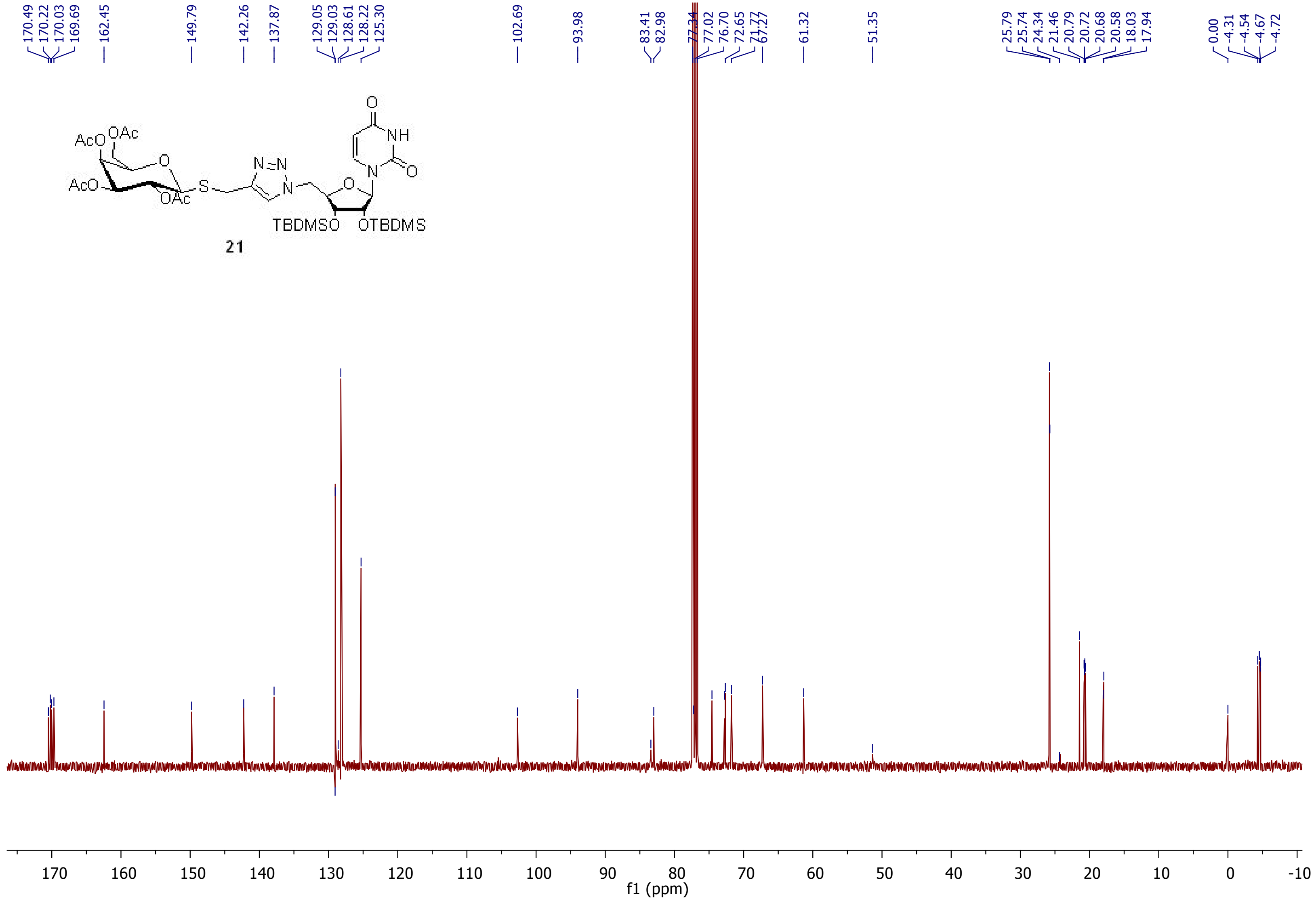

Fig. S16: ^{13}C NMR spectrum of compound **21**.

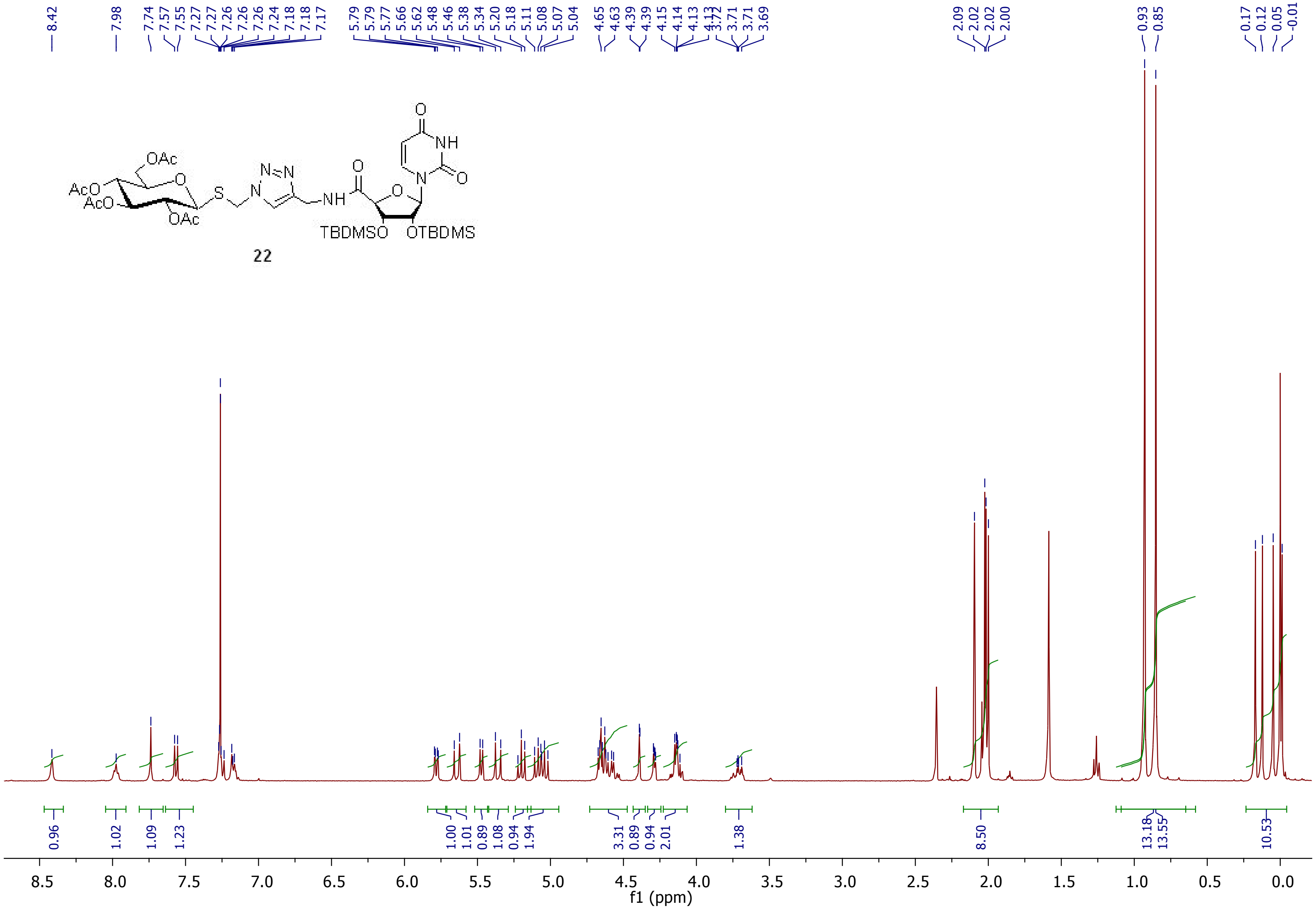

Fig. S17: ^{1}H NMR spectrum of compound **22**.

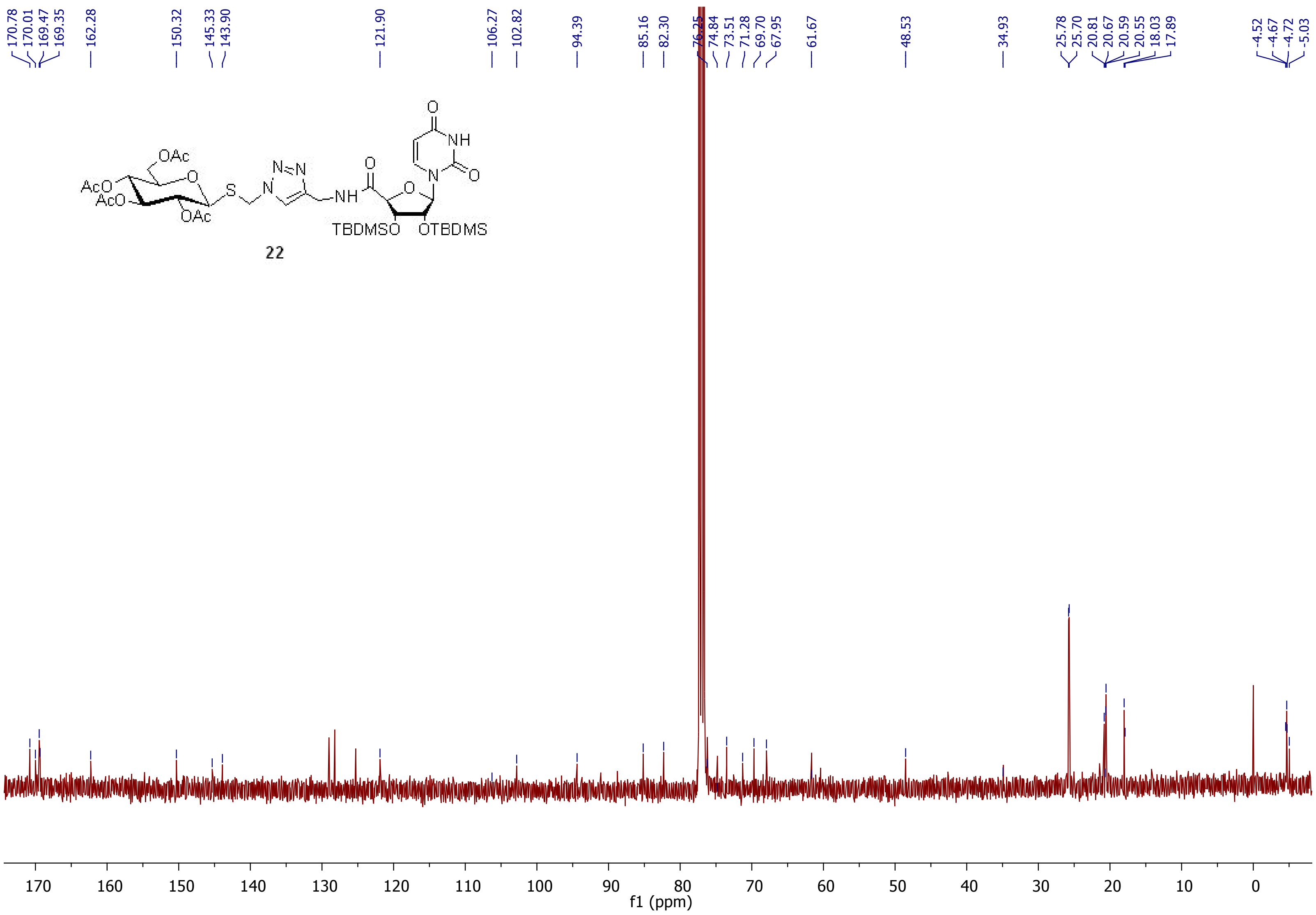

Fig. S18: ^{13}C NMR spectrum of compound **22**.

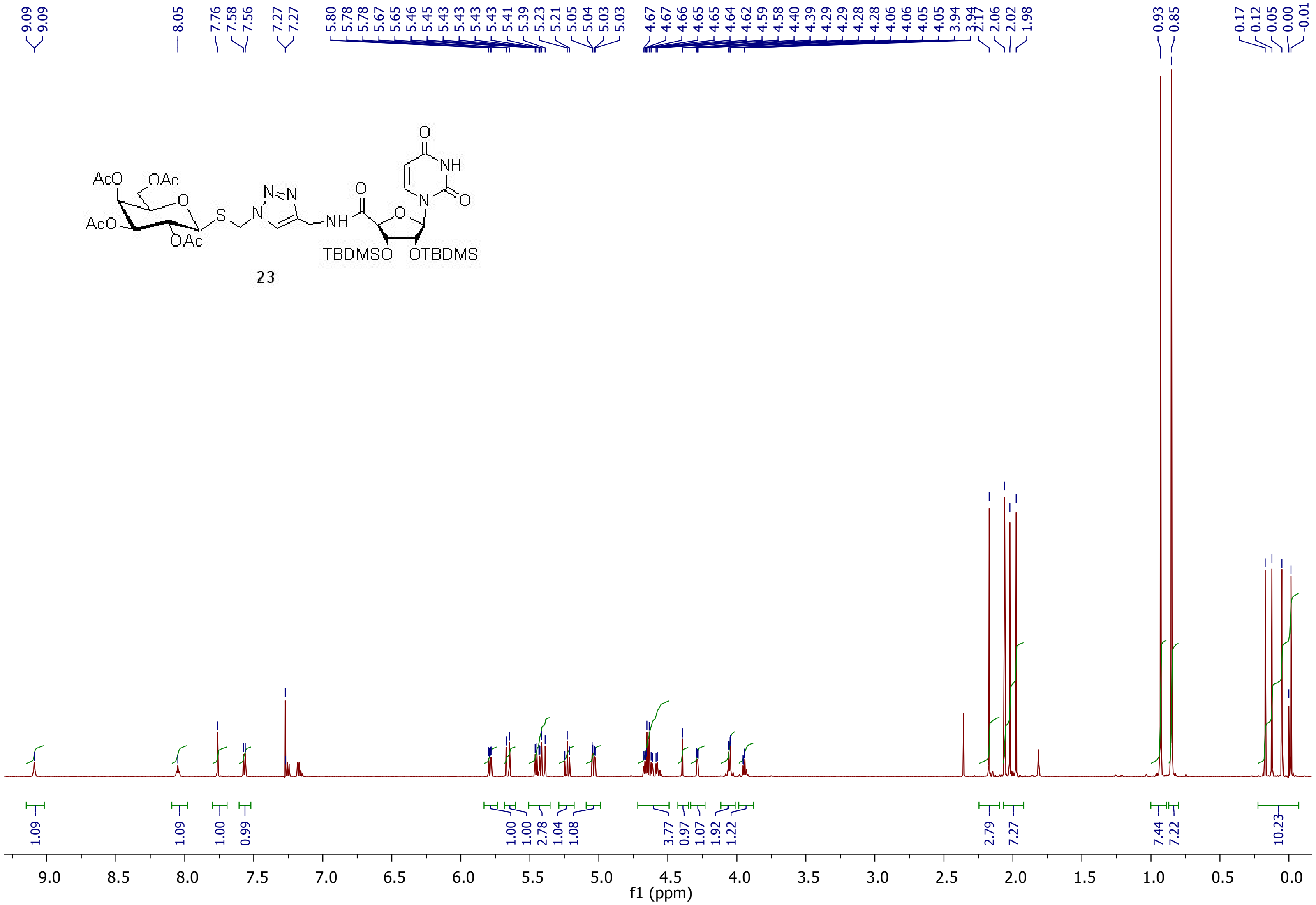

Fig. S19: ^{1}H NMR spectrum of compound **23**.

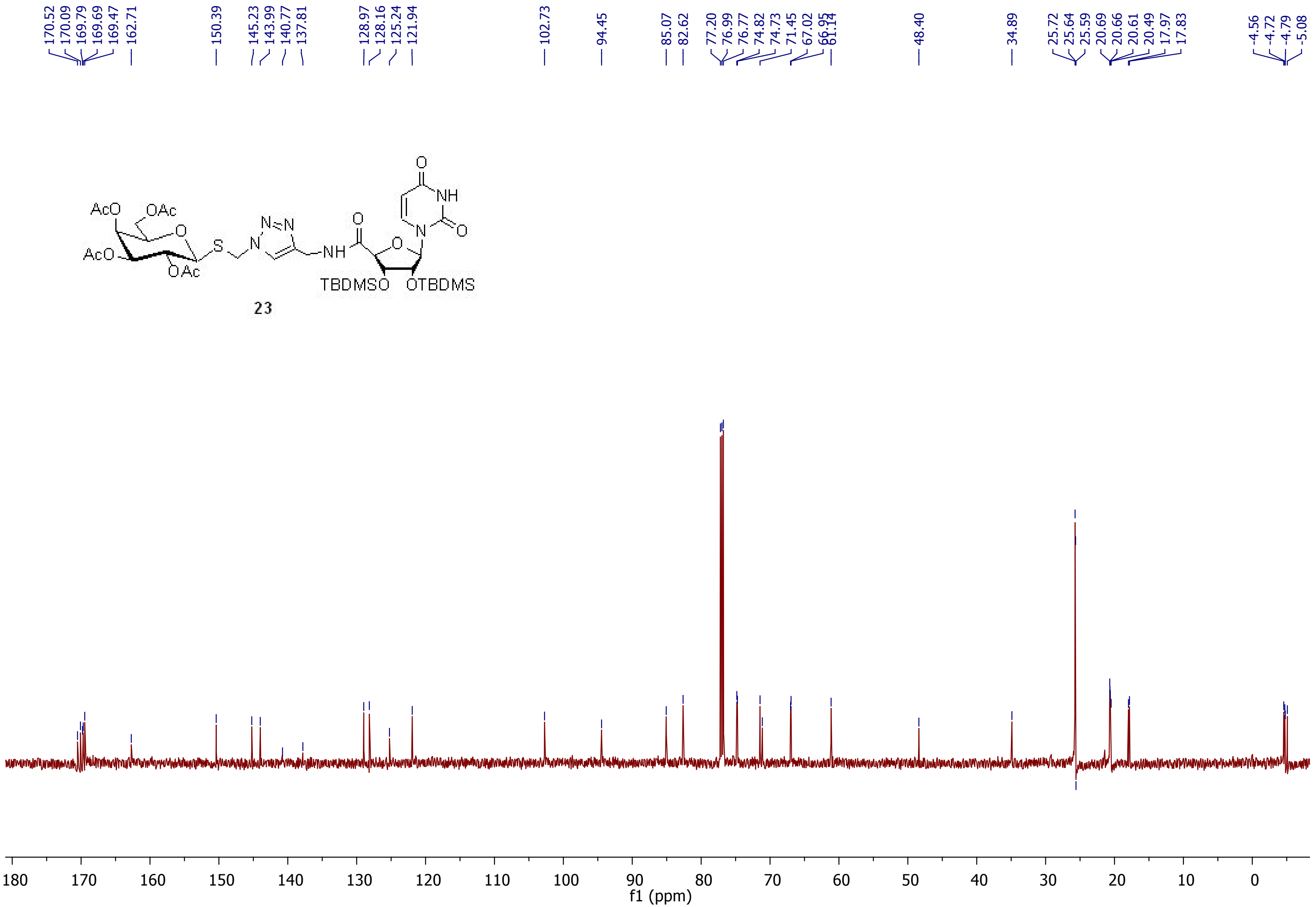

Fig. S20: ^{13}C NMR spectrum of compound **23**.

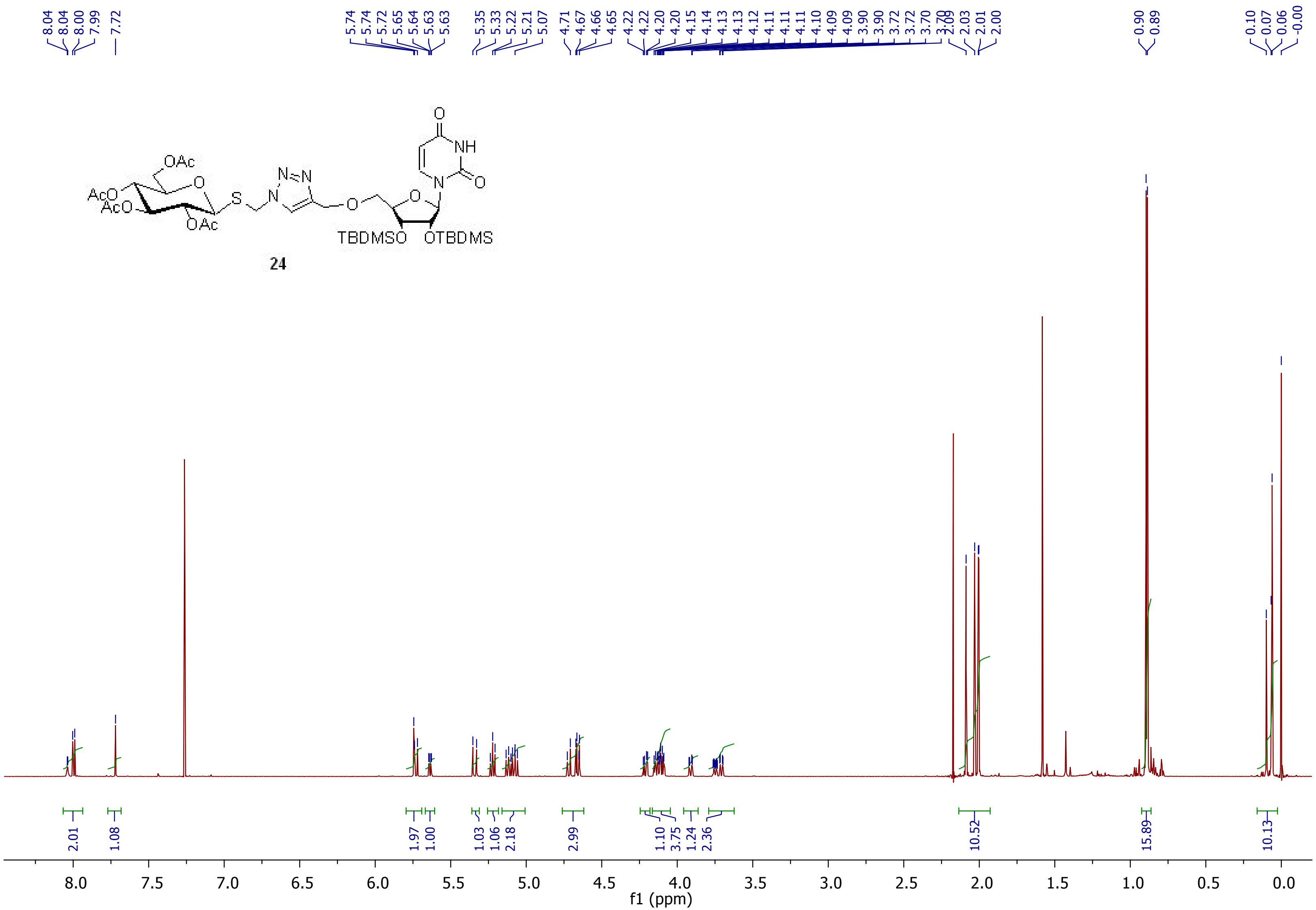

Fig. S21: ^{1}H NMR spectrum of compound **24**.

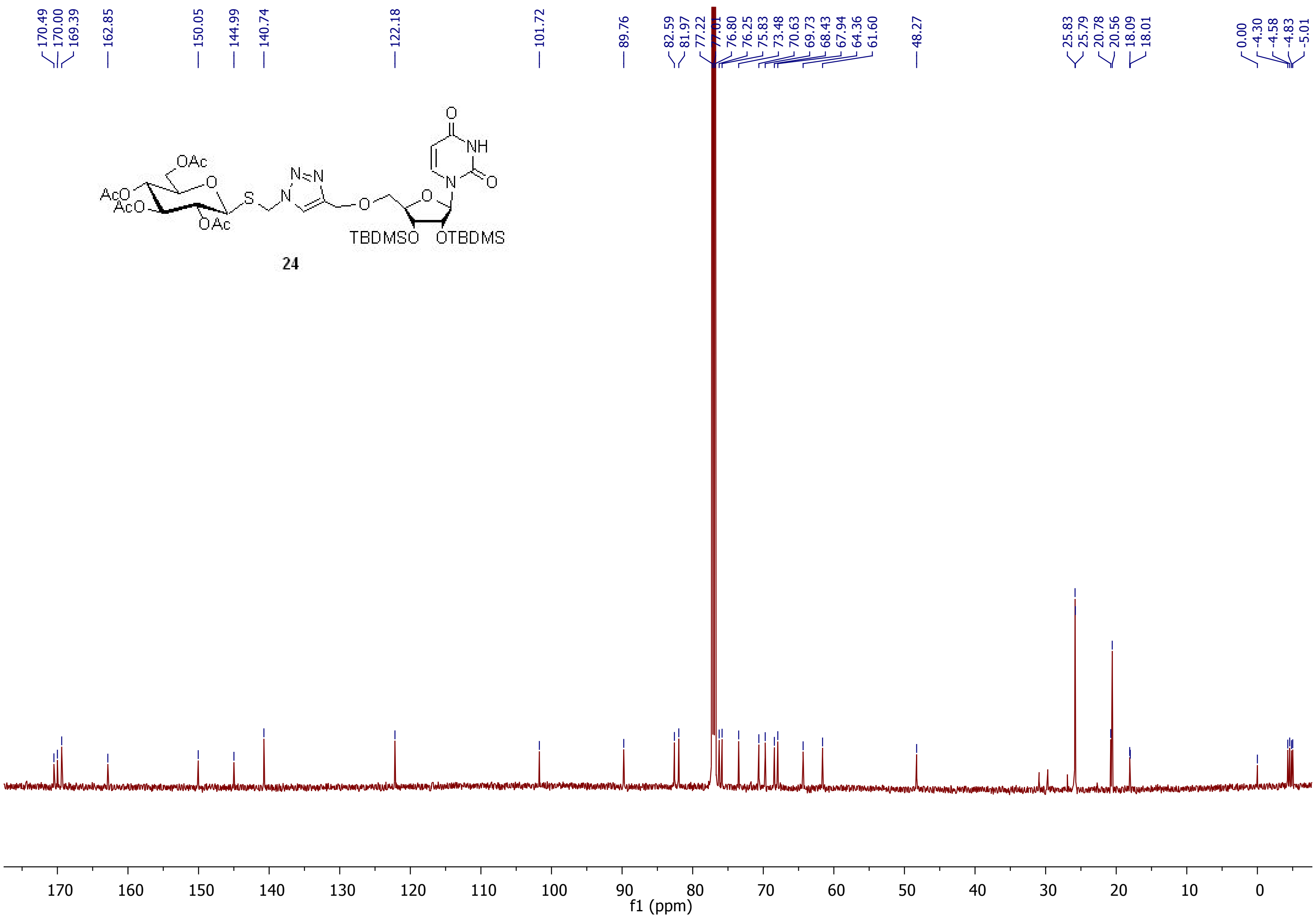

Fig. S22: ^{13}C NMR spectrum of compound **24**.

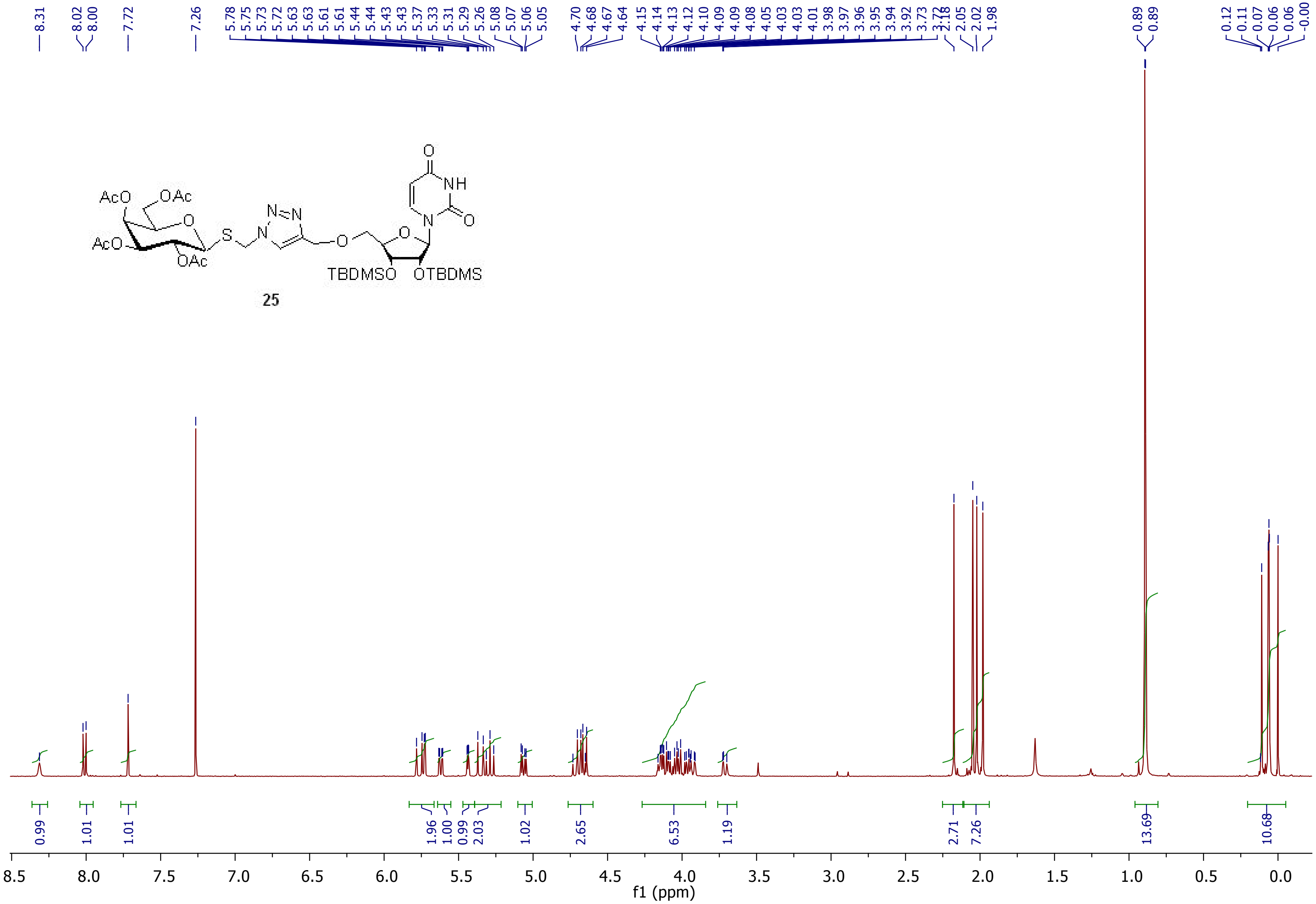

Fig. S23: ^{1}H NMR spectrum of compound **25**.

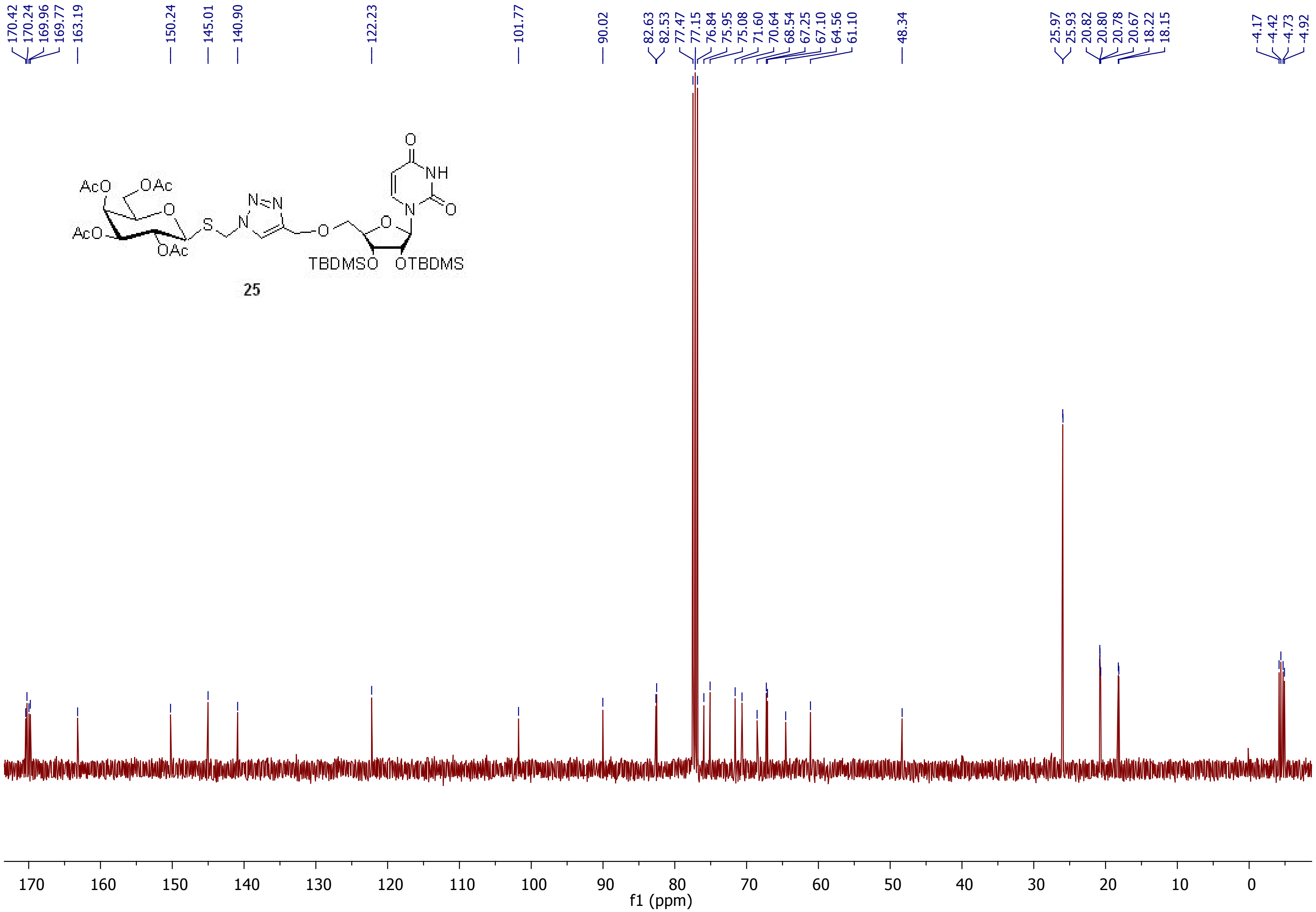

Fig. S24: ^{13}C NMR spectrum of compound **25**.

2. HRMS of Uridine Glycoconjugates (**14**-**25**)

Multiple Mass Analysis: 2 mass(es) processed
Tolerance = 100.0 mDa / DBE: min = -10.0, max = 200.0
Element prediction: Off
Number of isotope peaks used for i-FIT = 3
Monoisotopic Mass, Even Electron Ions
35 formula(e) evaluated with 12 results within limits (up to 3 closest results for each mass)
Elements Used:

Mass	RA	Calc. Mass	mDa	PPM	DBE	Formula	i-FIT	i-FIT Norm	Fit Conf %	C	H	N	O	Na	Si
884.3779	66.79	884.3781	-0.2	-0.2	12.5	C38 H62 N5 O15 Si2	162.9	0.682	50.56	38	62	5	15		2
		884.3757	2.2	2.5	9.5	C36 H63 N5 O15 Na Si2	163.1	0.909	40.30	36	63	5	15	1	2
		884.4696	-91.7	-103.7	2.5	C35 H75 N5 O15 Na Si2	164.6	2.392	9.14	35	75	5	15	1	2
906.3624	100.00	906.3624	0.0	0.0	15.5	C40 H60 N5 O15 Si2	192.1	1.269	28.10	40	60	5	15		2
		906.3600	2.4	2.6	12.5	C38 H61 N5 O15 Na Si2	191.2	0.376	68.67	38	61	5	15	1	2
		906.4539	-91.5	-101.0	5.5	C37 H73 N5 O15 Na Si2	194.3	3.434	3.23	37	73	5	15	1	2

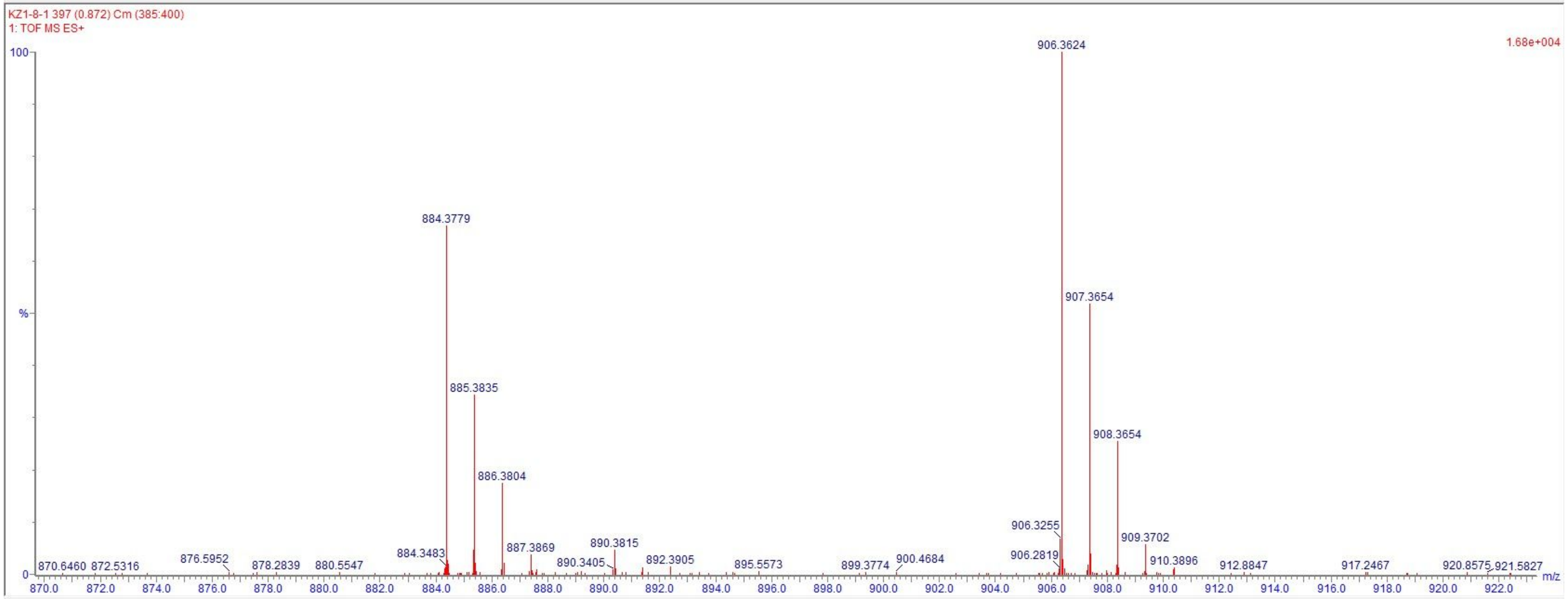

Fig. S25: HRMS of compound **14**

Multiple Mass Analysis: 2 mass(es) processed
Tolerance = 100.0 mDa / DBE: min = -10.0, max = 200.0
Element prediction: Off
Number of isotope peaks used for i-FIT = 3
Monoisotopic Mass, Even Electron Ions
35 formula(e) evaluated with 12 results within limits (up to 3 closest results for each mass)
Elements Used:

Mass	RA	Calc. Mass	mDa	PPM	DBE	Formula	i-FIT	i-FIT Norm	Fit Conf %	C	H	N	O	Na	Si
884.3775	95.30	884.3781	-0.6	-0.7	12.5	C38 H62 N5 O15 Si2	179.3	0.445	64.07	38	62	5	15		2
		884.3757	1.8	2.0	9.5	C36 H63 N5 O15 Na Si2	180.2	1.290	27.53	36	63	5	15	1	2
		884.4696	-92.1	-104.1	2.5	C35 H75 N5 O15 Na Si2	181.4	2.477	8.40	35	75	5	15	1	2
906.3611	100.00	906.3600	1.1	1.2	12.5	C38 H61 N5 O15 Na Si2	185.6	0.389	67.77	38	61	5	15	1	2
		906.3624	-1.3	-1.4	15.5	C40 H60 N5 O15 Si2	186.7	1.404	24.57	40	60	5	15		2
		906.2685	92.6	102.2	22.5	C41 H48 N5 O15 Si2	187.8	2.568	7.67	41	48	5	15		2

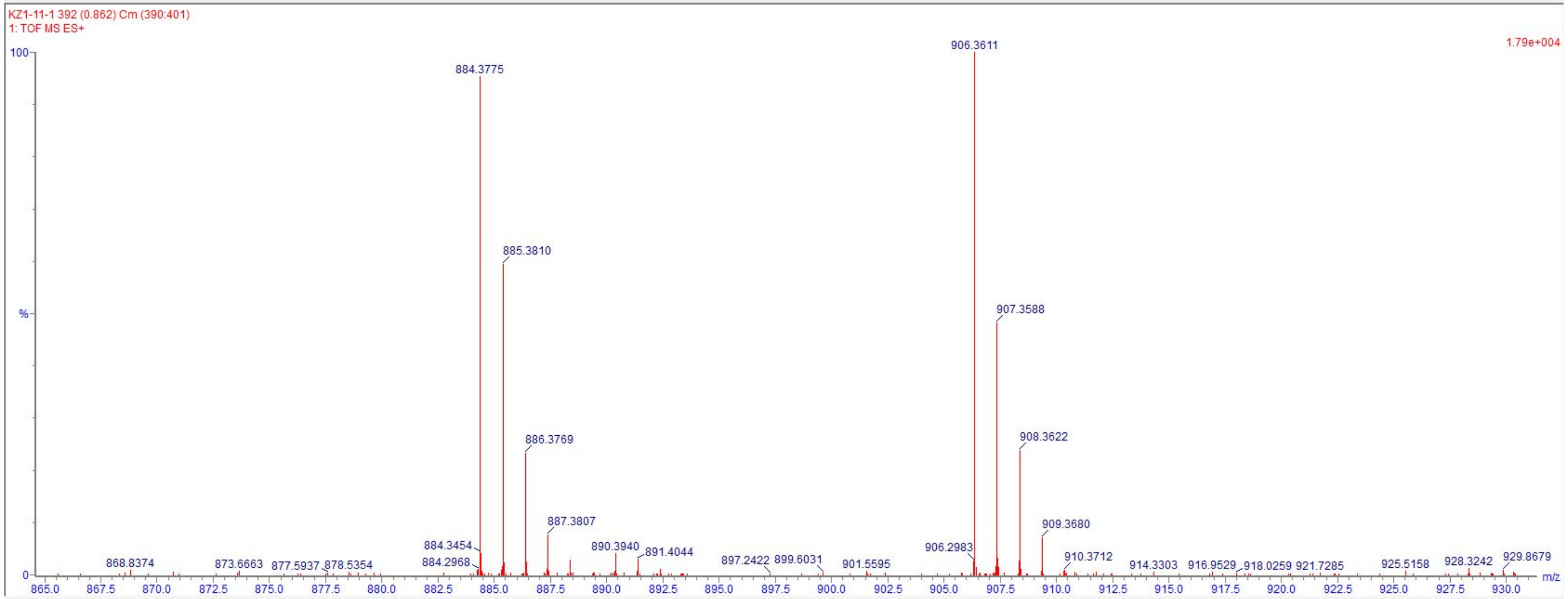

For Help, press F1

Fig. S26: HRMS of compound **15**

Multiple Mass Analysis: 3 mass(es) processed
Tolerance = 100.0 mDa / DBE: min = -10.0, max = 200.0
Element prediction: Off
Number of isotope peaks used for i-FIT = 3
Monoisotopic Mass, Even Electron Ions
196 formula(e) evaluated with 28 results within limits (up to 3 closest results for each mass)
Elements Used:

Mass	RA	Calc. Mass	mDa	PPM	DBE	Formula	i-FIT	i-FIT Norm	Fit Conf %	C	H	N	O	Na	Si
941.3997	56.65	941.3996	0.1	0.1	13.5	C40 H65 N6 O16 Si2	277.2	0.657	51.85	40	65	6	16		2
		941.3972	2.5	2.7	10.5	C38 H66 N6 O16 Na Si2	278.0	1.505	22.20	38	66	6	16	1	2
		941.4335	-33.8	-35.9	9.5	C39 H70 N6 O15 Na Si2	277.9	1.349	25.94	39	70	6	15	1	2
963.3805	100.00	963.3815	-1.0	-1.0	13.5	C40 H64 N6 O16 Na Si2	276.2	0.048	95.30	40	64	6	16	1	2
		963.3839	-3.4	-3.5	16.5	C42 H63 N6 O16 Si2	281.3	5.195	0.55	42	63	6	16		2
		963.4179	-37.4	-38.8	12.5	C41 H68 N6 O15 Na Si2	279.3	3.184	4.14	41	68	6	15	1	2
964.3849	56.51	964.4019	-17.0	-17.6	12.5	C41 H67 N5 O16 Na Si2	256.3	0.796	45.11	41	67	5	16	1	2
		964.4043	-19.4	-20.1	15.5	C43 H66 N5 O16 Si2	256.6	1.139	32.03	43	66	5	16		2
		964.3468	38.1	39.5	21.5	C45 H58 N5 O15 Si2	256.9	1.476	22.86	45	58	5	15		2

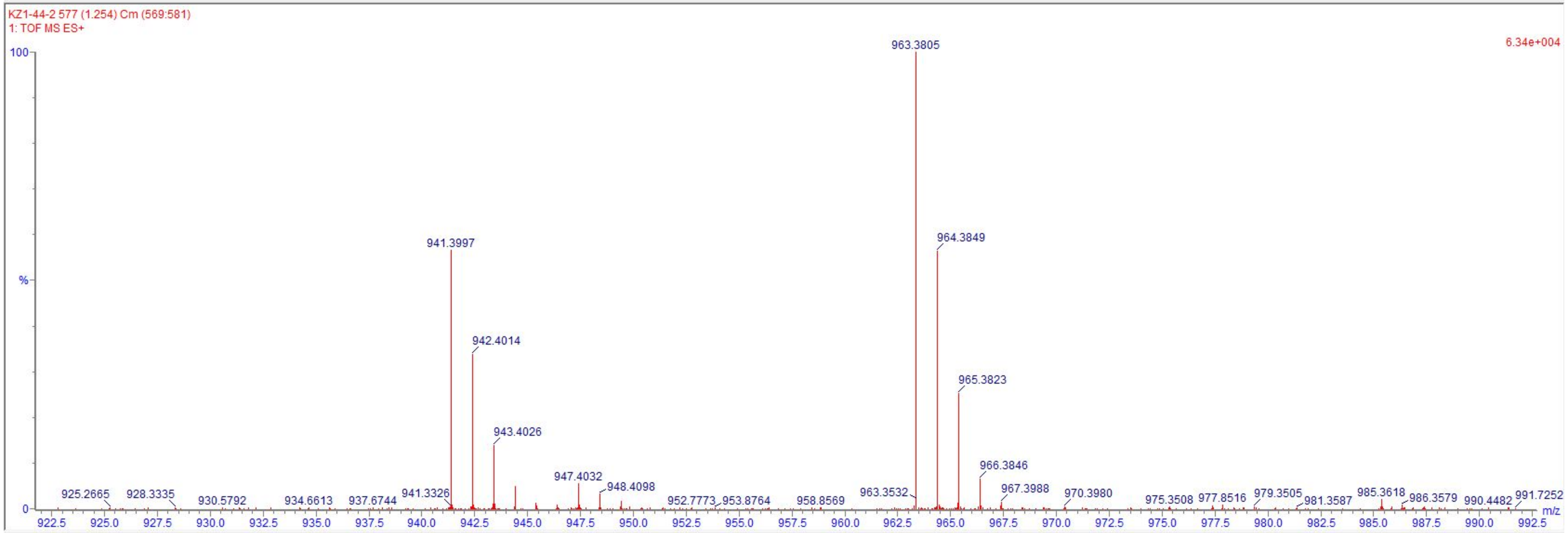

Fig. S27: HRMS of compound **16**

Multiple Mass Analysis: 4 mass(es) processed
Tolerance = 100.0 mDa / DBE: min = -10.0, max = 200.0
Element prediction: Off
Number of isotope peaks used for i-FIT = 3
Monoisotopic Mass, Even Electron Ions
262 formula(e) evaluated with 36 results within limits (up to 3 closest results for each mass)
Elements Used:

Mass	RA	Calc. Mass	mDa	PPM	DBE	Formula	i-FIT	i-FIT Norm	Fit Conf %	C	H	N	O	Na	Si
941.3999	94.35	941.3996	0.3	0.3	13.5	C40 H65 N6 O16 Si2	249.8	1.713	18.03	40	65	6	16		2
		941.3972	2.7	2.9	10.5	C38 H66 N6 O16 Na Si2	248.8	0.729	48.23	38	66	6	16	1	2
		941.4335	-33.6	-35.7	9.5	C39 H70 N6 O15 Na Si2	249.2	1.087	33.74	39	70	6	15	1	2
942.4027	53.34	942.4176	-14.9	-15.8	9.5	C39 H69 N5 O16 Na Si2	221.9	0.722	48.58	39	69	5	16	1	2
		942.4200	-17.3	-18.4	12.5	C41 H68 N5 O16 Si2	222.2	1.017	36.15	41	68	5	16		2
		942.3624	40.3	42.8	18.5	C43 H60 N5 O15 Si2	223.0	1.880	15.27	43	60	5	15		2
963.3811	100.00	963.3815	-0.4	-0.4	13.5	C40 H64 N6 O16 Na Si2	338.5	0.195	82.31	40	64	6	16	1	2
		963.3839	-2.8	-2.9	16.5	C42 H63 N6 O16 Si2	341.3	3.031	4.83	42	63	6	16		2
		963.4179	-36.8	-38.2	12.5	C41 H68 N6 O15 Na Si2	340.3	2.051	12.86	41	68	6	15	1	2
964.3859	55.50	964.4019	-16.0	-16.6	12.5	C41 H67 N5 O16 Na Si2	287.7	0.895	40.86	41	67	5	16	1	2
		964.4043	-18.4	-19.1	15.5	C43 H66 N5 O16 Si2	288.0	1.112	32.88	43	66	5	16		2
		964.3468	39.1	40.5	21.5	C45 H58 N5 O15 Si2	288.2	1.337	26.26	45	58	5	15		2

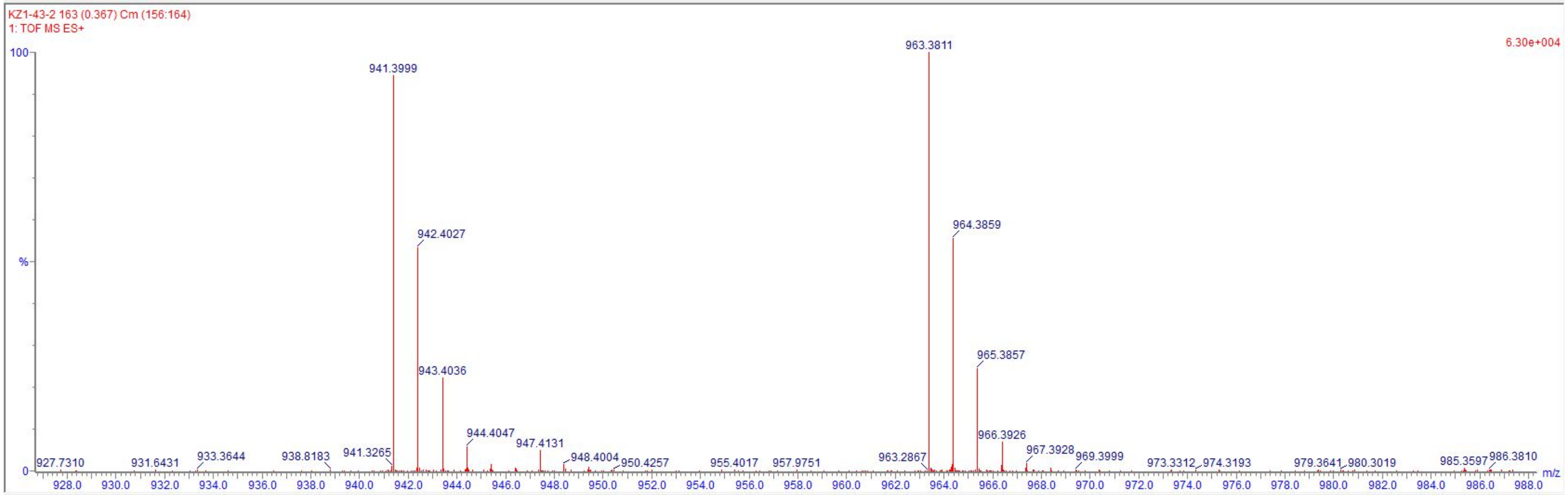

Fig. S28: HRMS of compound **17**

Multiple Mass Analysis: 2 mass(es) processed
Tolerance = 10.0 mDa / DBE: min = -100.0, max = 200.0
Element prediction: Off
Number of isotope peaks used for i-FIT = 3
Monoisotopic Mass, Even Electron Ions
338 formula(e) evaluated with 1 results within limits (up to 3 closest results for each mass)
Elements Used:

Mass	RA	Calc. Mass	mDa	PPM	DBE	Formula	i-FIT	i-FIT Norm	Fit Conf %	C	H	N	O	Na	Si
976.3769	100.00	976.3768	0.1	0.1	14.5	C40 ...	2...	n/a	n/a	40	63	7	16	1	2
977.3757	65.44	---													

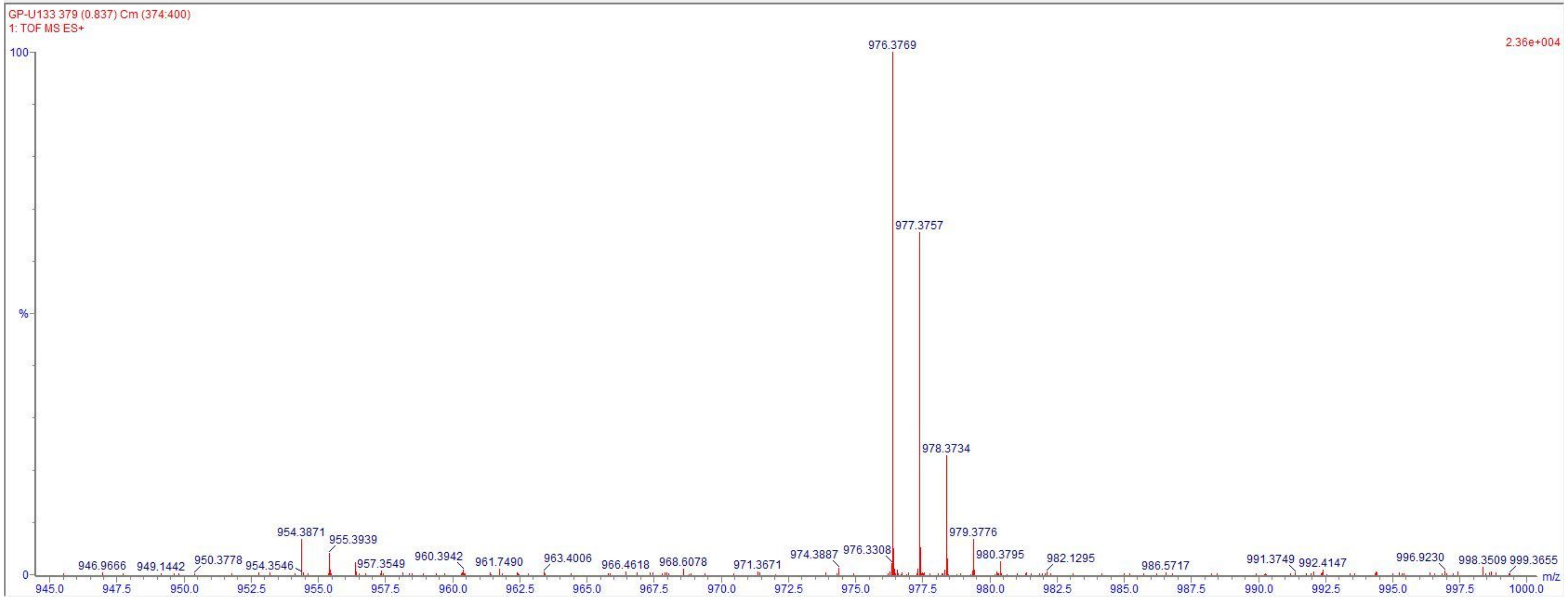

Fig. S29: HRMS of compound **18**

Tolerance = 10.0 mDa / DBE: min = -100.0, max = 200.0
Element prediction: Off
Number of isotope peaks used for i-FIT = 3
Monoisotopic Mass, Even Electron Ions
169 formula(e) evaluated with 1 results within limits (up to 3 closest results for each mass)
Elements Used:

Mass	RA	Calc. Mass	mDa	PPM	DBE	Formula	i-FIT	i-FIT Norm	Fit Conf %	C	H	N	O	Na	Si
976.3773	100.00	976.3768	0.5	0.5	14.5	C40 H63 N7 O16 Na Si2	33.4	n/a	n/a	40	63	7	16	1	2

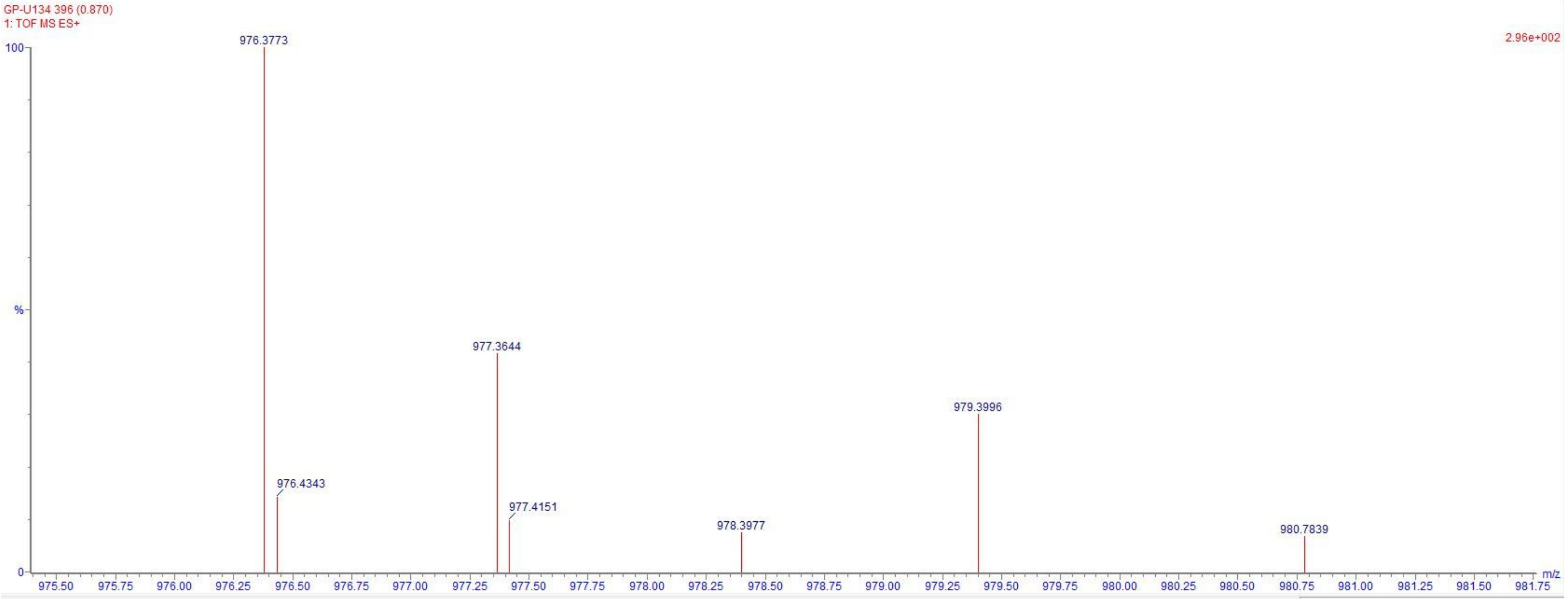

Fig. S30: HRMS of compound **19**

Multiple Mass Analysis: 2 mass(es) processed
Tolerance = 50.0 mDa / DBE: min = -10.0, max = 50.0
Element prediction: Off
Number of isotope peaks used for i-FIT = 2
Monoisotopic Mass, Even Electron Ions
593 formula(e) evaluated with 51 results within limits (up to 5 closest results for each mass)
Elements Used:

Mass	RA	Calc. Mass	mDa	PPM	DBE	Formula	i-FIT	i-FIT Norm	Fit Conf %	C	H	N	O	Si	S
900.3585	100.00	900.3561	2.4	2.7	21.5	C47 H58 N3 O11 Si S	89.3	0.341	71.13	47	58	3	11	1	1
		900.3593	-0.8	-0.9	16.5	C43 H62 N3 O12 Si2 S	90.3	1.307	27.05	43	62	3	12	2	1
		900.3553	3.2	3.6	12.5	C38 H62 N5 O14 Si2 S	93.1	4.114	1.63	38	62	5	14	2	1
		900.3534	5.1	5.7	25.5	C50 H58 N3 O7 Si2 S	95.8	6.860	0.10	50	58	3	7	2	1
		900.3589	-0.4	-0.4	17.5	C44 H58 N3 O15 S	96.2	7.211	0.07	44	58	3	15		1

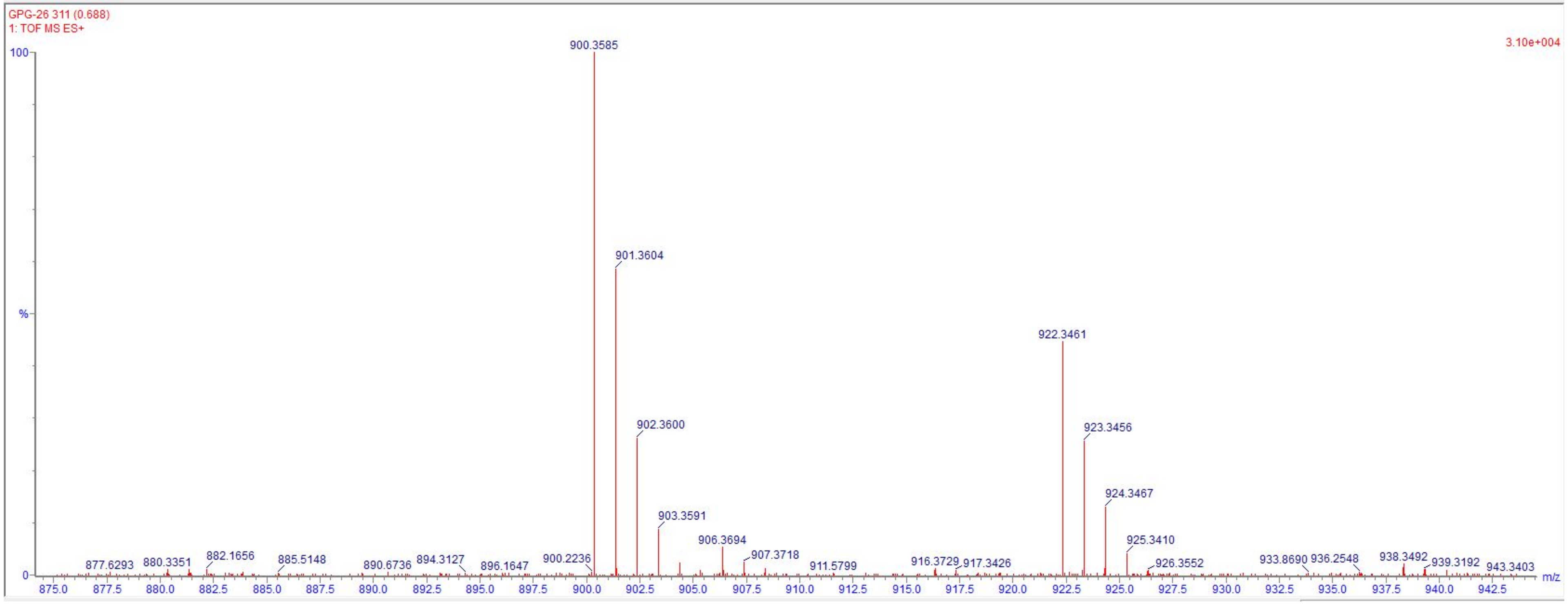

Fig. S31: HRMS of compound **20**

Multiple Mass Analysis: 2 mass(es) processed
Tolerance = 100.0 mDa / DBE: min = -10.0, max = 50.0
Element prediction: Off
Number of isotope peaks used for i-FIT = 2
Monoisotopic Mass, Even Electron Ions
1174 formula(e) evaluated with 238 results within limits (up to 5 closest results for each mass)
Elements Used:

Mass	RA	Calc. Mass	mDa	PPM	DBE	Formula	i-FIT	i-FIT Norm	Fit Conf %	C	H	N	O	S	Si
900.3560	100.00	900.3553	0.7	0.8	12.5	C38 H62 N5 O14 S Si2	93.7	0.566	56.76	38	62	5	14	1	2
		900.3561	-0.1	-0.1	21.5	C47 H58 N3 O11 S Si	94.3	1.163	31.26	47	58	3	11	1	1
		900.3593	-3.3	-3.7	16.5	C43 H62 N3 O12 S Si2	95.4	2.320	9.82	43	62	3	12	1	2
		900.3589	-2.9	-3.2	17.5	C44 H58 N3 O15 S	97.1	3.989	1.85	44	58	3	15	1	
		900.3534	2.6	2.9	25.5	C50 H58 N3 O7 S Si2	98.9	5.794	0.30	50	58	3	7	1	2
901.3622	56.66	901.3613	0.9	1.0	16.5	C44 H61 N2 O14 S Si	88.3	1.125	32.45	44	61	2	14	1	1
		901.3644	-2.2	-2.4	11.5	C40 H65 N2 O15 S Si2	88.4	1.268	28.15	40	65	2	15	1	2
		901.3595	2.7	3.0	26.5	C49 H53 N6 O9 S	88.8	1.664	18.95	49	53	6	9	1	

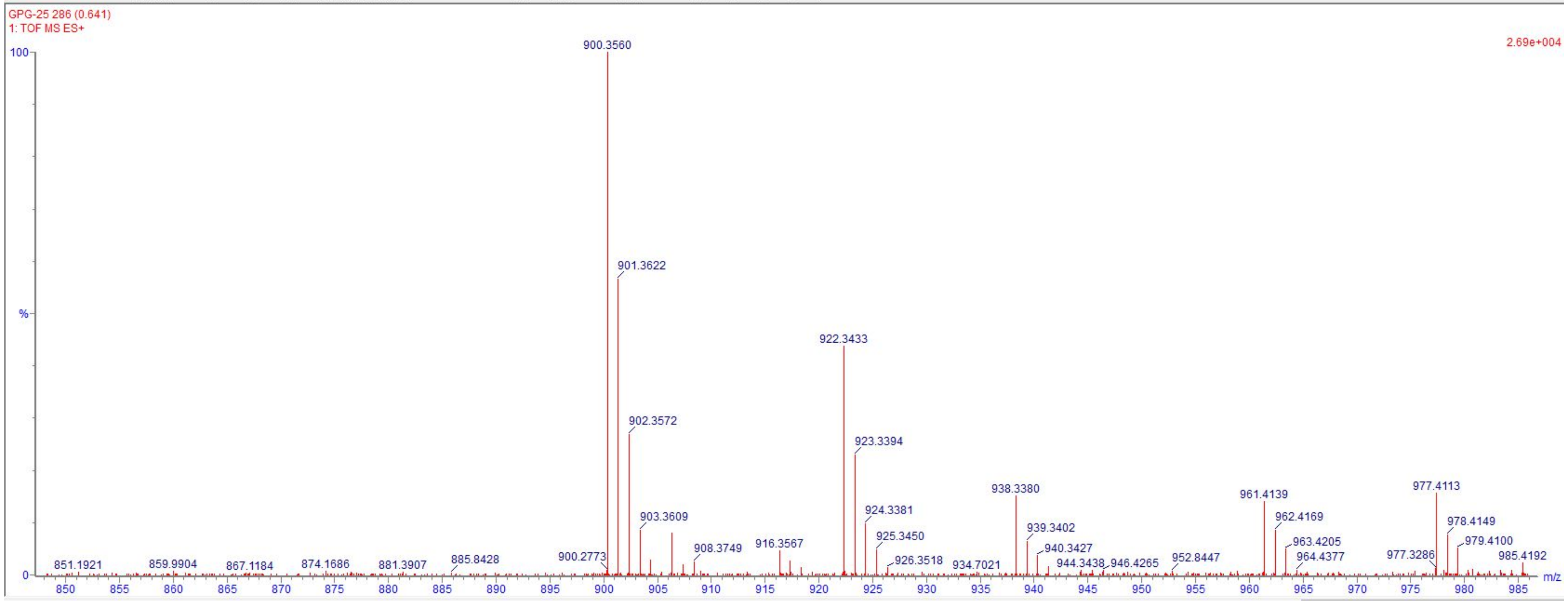

Fig. S32: HRMS of compound **21**

Tolerance = 100.0 mDa / DBE: min = -10.0, max = 200.0
Element prediction: Off
Number of isotope peaks used for i-FIT = 3
Monoisotopic Mass, Even Electron Ions
8131 formula(e) evaluated with 1074 results within limits (up to 5 closest results for each mass)
Elements Used:

Mass	RA	Calc. Mass	mDa	PPM	DBE	Formula	i-FIT	i-FIT Norm	Fit Conf %	C	H	N	O	Na	S	Si
965.3432	100.00	965.3430	0.2	0.2	13.5	C39 H62 N6 O15 Na S Si2	378.7	0.006	99.40	39	62	6	15	1	1	2
		965.3432	0.0	0.0	13.5	C40 H62 N6 O14 Na S2 Si	383.8	5.139	0.59	40	62	6	14	1	2	1
		965.3429	0.3	0.3	20.5	C45 H61 N6 O10 S2 Si2	387.8	9.185	0.01	45	61	6	10		2	2
		965.3432	0.0	0.0	30.5	C54 H53 N4 O11 S	390.4	11.725	0.00	54	53	4	11		1	
		965.3431	0.1	0.1	40.5	C60 H50 N6 O2 Na Si2	392.9	14.273	0.00	60	50	6	2	1		2

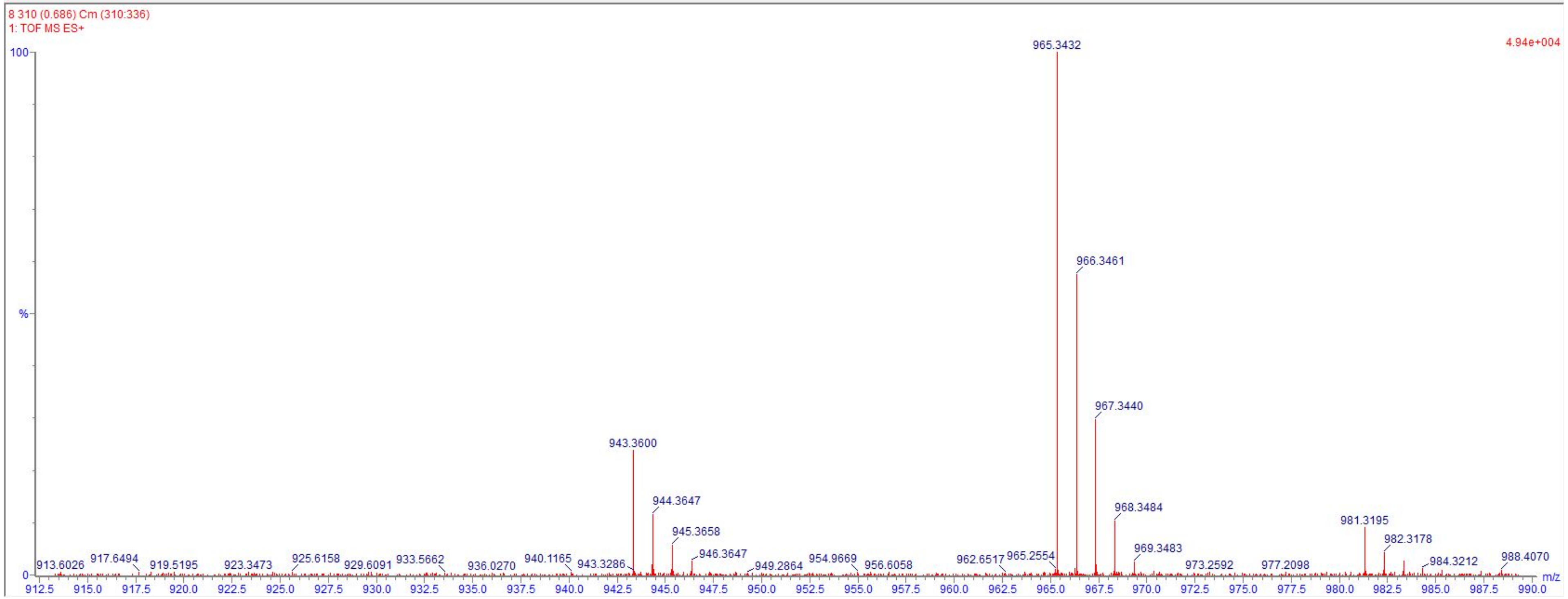

Fig. S33: HRMS of compound **22**

Tolerance = 100.0 mDa / DBE: min = -10.0, max = 200.0
Element prediction: Off
Number of isotope peaks used for i-FIT = 3
Monoisotopic Mass, Even Electron Ions
8131 formula(e) evaluated with 1073 results within limits (up to 5 closest results for each mass)
Elements Used:

Mass	RA	Calc. Mass	mDa	PPM	DBE	Formula	i-FIT	i-FIT Norm	Fit Conf %	C	H	N	O	Na	Si	S
965.3433	100.00	965.3430	0.3	0.3	13.5	C39 H62 N6 O15 Na Si2 S	335.6	0.042	95.89	39	62	6	15	1	2	1
		965.3432	0.1	0.1	13.5	C40 H62 N6 O14 Na Si S2	338.7	3.198	4.08	40	62	6	14	1	1	2
		965.3436	-0.3	-0.3	29.5	C53 H57 N4 O8 Si2 S	344.2	8.627	0.02	53	57	4	8		2	1
		965.3432	0.1	0.1	30.5	C54 H53 N4 O11 S	345.5	9.976	0.00	54	53	4	11			1
		965.3431	0.2	0.2	40.5	C60 H50 N6 O2 Na Si2	347.3	11.727	0.00	60	50	6	2	1	2	

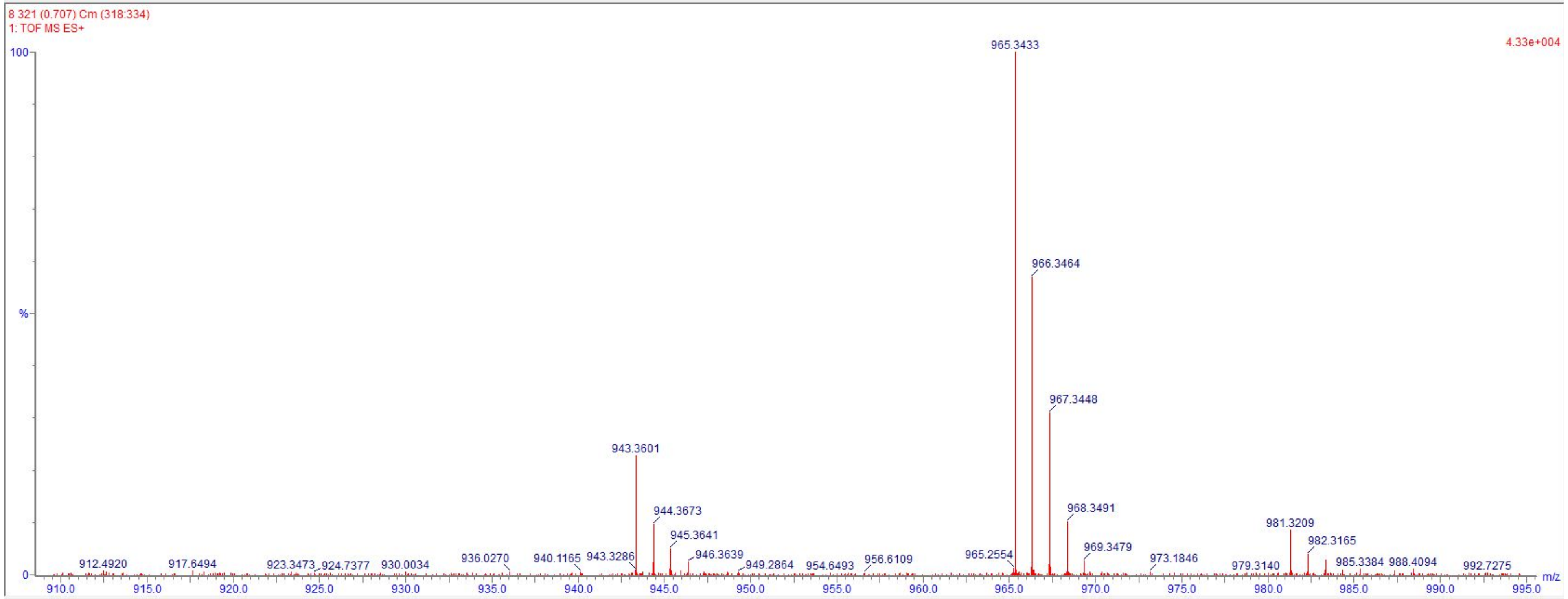

Fig. S34: HRMS of compound **23**

Tolerance = 100.0 mDa / DBE: min = -10.0, max = 200.0
Element prediction: Off
Number of isotope peaks used for i-FIT = 3
Monoisotopic Mass, Odd and Even Electron Ions
168 formula(e) evaluated with 41 results within limits (up to 4 closest results for each mass)
Elements Used:

Mass	RA	Calc. Mass	mDa	PPM	DBE	Formula	i-FIT	i-FIT Norm	Fit Conf %	C	H	N	O	Na	Si	S
952.3474	100.00	952.3478	-0.4	-0.4	12.5	C39 H63 N5 O15 Na Si2 S	446.5	0.014	98.63	39	63	5	15	1	2	1
		952.3468	0.6	0.6	20.5	C44 H58 N5 O15 Si2	453.3	6.842	0.11	44	58	5	15		2	
		952.3502	-2.8	-2.9	15.5	C41 H62 N5 O15 Si2 S	450.9	4.459	1.16	41	62	5	15		2	1
		952.3444	3.0	3.2	17.5	C42 H59 N5 O15 Na Si2	453.3	6.862	0.10	42	59	5	15	1	2	

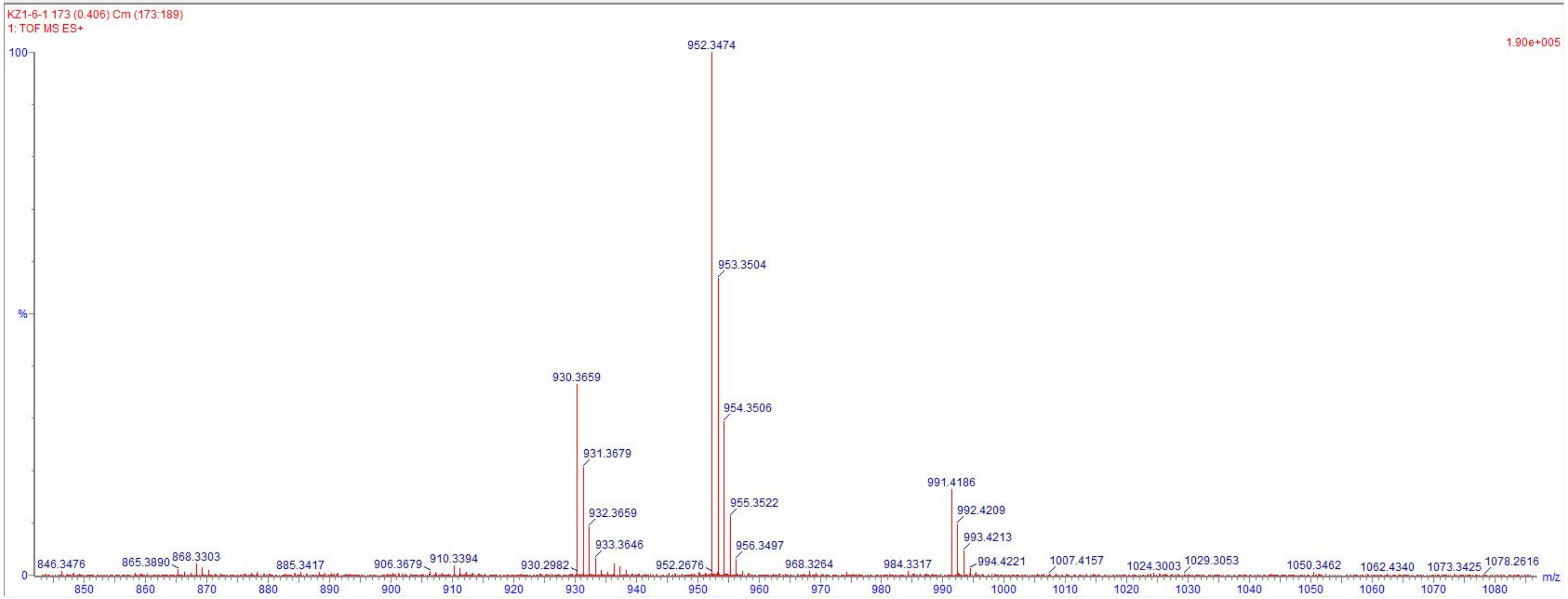

Fig. S35: HRMS of compound **24**

Tolerance = 100.0 mDa / DBE: min = -10.0, max = 200.0
Element prediction: Off
Number of isotope peaks used for i-FIT = 3
Monoisotopic Mass, Even Electron Ions
168 formula(e) evaluated with 42 results within limits (up to 4 closest results for each mass)
Elements Used:

Mass	RA	Calc. Mass	mDa	PPM	DBE	Formula	i-FIT	i-FIT Norm	Fit Conf %	C	H	N	O	Na	Si	S
952.3477	100.00	952.3478	-0.1	-0.1	12.5	C39 H63 N5 O15 Na Si2 S	67.0	0.984	37.37	39	63	5	15	1	2	1
		952.3468	0.9	0.9	20.5	C44 H58 N5 O15 Si2	69.4	3.397	3.35	44	58	5	15		2	
		952.3502	-2.5	-2.6	15.5	C41 H62 N5 O15 Si2 S	66.6	0.569	56.63	41	62	5	15		2	1
		952.3444	3.3	3.5	17.5	C42 H59 N5 O15 Na Si2	69.6	3.630	2.65	42	59	5	15	1	2	

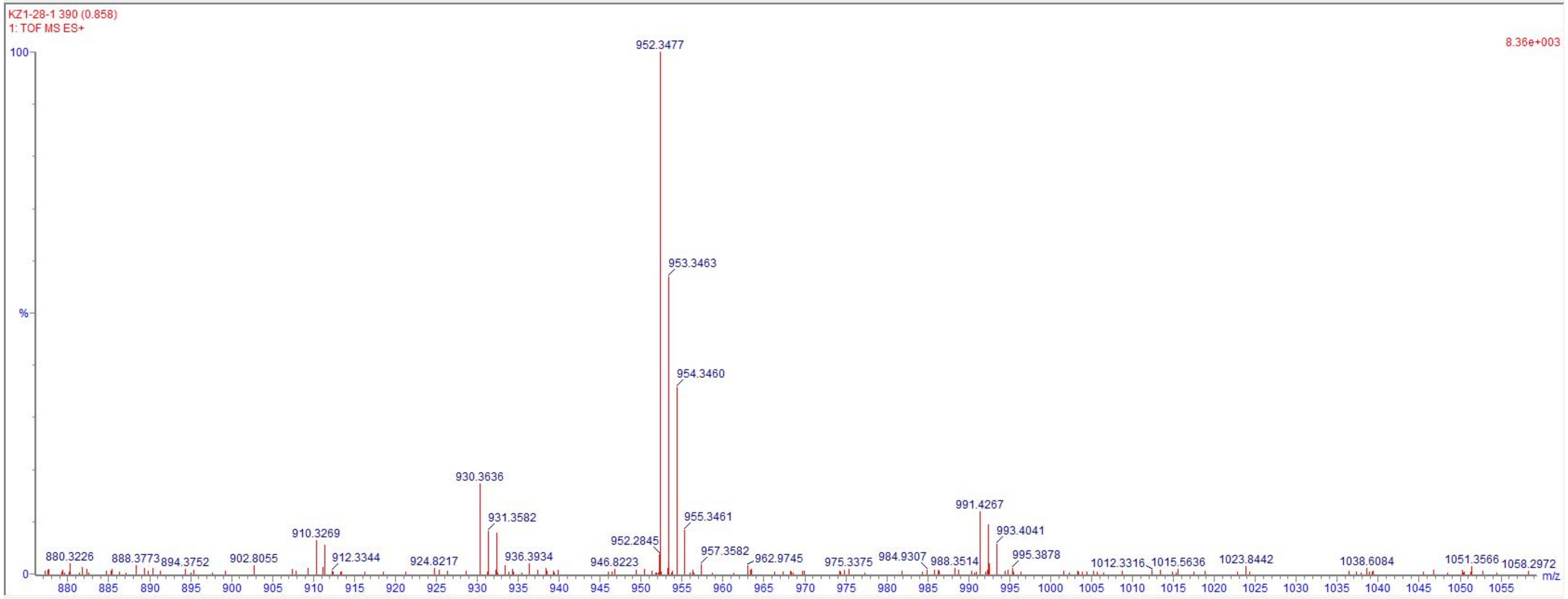

Fig. S36: HRMS of compound **25**

3. ***Western blot analysis of S, N and β-actin levels in SC-VLP producing cells.***

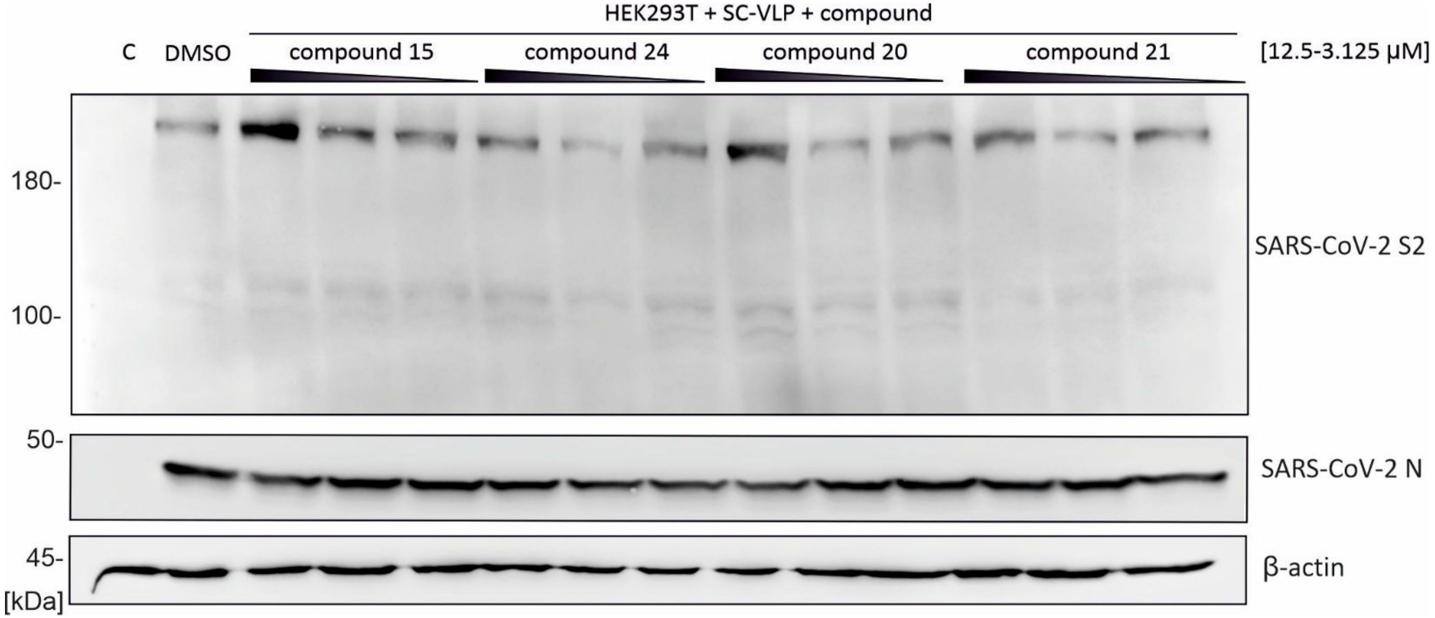

Fig. S37: ***Western blot analysis of S, N and β-actin levels in SC-VLP producing cells. C-non-transfected cells***
